# Constructing 3-Dimensional Atomic-Resolution Models of Nonsulfated Glycosaminoglycans with Arbitrary Lengths Using Conformations from Molecular Dynamics

**DOI:** 10.3390/ijms21207699

**Published:** 2020-10-18

**Authors:** Elizabeth K. Whitmore, Devon Martin, Olgun Guvench

**Affiliations:** 1 Department of Pharmaceutical Sciences and Administration, University of New England School of Pharmacy, 716 Stevens Avenue, Portland, ME 04103, USA; ewhitmore@une.edu (E.K.W.); dmartin11@une.edu (D.M.); 2 Graduate School of Biomedical Science and Engineering, University of Maine, 5775 Stodder Hall, Orono, ME 04469, USA

**Keywords:** molecular dynamics, glycosaminoglycan, proteoglycan, hyaluronan, dermatan, keratan, heparan, carbohydrate conformation, carbohydrate flexibility, glycosidic linkage, ring pucker, force field, explicit solvent

## Abstract

Glycosaminoglycans (GAGs) are the linear carbohydrate components of proteoglycans (PGs) and are key mediators in the bioactivity of PGs in animal tissue. GAGs are heterogeneous, conformationally complex, and polydisperse, containing up to 200 monosaccharide units. These complexities make studying GAG conformation a challenge for existing experimental and computational methods. We previously described an algorithm we developed that applies conformational parameters (i.e., all bond lengths, bond angles, and dihedral angles) from molecular dynamics (MD) simulations of nonsulfated chondroitin GAG 20-mers to construct 3-D atomic-resolution models of nonsulfated chondroitin GAGs of arbitrary length. In the current study, we applied our algorithm to other GAGs, including hyaluronan and nonsulfated forms of dermatan, keratan, and heparan and expanded our database of MD-generated GAG conformations. Here, we show that individual glycosidic linkages and monosaccharide rings in 10- and 20-mers of hyaluronan and nonsulfated dermatan, keratan, and heparan behave randomly and independently in MD simulation and, therefore, using a database of MD-generated 20-mer conformations, that our algorithm can construct conformational ensembles of 10- and 20-mers of various GAG types that accurately represent the backbone flexibility seen in MD simulations. Furthermore, our algorithm efficiently constructs conformational ensembles of GAG 200-mers that we would reasonably expect from MD simulations.

## 1. Introduction

Proteoglycans (PGs) are a diverse group of proteins in the extracellular matrix (ECM), as well as on and within cells in animal tissue. PGs play key roles in signal transduction [1,2], tissue morphogenesis [3,4,5,6,7], and matrix assembly [7,8,9,10] by binding growth factors [3,4,5,6,7,11,12,13,14,15,16,17], enzymes [7,17], membrane receptors [17], and ECM molecules [2,7,17]. Many of these functions are mediated by glycosaminoglycans (GAGs), which are linear, highly negatively-charged, and structurally diverse carbohydrate chains covalently-linked to PGs. Specifically, GAGs can form covalent and noncovalent complexes with proteins or inhibit complex formation with other biomolecules. All of these functions allow GAGs to modulate disease. For example, hyaluronan can predict disease outcome and is used as a treatment for osteoarthritis [18,19]; deficiency of dermatan sulfate has been linked to Ehlers-Danlos syndrome, so screening of dermatan sulfate in urine may present a method of diagnosis [20,21]; reduced cerebral cell keratan sulfate levels have been implicated in Alzheimer’s disease [22]; and heparan sulfate has been shown to induce septic shock [23,24].

GAG-protein binding is determined by the sequence, structure, shape, and charge of the GAG and GAG-binding region of the protein [17,25]. Therefore, the three-dimensional structure and conformation of GAGs determine their bioactivity, and even subtle structural differences can change their function. For example, while hyaluronan and chondroitin have many functional differences, the only structural difference is in the chirality of carbon four in the amide monosaccharide derivatives (i.e., N-acetylglucosamine and N-acetylgalactosamine, respectively). The structural and conformational complexities of GAGs make studying GAG conformational thermodynamics at atomic resolution a challenge using existing experimental methods. Structural complexities arise from nontemplate-based synthesis [26] and variable sulfation [27], which means a single GAG type can have variable lengths and sulfation patterns, making it polydisperse and heterogeneous. Conformational complexities result from glycosidic linkage flexibility [28,29,30,31,32,33] and ring puckering [33]. There are several experimental techniques for studying GAGs, including liquid chromatography-mass spectrometry (LC-MS) [34,35,36], X-ray crystallography [37,38,39,40,41,42], and nuclear magnetic resonance (NMR) [43,44,45,46], but no single technique is able to account for all of these complexities. While each has its limitations, these experimental techniques provide a critical means to validate molecular dynamics (MD) simulations [46,47,48,49,50,51,52]. MD can be used to generate atomic-resolution conformational ensembles of GAGs under physiological conditions, and agreement between MD-generated results and available experimental data builds confidence that MD does indeed generate realistic three-dimensional atomic-resolution GAG models [53,54,55,56,57]. However, all-atom explicit-solvent MD is not a feasible tool for routine simulation of large systems, and GAG biopolymers may consist of up to 200 monosaccharide units [4], which, when fully solvated, would result in a system with over 10^6^ atoms. Coarse-grained (CG) MD is a feasible alternative for simulations of long GAG polymers, but may be limited because it lacks the atomic resolution and the accurate treatment of solvation provided by all-atom explicit-solvent MD [48,58,59,60].

As an alternative approach to obtaining atomic-resolution, three-dimensional, conformational ensembles of long GAG biopolymers, we developed an algorithm that uses glycosidic linkage and monosaccharide ring conformations from unbiased all-atom explicit-solvent MD simulations [57,61,62,63] of GAG 20-mers (i.e., 20 monosaccharide units) to rapidly generate GAG polymer conformational ensembles of user-specified length and with a user-specified number of conformations [33]. In our previous work, we examined nonsulfated chondroitin GAGs. Here, we aim to expand the application of our algorithm to hyaluronan and to nonsulfated forms of dermatan, keratan, and heparan. Nonsulfated forms were chosen for their simplicity and homogeneity. The algorithm is expected to produce conformational ensembles that mimic the backbone flexibility observed in simulations only if individual ring and linkage conformations of GAG 20-mers behave randomly and independently in simulation. This means that the algorithm will not produce polymer conformations with higher-order structures.

It has been suggested that hyaluronan may take on higher order structures [64], and while it has been shown that helical structures occur in hyaluronan oligosaccharides [65,66], this behavior may not necessarily be extrapolated to long polysaccharides under physiological conditions. Hyaluronan oligosaccharides have been shown to exhibit rigid structures in aqueous solution, possibly resulting from electrostatic effects and hydrogen-bonding between neighboring monosaccharides [65,67,68,69,70,71,72,73]. MD studies of hyaluronan di- and tri- saccharides in aqueous solution (without ions) revealed that hydrogen-bonds form but do not remain for long periods of time, and do not always occur at the same time in different regions of the oligosaccharide [67,68]. It has been shown that under physiological conditions, the extended conformations and large hydrodynamic radius of hyaluronan can be attributed primarily to hydrogen-bonding between adjacent monoasaccharides (and less so to electrostatic effects) [69]. It is important to note that each of these studies examined short hyaluronan oligosaccharides; thus, atomic-resolution conformational properties of long polysaccharides under physiological conditions have not been reported.

We sought to determine (1) if longer polysaccharides of hyaluronan and nonsulfated dermatan, keratan, and heparan took on a higher-order structure during MD simulations in an aqueous solution with physiological ion strength and, consequently, (2) if our algorithm could construct conformational ensembles of GAGs that mimic the backbone flexibility observed in MD simulations. To this end, we ran a microsecond-scale, all-atom, explicit-solvent MD on 20-mers of each GAG type with sequences [-4 glucuronate β1-3 N-acetylglucosamine β1-]_10_ (hyaluronan), [-4 iduronate α1-3 N-acetylgalactosamine β1-]_10_ (dermatan), [-3 galactose β1-4 N-acetylglucosamine β1-]_10_ (keratan), and [-4 iduronate α1-4 N-acetylglucosamine α1-]_10_ (heparan). Analysis of glycosidic linkage and monosaccharide ring conformations revealed no apparent correlation between individual rings and flanking linkages in any of the 20-mers. Glycosidic linkages took on experimentally-observed helical conformations, and some linkages sampled secondary conformations that caused a kink in the polymer. No patterns of interdependence between adjacent linkage conformations were seen. While ring and linkage conformations did not reach equilibrium in hyaluronan simulations on the microsecond timescale, overall hyaluronan 20-mer appeared to behave randomly. End-to-end distance distributions revealed mostly extended conformations (n.b. we use the term “extended” throughout to describe conformations with high end-to-end distances; this does not necessarily contradict helical structure) with some compact conformations resulting from non-^4^C_1_ ring puckering, which brought the oxygen atoms in sequential glycosidic linkages closer together, and certain glycosidic linkage conformations that brought neighboring monosaccharides closer together. Thus, these ring and linkage conformations both introduce a kink in the polymer. These conformations are independent and occur rarely on the microsecond timescale. The variability in individual ring and linkage conformations, as well as end-to-end distances and the radii of gyration, demonstrate the flexibility and lack of stable higher-order structures of each of these nonsulfated GAG polymers during simulation in aqueous solution.

For each nonsulfated GAG type, monosaccharide ring and glycosidic linkage conformations from MD-generated 20-mers were used as inputs in our algorithm to generate conformational ensembles of 10-, 20-, and 200-mers. For each GAG type, 10- and 20-mer constructed ensembles were compared to 10- and 20-mer MD-generated ensembles. In each case, end-to-end distances in constructed and MD-generated ensembles match, with minimal differences in most probable end-to-end distances. The similarities in backbone conformation of constructed and MD-generated 10- and 20-mer ensembles provide further evidence that there is little correlation between individual glycosidic linkage and ring conformations, and thus, that nonsulfated GAG 10- and 20-mers do not exhibit a higher-order structure. This suggests that our program can be used to construct realistic conformations of long GAG biopolymers (e.g., 200 monosaccharides), and the resulting atomic-resolution models may be used to develop further insights into GAG binding properties, and thus, GAG-protein complex formation. These will be especially useful for studying complexes in which there are multiple biomolecules bound to the same GAG.

Other programs exist which are capable of constructing three-dimensional, atomic-resolution GAG polymer models including Glycam GAG Builder [74], POLYS Glycan Builder [75], CarbBuilder [76], MatrixDB GAG Builder [77,78], and CHARMM-GUI [79,80,81,82]. These are useful tools for constructing the initial structures of GAGs having user-specified sequences and lengths for MD simulations. Additional features of Glycam and POLYS Glycan Builder include user specification of glycosidic linkage dihedral angle values or construction using conformations from their databases. Glycam, POLYS Glycan Builder, and Carb Builder databases consist of GAG mono- and di- saccharide structures sampled in MD and/or determined by molecular mechanics calculations. The MatrixDB databases contain GAG disaccharide conformations from GAG-protein complex crystal structures. CHARMM-GUI uses the CHARMM software [83,84,85] to construct GAGs with user-specified sequences, using default force field parameters for carbohydrates [57,61,62,63], adding explicit solvents with user-specified water box size and ion concentrations, and generating inputs for MD equilibration and production simulations. Although the user has the option to choose the GAG length, these tools are best used for short GAG oligosaccharides. Our algorithm differs from these tools in that it draws upon our databases containing full MD-generated unbound GAG 20-mer atomic-resolution conformational ensembles. Furthermore, our algorithm is developed for modeling long GAG biopolymers (e.g., 200-mers), and can efficiently produce large conformational ensembles (e.g., 10,000 3-D models) of long GAG biopolymers. Therefore, our algorithm provides an alternative to MD simulation that takes much less time and computational resources.

## 2. Materials and Methods

The following protocol was conducted for each of the hyaluronan and nonsulfated dermatan, keratan, and heparan GAGs.

### 2.1. Molecular Dynamics: System Construction, Energy Minimization, Heating, and Production Simulations

System construction, energy minimization, and heating followed the same protocol we described previously [33]. Briefly, all polymers were constructed with an explicit solvent using the CHARMM software [83,84,85] v. c41b2 with the CHARMM36 (C36) biomolecular force field for carbohydrates [57,61,62,63]. The initial conformation of each GAG 10- and 20-mer had glycosidic linkage dihedrals (with IUPAC definitions: *ϕ* = O_5_-C_1_-O-C*_n_* and *ψ* = C_1_-O-C*_n_*-C_(*n*−1)_) set to *ϕ* = −83.75° and *ψ* = −156.25° in all GlcAβ1-3GlcNAc (hyaluronan), IdoAα1-3GalNAc (dermatan), and Galβ1-4GlcNAc (keratan) linkages and *ϕ* = −63.75° and *ψ* = 118.75° in all GlcNAcβ1-4GlcA (hyaluronan), GalNAcβ1-4IdoA (dermatan), and GlcNAcβ1-3Gal (keratan) linkages. These dihedral values were the most energetically stable in nonsulfated chondroitin disaccharide glycosidic linkages during MD simulation [55]. In heparan 10- and 20-mers, all IdoAα1-4GlcNAc glycosidic linkage dihedrals were set to *ϕ* = −69° and *ψ* = 147° and all GlcNAcα1-4IdoA linkage dihedrals were set to *ϕ* = 77° and *ψ* = 30°. These dihedral values were found in an NMR and force field study of heparan disaccharides [30], and gave extended heparan polymer conformations. With these glycosidic linkage conformations, 20-mer end-to-end distances were 99.0 Å, 89.7 Å, 85.8 Å, and 87.5 Å for hyaluronan, dermatan, keratan, and heparan, respectively. The GAG 20-mers were solvated in cubic periodic unit cells with edge lengths of 124.3 Å (63,347 water molecules), 115.0 Å (50,066 water molecules), 111.9 Å (46,103 water molecules), and 111.9 Å (46,122 water molecules) for hyaluronan, dermatan, keratan, and heparan, respectively. Each of these edge lengths is at least 10 Å longer than the maximum dimension of the corresponding 20-mer in a fully-extended (i.e., maximum end-to-end distance) conformation. The TIP3 water model [86,87], as implemented in the CHARMM force field, was used for all simulations, and neutralizing Na^+^ counterions and 140 mM NaCl [88] were added.

The NAMD program [89] v. 2.12 (http://www.ks.uiuc.edu/Research/namd/) was used to minimize the potential energy and heat the system to the target temperature of 310 K under constant pressure at 1 atm. An unbiased constant particle number/constant pressure/constant temperature (NPT) MD run followed, and average periodic cell parameters from the last half of this trajectory were used as cell basis vectors for the quadruplicate canonical (NVT) ensemble MD simulations. NVT MD simulations were preceded by minimization and heating at constant volume with box edge lengths of 123.7 Å, 114.4 Å, 111.3 Å, and 111.3 Å for hyaluronan, dermatan, keratan, and heparan, respectively.

Production simulations were performed using a similar protocol as previously described [33] but with a longer timescale and lower frequency for saving snapshots. Briefly, using the CHARMM software, each of four replicates (per GAG system) was equilibrated. Then, unbiased canonical (NVT) ensemble MD simulations were run for 500,000,000 steps (1 µs) and atomic coordinates were saved at 50,000-step (100-ps) intervals for analyses (10,000 snapshots per simulation, 40,000 snapshots per GAG).

### 2.2. Conformational Analysis

**Figure 1:**
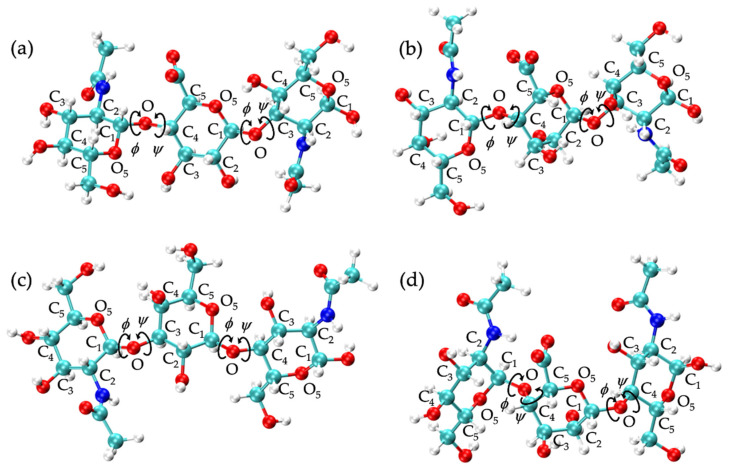
GAG trisaccharides: (**a**) hyaluronan [GlcNAcβ1-4GlcAβ1-3GlcNAc], (**b**) nonsulfated dermatan [GalNAcβ1-4IdoAα1-3GalNAc], (**c**) nonsulfated keratan [GlcNAcβ1-3Galβ1-4GlcNAc], (**d**) nonsulfated heparan [GlcNAcα1-4IdoAα1-4GlcNAc]; endocyclic ring atoms and oxygen atoms linking adjacent rings are labeled; arrows indicate rotations about glycosidic linkage bonds C_1_-O and O-C*_n_* (quantified by *ϕ* and *ψ* dihedral angles, respectively); glycosidic linkage parameters used in the construction algorithm include C_1_-O and O-C*_n_* bond lengths, O_5_-C_1_-O and C_1_-O-C*_n_* bond angles, and *ϕ* = O_5_-C_1_-O-C*_n_* and *ψ* = C_1_-O-C*_n_*-C_(_*_n_*_−__1)_ dihedral angles. Molecular graphics throughout are produced with the VMD program [90].

GAG conformational parameters include bond length, bond angle, and dihedral angle values. The glycosidic linkage conformational parameters considered were bond lengths for C_1_-O and O-C*_n_*, bond angles for O_5_-C_1_-O and C_1_-O-C*_n_*, and dihedral angles *ϕ* and *ψ* (Figure 1). To characterize potential patterns in glycosidic linkage conformations, dihedral angle free energies Δ*G*(*ϕ*, *ψ*) were analyzed. As *ϕ*, *ψ* dihedral values from MD-generated ensembles were taken to have uniform probabilities, Δ*G*(*ϕ*, *ψ*) was calculated by sorting dihedral angle values into 2.5° × 2.5° bins and then using the formula Δ*G*(*ϕ_i_*,*ψ_j_*) = −*RT*ln(*n_ij_*) − *k*, where *n_ij_* is the bin count for the bin corresponding to *ϕ_i_*,*ψ_j_*, *R* is the universal gas constant, *T* is the temperature of the MD simulations, and *k* was chosen so that the global minimum was located at Δ*G* = 0 kcal/mol.

Monosaccharide ring geometries are quantified by all bond lengths, bond angles, and dihedral angles within the ring and in exocyclic functional groups that are not part of a glycosidic linkage. Cremer-Pople (C-P) ring puckering parameters (*ϕ*, *θ*, *Q*) [91] of each monosaccharide ring in the MD-generated 20-mer ensembles were computed to characterize potential ring puckering patterns. Conformations of each monosaccharide and each linkage of the 20-mers were extracted separately from each snapshot of the MD trajectories. First, to determine if conformational data in different runs matched, and if there was interdependency in the individual linkage and ring conformations, data were separated by run and monosaccharide/linkage number and aggregated across all snapshots in each MD simulation run. Next, all individual conformations were aggregated across all snapshots in all runs (e.g., 10,000 snapshots * 4 runs * 10 GlcA monosaccharides = 400,000 samples of GlcA monosaccharide conformations in hyaluronan 20-mer) to generate a single dataset for each of the two monosaccharide types and two linkage types in each GAG (e.g., GlcA monosaccharide, GlcNAc monosaccharide, β1-3 glycosidic linkage, and β1-4 glycosidic linkage conformations in hyaluronan 20-mer).

### 2.3. Construction Algorithm to Generate GAG Conformational Ensembles

The MD-generated conformational datasets described above made up the database used for the algorithm we developed to generate GAG polymer conformational ensembles of user-specified length and with a user-specified number of conformations (previously described) [33]. Essentially, our algorithm (1) incorporates all bond, bond angle, and dihedral angle conformational parameters from MD simulation of GAG 20-mers, (2) treats monosaccharide rings and glycosidic linkages independently, (3) performs a restrained energy minimization on each constructed conformation to relieve steric overlap while maintaining polymer conformation, and (4) applies a bond potential energy cutoff to exclude conformations that remain nonphysical after minimization. A nonphysical conformation results from either overlapping bonds or a bond that pierces the center of another monosaccharide ring in the initial constructed conformation (Figure S1). As dihedral angles are restrained, minimization addresses these issues by stretching the overlapping or ring-piercing bonds to nonphysical lengths, which increases the post-minimization bond potential energy by more than 132 kcal/mol [33]. The energy cutoff is the sum of a 100 kcal/mol buffer and a polymer-length specific cutoff, which is equal to the post-minimization bond potential energy of the fully-extended conformation. This conformation was constructed by assigning energetically-favorable glycosidic linkage dihedrals from the corresponding MD simulations of each GAG type that give a fully-extended conformation (i.e., with the maximum end-to-end distance observed in simulation). We used the algorithm to construct 20-mer conformational ensembles of each nonsulfated GAG.

For internal validation of the algorithm, glycosidic linkage dihedral free energies ΔG(*ϕ*,*ψ*), monosaccharide ring C-P parameters, end-to-end distance distributions, and radii of gyration from MD-generated ensembles and constructed ensembles were compared. Additionally, post-minimization bond potential energy distributions from constructed ensembles were analyzed to validate the calculated bond potential energy cutoff and verify that the ensemble had expected energy distributions for the given polymer length.

To evaluate application of MD-generated 20-mer conformations to construct GAG polymers of variable length, we constructed 10-mer ensembles using the algorithm and compared them to 10-mer ensembles generated using the same protocol as 20-mer MD simulations. To assess the efficacy and efficiency of our algorithm to construct conformational ensembles of GAG polymers with biologically-relevant chain lengths, we also constructed 200-mer ensembles.

## 3. Results and Discussion

### 3.1. Hyaluronan

#### 3.1.1. Molecular Dynamics Simulations: Glycosidic Linkage and Monosaccharide Ring Geometry Effects on Polymer Backbone Flexibility

**Figure 2:**
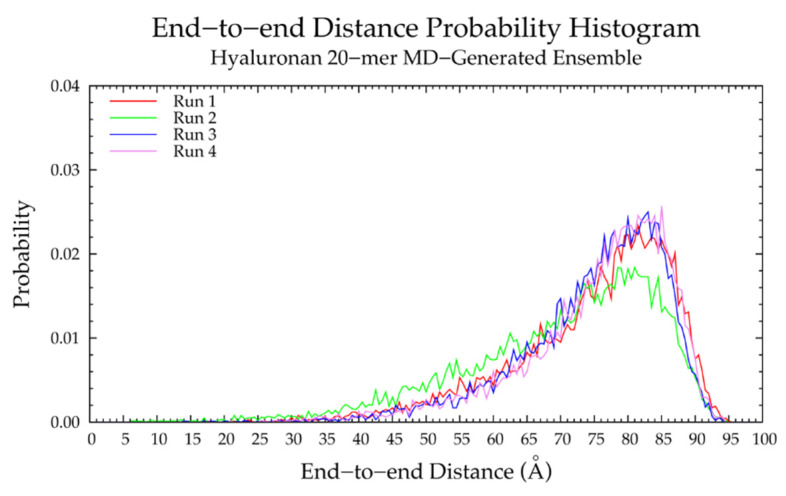
End-to-end distance probability distribution of MD-generated hyaluronan 20-mer ensemble; each of the four runs includes 10,000 conformations; probabilities were calculated for end-to-end distances sorted into 0.5 Å bins.

To examine the backbone flexibility of hyaluronan 20-mers, end-to-end distances and radii of gyration were analyzed in each of the four MD simulation runs. One of the four runs produced more compact conformations, i.e., with lower end-to-end distances and radii of gyration than the other three (Figure 2 and Figure S2a and Table 1). The system potential energy distributions were identical for all four runs, suggesting that this outlying run was energetically stable (Figure S3). To uncover the conformational factors that contributed to end-to-end distance and explain the differences in backbone flexibility in the different MD runs, the monosaccharide ring and glycosidic linkage conformations were examined.

**Figure 3:**
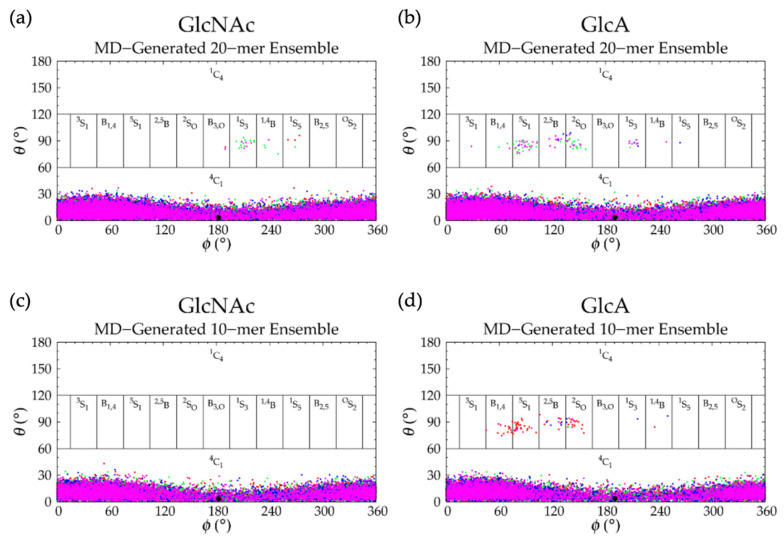
Cremer–Pople data for MD-generated hyaluronan ensembles: (**a**) GlcNAc and (**b**) GlcA in the 20-mer and (**c**) GlcNAc and (**d**) GlcA in the 10-mer; geometries from the four sets of each ensemble are represented by red, green, blue, and magenta dots, respectively and the force-field geometry is represented by a single large black dot; Cremer–Pople parameters (*ϕ*, *θ*) for all rings in every tenth snapshot from each ensemble were plotted (i.e., 10 rings × 1000 snapshots per run × 4 runs = 40,000 parameter sets for the 20-mer and 20,000 parameter sets for the 10-mer).

**Figure 4:**
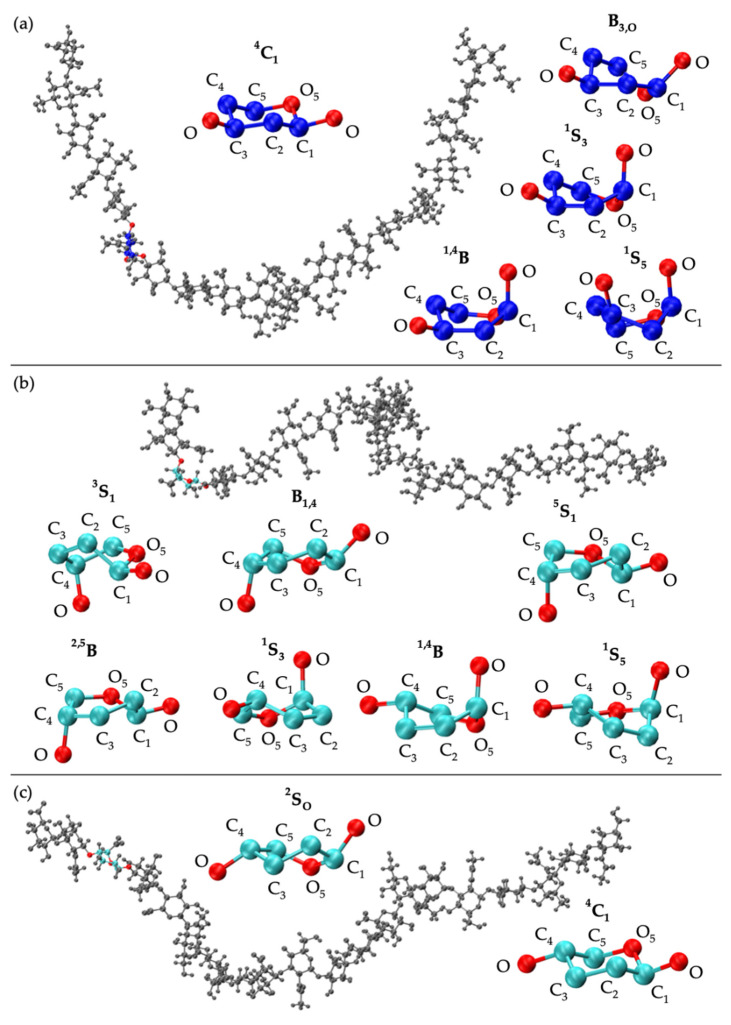
MD-generated hyaluronan conformations: (**a**) 20-mer with a ^1,4^B GlcNAc conformer (ring atoms in blue; linkage atoms in red) and ^4^C_1_, B_3,O_, ^1^S_3_, ^1,4^B, and ^1^S_5_ GlcNAc monosaccharide conformations (endocyclic ring atoms and linker oxygen atoms only); (**b**) 20-mer with a ^5^S_1_ GlcA conformer (ring atoms in cyan; linkage atoms in red) and ^3^S_1_, B_1,4_, ^5^S_1_, ^2,5^B, ^1^S_3_, ^1,4^B, and ^1^S_5_ GlcA monosaccharide conformations (endocyclic ring atoms and linker oxygen atoms only); (**c**) 20-mer with a ^2^S_O_ GlcA conformer (ring atoms in cyan; linkage atoms in red) and ^4^C_1_ and ^2^S_O_ GlcA monosaccharide conformations (endocyclic ring atoms and linker oxygen atoms only); in each 20-mer snapshot, all gray rings are in ^4^C_1_ conformations.

Analysis of C-P parameters of GlcNAc monosaccharide rings revealed mostly ^4^C_1_ chair conformations, as found in crystal structures [92,93,94,95,96,97,98,99] and NMR and force field studies [67,100,101,102], with a minority of samples in boat and skew-boat conformations, namely B_3,O_, ^1^S_3_, ^1,4^B, and ^1^S_5_ (Figure 3a and Figure 4a). While non-^4^C_1_ conformations of GlcNAc are rare, they have been observed in crystal structures of protein co-complexes [95,103,104,105,106]. For example, one GlcNAc ring in the crystal structure of a hyaluronan tetramer in complex with hyaluronidase was found in the ^1,4^B boat conformation [95]. Additionally, GlcNAc boat/skew-boat conformations have been sampled in biased MD [107], 1-µs unbiased MD [108], and 10-µs unbiased MD simulations of GlcNAc monosaccharides [100]. One NMR and force field study suggested that these conformations were important for GAG-protein interactions [100]. Analysis of individual GlcNAc rings in each run revealed that these non-^4^C_1_ conformations were not specific to any particular region of the 20-mer and did not occur simultaneously in multiple rings in the same snapshot (Figure S4). Furthermore, GlcNAc rings that sampled non-^4^C_1_ puckers returned to ^4^C_1_ chair conformations relatively quickly, i.e., in under 25 ns (Figure S4).

C-P analysis of GlcA monosaccharide rings in the hyaluronan 20-mer showed that GlcA is found mostly in ^4^C_1_ chair conformations (Figure 3b), as seen in crystal structures [92,93,94,95] and NMR and force field studies [51,67,101,102,107,109,110], with some boat and skew-boat conformations, including ^3^S_1_, B_1,4_, ^5^S_1_, ^2,5^B, ^2^S_O_, ^1^S_3_, ^1,4^B, and ^1^S_5_ (Figure 3b and Figure 4b,c), which were also observed in GlcA rings in unbiased MD simulations of nonsulfated chondroitin 20-mers [33]. Some of these conformations were also sampled rarely in unbiased MD simulations of GlcA monosaccharides [51,107]. The slight differences between boat/skew-boat conformations in our simulations and monosaccharide simulations may have arisen from intramolecular interactions in the hyaluronan 20-mer. As with GlcNAc rings, these conformations did not occur consistently in any one region of the 20-mer, did not occur simultaneously in multiple rings in the same snapshot, and returned to ^4^C_1_ chair conformations quickly, i.e., within 40 ns (Figure S5). Except for ^2^S_O_, each of these GlcA and GlcNAc boat and skew-boat conformations brought the linker oxygen atoms closer together, causing a kink in the polymer chain (Figure 4), which may have contributed to more compact polymer conformations. To investigate this, end-to-end distances and radii of gyration of 20-mer conformations with non-^4^C_1_ ring puckers were analyzed (Figure S6) and both compact and extended conformations were observed. This can be explained by the flexibility in the glycosidic linkages. Of note, MD-generated atomic-resolution snapshots before and after a monosaccharide ring underwent a conformational change between ^4^C_1_ and non-^4^C_1_ were visualized in VMD to check for ionic interactions (e.g., between Na^+^ and carboxylate O^−^ atoms and/or partially-charged hydroxyl O atoms in GlcA, between Cl^−^ and hydroxyl H atoms in GlcA, between Na^+^ and partially-charged N-acetyl N or O atoms and/or hydroxyl O atoms in GlcNAc, or between Cl^−^ and H atoms of methyl and/or hydroxyl groups in GlcNAc). Specifically, we looked for ions within 5 Å of the exocyclic atoms of the monosaccharide of interest and found that ionic interactions were most often absent during, and thus, did not significantly contribute to, ring puckering in the MD simulation of hyaluronan 20-mer.

**Figure 5:**
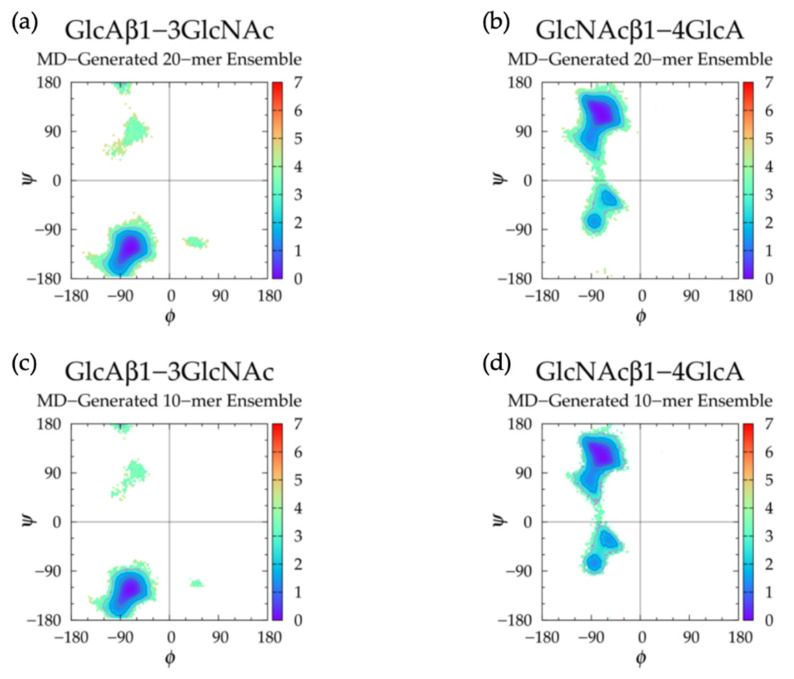
Δ*G*(*ϕ*, *ψ*) in the MD-generated hyaluronan ensembles for aggregated GlcAβ1-3GlcNAc and GlcNAcβ1-4GlcA glycosidic linkage data in the (**a**,**b**) 20-mer and (**c**,**d**) 10-mer, respectively; contour lines every 1 kcal/mol.

To analyze glycosidic linkage conformations, free energies of glycosidic linkage dihedrals Δ*G*(*ϕ*, *ψ*) were calculated (Figure 5a,b and Table 2). If we assume O_5_-C_1_-O-C*_n_* = H_1_-C_1_-O-C*_n_* - 120° for *ϕ* values and C_1_-O-C_3_-C_2_ = C_1_-O-C_3_-H_3_ - 120° and C1-O-C_4_-C_3_ = C_1_-O-C_4_-H_4_ + 120° for *ψ* values, our data match well with X-ray diffraction and force field data from hyaluronan tetrasaccharides [49], as well as NMR and molecular mechanics data from hyaluronan tetrasaccharides [111], hexasaccharides [112], and octasaccharides [113]. If we assume C_1_-O-C_3_-C_2_ = C_1_-O-C_3_-C_4_ + 120° and C_1_-O-C_4_-C_3_ = C_1_-O-C_4_-C_5_ - 120°, our data are also in close agreement with X-ray diffraction and quantum molecular modeling data [65,114,115,116], as well as NMR and MD data [102]. This agreement is with conformations sampled about the global minimum in each linkage in our MD simulations. All of these conformations were helical, so it stands to reason that 2-, 3-, and 8-fold helices were observed in hyaluronan oligosaccharides. This does not necessarily mean that adjacent glycosidic linkage conformations were interdependent. In fact, variation in the types of helices observed in the force field and experimental studies in only short oligosaccharide units suggests a lack of consistent and uniform helical structures in long hyaluronan polymers.

Our data also show that a secondary basin was sampled in each linkage type in our MD simulations. In GlcAβ1-3GlcNAc linkages, conformations in the upper left quadrant (−*ϕ*, +*ψ*) were sampled. A molecular modeling study that reportedly sampled the full allowable range of glycosidic linkage conformations in agreement with experimental data for several disaccharide units also revealed hyaluronan GlcAβ1-3GlcNAc linkage conformations in a secondary basin similar to ours [117]. Furthermore, crystal structures of hyaluronan oligosaccharides in complexes with proteins [92,93,94,95] were examined and we found that most glycosidic linkage conformations were similar to the most energetically-favorable conformations observed in MD simulation, with the exception of one GlcAβ1-3GlcNAc linkage conformation with −*ϕ*, +*ψ* dihedrals, which was seen in a hyaluronan hexasaccharide in complex with hyaluronidase [94]. This suggests that, although rare, this is a physical conformation. Analysis of individual glycosidic linkages in individual runs (Figure S7) showed that this basin was sampled mostly in two particular GlcAβ1-3GlcNAc linkages in the MD run with the lowest end-to-end distance distribution. Therefore, 20-mer MD snapshots with these glycosidic linkage conformations were analyzed; we found that these linkage conformations were associated with lower end-to-end distances (Figure S8 and Table S1). Visual analysis of these snapshots showed that GlcAβ1-3GlcNAc glycosidic linkages with −*ϕ*, +*ψ* dihedrals caused a kink in the polymer, which explains the more compact polymer conformations in comparison to those containing only linkage conformations at the global free energy minimum (Figure S8). To find out if there was a connection between these glycosidic linkage conformations and non-^4^C_1_ puckers of adjacent monosaccharide rings, glycosidic linkages flanking non-^4^C_1_ ring puckers were plotted, and they were all centered about the global minimum, i.e., −*ϕ*, −*ψ* (Figure S9). In fact, there did not appear to be any correlation between GlcAβ1-3GlcNAc linkage −*ϕ*, +*ψ* conformations and a non-^4^C_1_ ring pucker in any region of the polymer.

Analysis of GlcNAcβ1-4GlcA glycosidic linkage conformations (Figure 5b and Table 2) revealed secondary minima in the lower left quadrant (−*ϕ*, −*ψ*), which was also seen in a molecular modeling study of GAG disaccharides with results validated by experimental data [117]. In hyaluronan 20-mer MD simulations, these conformations were sampled more in the GlcNAcβ1-4GlcA linkages neighboring the GlcAβ1-3GlcNAc linkages with −*ϕ*, +*ψ* dihedrals in the MD run with the lowest end-to-end distances. The same analyses to those performed on 20-mer conformations with GlcAβ1-3GlcNAc linkages with −*ϕ*, +*ψ* dihedrals were performed on MD-generated 20-mer snapshots with GlcNAcβ1-4GlcA linkages with −*ϕ*, −*ψ* dihedrals. Similarly, these glycosidic linkage conformations caused a kink in the polymer chain and were associated with lower end-to-end distances (Figure S10 and Table S1). To determine if there was a correlation between GlcAβ1-3GlcNAc −*ϕ*, +*ψ* linkage dihedrals and GlcNAcβ1-4GlcA −*ϕ*, −*ψ* linkage dihedrals, snapshots with both of these linkage conformations flanking the same monosaccharide ring were examined. It was found that there were very few snapshots in which this was the case. GlcNAcβ1-4GlcA linkage conformations flanking non-^4^C_1_ monosaccharide rings were also analyzed, and these conformations were centered about the global minimum, i.e., −*ϕ*, +*ψ* (Figure S9). Additionally, ionic interactions (e.g., between Na^+^, O^−^ in GlcA carboxylate group, and O in C=O of adjacent GlcNAc N-acetyl group, between Cl^−^, H in GlcNAc methyl group, and H in GlcA hydroxyl groups, or between Na^+^ and O atoms or Cl^−^ and H atoms of hydroxyls in adjacent monosaccharides) did not appear to play a role in the transition of glycosidic linkage conformations between their primary and secondary *ϕ*, *ψ* basins.

Hyaluronan 10-mers were also simulated and showed the same conformations as the 20-mer in MD simulation except for GlcNAc monosaccharide rings, which sampled only ^4^C_1_ chair conformations in the 10-mer (Figure 3c,d and Figure 5c,d and Table 2). This was expected, as these conformations occurred very few times in 20-mer MD simulations, and there were half as many samples (i.e., monosaccharide rings) in the 10-mer.

Together, these results suggest that (1) realistic helical glycosidic linkage conformations in hyaluronan 10- and 20-mers are seen in MD simulation, (2) non-^4^C_1_ ring puckers, −*ϕ*, +*ψ* dihedrals in GlcAβ1-3GlcNAc linkages, and −*ϕ*, −*ψ* dihedrals in GlcNAcβ1-4GlcA linkages all contribute to more compact conformations, and (3) monosaccharide rings and glycosidic linkages in hyaluronan 10- and 20-mers behave randomly and independently in MD simulation.

#### 3.1.2. Construction Algorithm

**Figure 6:**
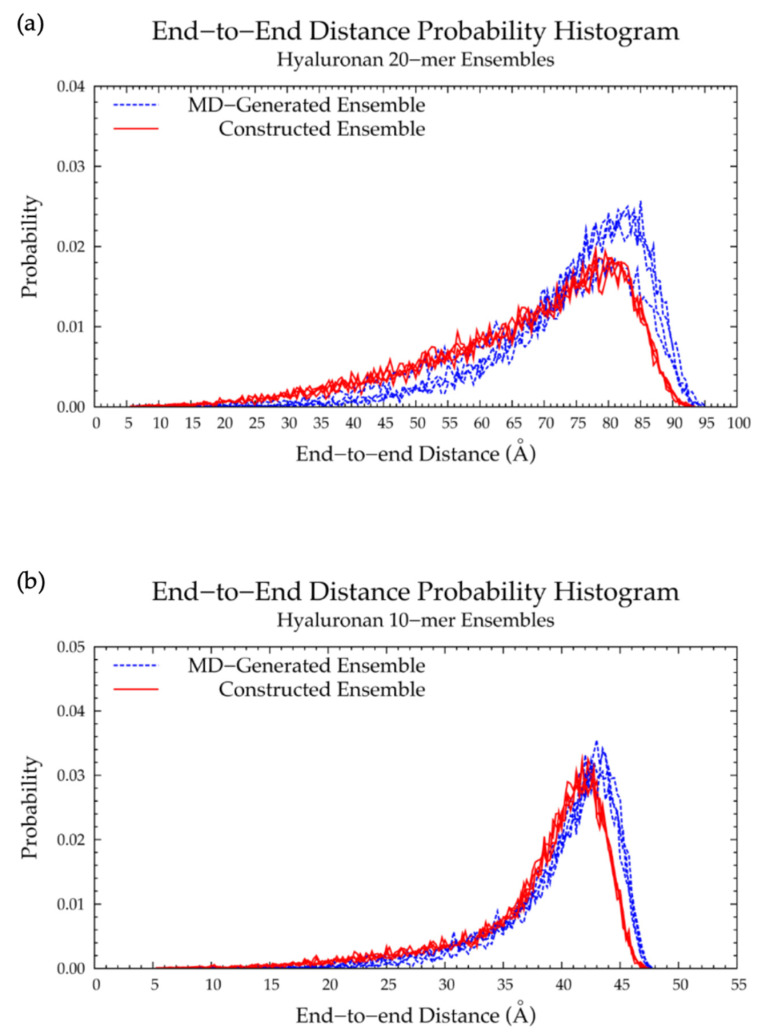
End-to-end distance probability distributions of MD-generated (blue dashed lines) and constructed (red solid lines) hyaluronan ensembles: (**a**) 20-mer and (**b**) 10-mer; probabilities were calculated for end-to-end distances sorted into 0.5 Å bins for the 20-mer and 0.25 Å bins for the 10-mer; each ensemble includes four sets of 10,000 conformations.

Our construction algorithm was used to construct four sets of hyaluronan 20-mer ensembles with 10,000 conformations each (40,000 conformations total). A comparison of end-to-end distance distributions (Figure 6a and Table 1) and radii of gyration (Figure S2a,b) showed that our construction algorithm produces ensembles that mimic backbone flexibility observed in MD simulations. Furthermore, the C-P parameters (Figure S11) and glycosidic linkage dihedral free energies Δ*G*(*ϕ*, *ψ*) (Figure S12) of constructed conformations post-minimization matched those of MD-generated ensembles (i.e., construction algorithm input data; Figure 3a,b and Figure 5a,b and Table 2), indicating that dihedral angles were properly restrained and that minimization did not change the overall ring and linkage conformation.

A hyaluronan 10-mer ensemble with 40,000 conformations was also constructed using the algorithm; similarly, the end-to-end distances and radii of gyration of the constructed 10-mer ensemble matched those of the MD-generated 10-mer ensemble (Figure 6b and Figure S2c,d and Table 1). Both ensembles contained mostly extended conformations, as expected for short oligosaccharides with fewer opportunities for kinks or curves.

**Figure 7:**
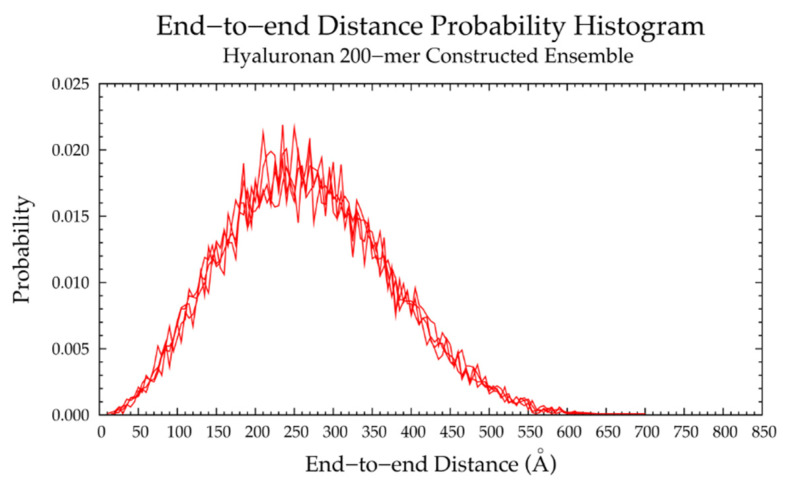
End-to-end distance probability distribution of constructed ensemble of hyaluronan 200-mer; most probable end-to-end distance across all four sets is 235 Å; probabilities were calculated for end-to-end distances sorted into 5 Å bins; the ensemble contains four sets of 10,000 conformations.

Finally, a hyaluronan 200-mer ensemble containing four sets of 10,000 conformations was constructed using the algorithm. The construction procedure produced end-to-end distances, radii of gyration, and bond potential energies of all conformations, as well as PDB files of conformations with most probable end-to-end distances, bond potential energies above that of the fully-extended conformation, and all excluded conformations (for a total of about 1000 PDBs for each of the four sets) for visualization and validation of the bond potential energy cutoff. As seen in MD-generated hyaluronan 10- and 20-mer ensembles, we expected the end-to-end distance distribution curve skewness to shift toward the right (i.e., lower end-to-end distances indicating more compact conformations) with increasing polymer length, as there were more opportunities for kinks and curves resulting from ring puckering and the flexibility of the glycosidic linkages. This pattern was seen in the end-to-end distance distributions of the hyaluronan 200-mer constructed ensembles (Figure 7) and in those of nonsulfated chondroitin 100- and 200-mer constructed ensembles [33]. The relationship between end-to-end distance and radius of gyration was decreasingly linear with increasing polymer length. In other words, conformations with the same end-to-end distance have a wider range of radii of gyration in longer polymers. This was evidenced by comparison of R^2^ values of the end-to-end distance vs. radius of gyration regression lines of hyaluronan 20- and 10-mer ensembles, both MD-generated and constructed (Figure S2a–d). As expected, the end-to-end distance and radius of gyration relationship was decreasingly linear with increasing polymer length, as shown by comparison of hyaluronan 10-, 20-, and 200-mer constructed ensembles (Figure S2b,d,e). This suggests that our algorithm constructs hyaluronan 200-mer ensembles with the backbone conformations that we would expect to see in MD simulations.

### 3.2. Nonsulfated Dermatan

#### 3.2.1. Molecular Dynamics Simulations: Glycosidic Linkage and Monosaccharide Ring Geometry Effects on Polymer Backbone Flexibility

**Figure 8:**
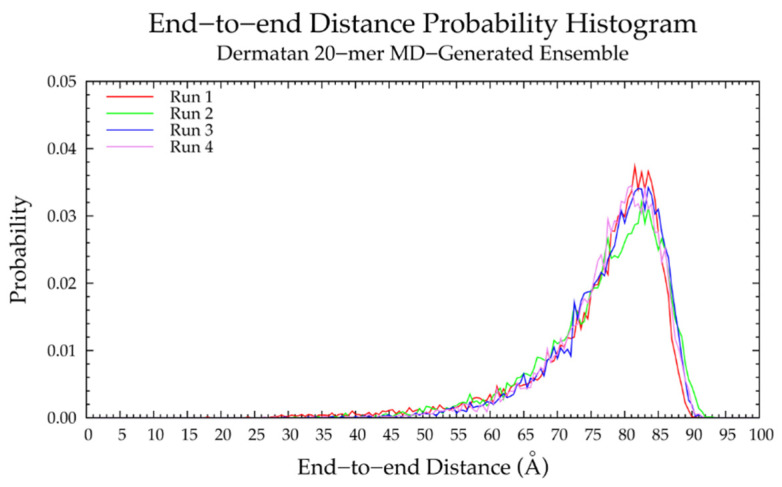
End-to-end distance probability distribution of MD-generated nonsulfated dermatan 20-mer ensemble; each of the four runs includes 10,000 conformations; probabilities were calculated for end-to-end distances sorted into 0.5 Å bins.

MD simulations of nonsulfated dermatan 20-mer revealed relatively rigid and linear backbone conformations, as evidenced by the narrow and highly left-skewed end-to-end distance distributions, which match in all four MD runs (Figure 8 and Table 3), and the linear relationship between end-to-end distance and radius of gyration (Figure S13a). To understand the factors contributing to this rigid behavior of the dermatan 20-mer in simulation, conformations of monosaccharide rings and glycosidic linkages were analyzed.

**Figure 9:**
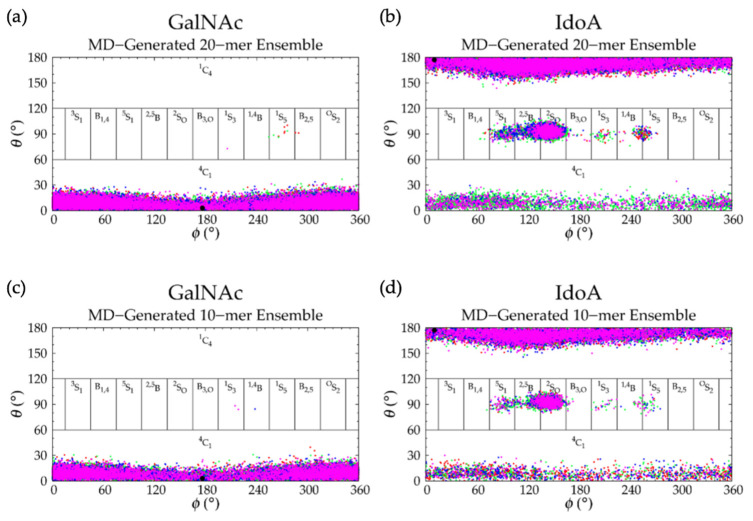
Cremer–Pople data for MD-generated nonsulfated dermatan ensembles: (**a**) GalNAc and (**b**) IdoA in the 20-mer and (**c**) GalNAc and (**d**) IdoA in the 10-mer; geometries from the four sets of each ensemble are represented by red, green, blue, and magenta dots, respectively and the force-field geometry is represented by a single large black dot; Cremer–Pople parameters (*ϕ*, *θ*) for all rings in every tenth snapshot from each ensemble were plotted (i.e., 10 rings × 1000 snapshots per run × 4 runs = 40,000 parameter sets for the 20-mer and 20,000 parameter sets for the 10-mer).

GalNAc monosaccharide ring C-P parameters (Figure 9a) show mostly ^4^C_1_ conformations, as well-established by X-ray diffraction [49,106,118], NMR [47,119], and force field [33,47,49,107] data. Biased MD simulations of β-GalNAc monosaccharides showed that nonsulfated β-GalNAc sampled boat and skew-boat conformations, namely B_3,O_, ^1^S_3_, and ^1,4^B, with relatively high free energies [107]. In line with this study, our simulations showed very few boat and skew-boat puckers, namely ^1^S_3_, ^1,4^B, ^1^S_5_ (Figure 4a; -3GalNAcβ1- endocyclic ring and linker oxygen atoms were identical to those of -3GlcNAcβ1-), and B_2,5_, on the microsecond timescale. These puckers were sampled by different rings of the 20-mer in different MD runs and returned to ^4^C_1_ within 10 ns (Figure S14), suggesting that this behavior was random, and confirming that non-^4^C_1_ GalNAc puckers are not energetically favorable. While these boat and skew-boat conformations caused a kink in the polymer chain, there were so few occurrences of this that they did not impact the overall end-to-end distance distribution.

**Figure 10:**
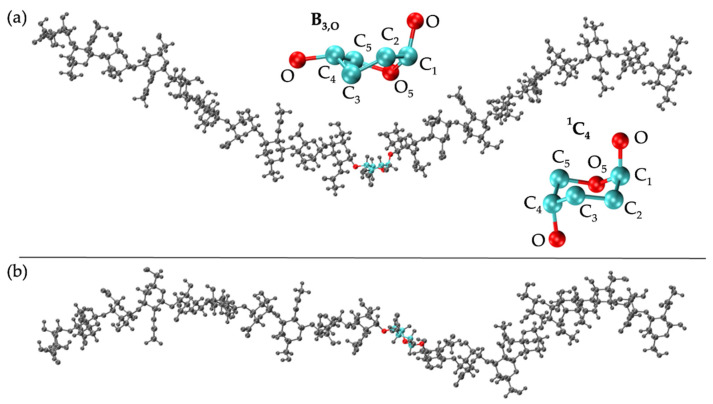
MD-generated nonsulfated dermatan conformations: (**a**) 20-mer with a B_3,O_ IdoA conformer (ring atoms in cyan; linkage atoms in red) and B_3,O_ IdoA and ^1^C_4_ IdoA monosaccharide conformations (endocyclic ring atoms and linker oxygen atoms only); (**b**) 20-mer with a ^2^S_O_ IdoA conformer highlighted (ring atoms in cyan; linkage atoms in red; ^2^S_O_ α-IdoA is identical to ^2^S_O_ β-GlcA in Figure 4c); in both 20-mer conformations, all IdoA monosaccharides in gray are in ^1^C_4_ and all GalNAc monosaccharides are in ^4^C_1_.

IdoA monosaccharide rings in 20-mer MD sampled a majority of ^1^C_4_ and, to a lesser degree, ^2^S_O_ and ^4^C_1_ puckers (Figure 9b and Table S2), as observed in NMR and force field studies [29,45,47,51,107,120,121,122,123,124,125]. Literature reports of the relative proportions of these three ring puckers in nonsulfated IdoA vary, which may be explained by differences in the structure of neighboring residues (or lack thereof) [45,120,123,124] and differences in ion concentrations [123]. Furthermore, existing studies were conducted on IdoA monosaccharides and short IdoA-containing GAG oligosaccharides (i.e., <20 monosaccharides). NMR data have shown that, when at the nonreducing terminal in GAG oligosaccharides, nonsulfated IdoA is in equilibrium with ^1^C_4_, ^2^S_O_, and ^4^C_1_ puckers, and when flanked by nonsulfated N-acetylated sugar derivatives (i.e., GalNAc or GlcNAc), nonsulfated IdoA is primarily in ^1^C_4_ and ^2^S_O_ conformations [123], with fewer than 10% of samples in ^4^C_1_ [45,124]. Indeed, we found this to be the case in our dermatan 20-mer MD simulations (Table S2). Importantly, none of the ^1^C_4_, ^2^S_O_, or ^4^C_1_ puckers introduced a kink in the polymer chain, so we would not expect variations in the relative proportions of these ring puckers to alter overall polymer backbone conformations. IdoA also sampled other boat and skew-boat conformations in 20-mer MD simulations, specifically B_1,4_, ^5^S_1_, ^2,5^B, B_3,O_, ^1^S_3_, ^1,4^B, and ^1^S_5_ (Figure 9b and Table S2), which is in agreement with results from MD simulations of IdoA monosaccharides [107,126]. Another force field study showed that ^2,5^B and B_3,O_ IdoA conformers are also feasible interpretations of existing NMR data [127]. Furthermore, a force field study that mapped X-ray diffraction and NMR data for dermatan sulfate [122] and a molecular modeling study of IdoA monosaccharides [126] showed that interconversion between different boat/skew-boat forms of IdoA is much more common than that between boat/skew-boat and chair forms, as it is less energetically costly, which helps explain the sampling of multiple different boat/skew-boat IdoA conformations. As each of these boat/skew-boat puckers (except for ^2^S_O_) introduces a kink in the polymer chain (Figure 10 and Figure 4b,c; -4IdoAα1- endocyclic ring and linker oxygen atoms are identical to those of -4GlcAcβ1-), end-to-end distances of 20-mer conformations with these puckers were analyzed to determine if they were associated with more compact conformations. The end-to-end distance distribution of conformations with boat/skew-boat ring puckers (other than ^2^S_O_) matched that of the average of the four runs from the full MD-generated 20-mer ensemble (Figure S15), indicating that these ring puckers do not necessarily give compact polymer conformations. Additionally, the end-to-end distance distribution of 20-mer conformations with ^2^S_O_ IdoA conformations was compared to that of the full MD ensemble (Figure S15). These distributions were similar, further suggesting that ^2^S_O_ conformations are not associated with more or less compact 20-mer conformations. To determine if IdoA ring puckering in nonsulfated dermatan 20-mer MD was random, an analysis was performed of C-P parameters of individual IdoA monosaccharides in each of the four MD runs (Figure S16). This revealed that (1) not all IdoA rings overcame the energy barrier to sample a ^4^C_1_ pucker, (2) no single IdoA ring sampled ^4^C_1_ in all four MD runs, (3) each IdoA ring overcame the energy barrier to sample boat/skew-boat puckers (i.e., C-P *θ* ~ 90°) in at least one MD run, and (4) after sampling boat/skew-boat puckers, some IdoA rings returned to ^1^C_4_ in as little as 165 ns, while others remained in boat/skew-boat conformations for the remainder of the 1-µs trajectory. These observations support the idea that monosaccharide rings in a nonsulfated dermatan 20-mer behave randomly and independently in unbiased MD simulation.

**Figure 11:**
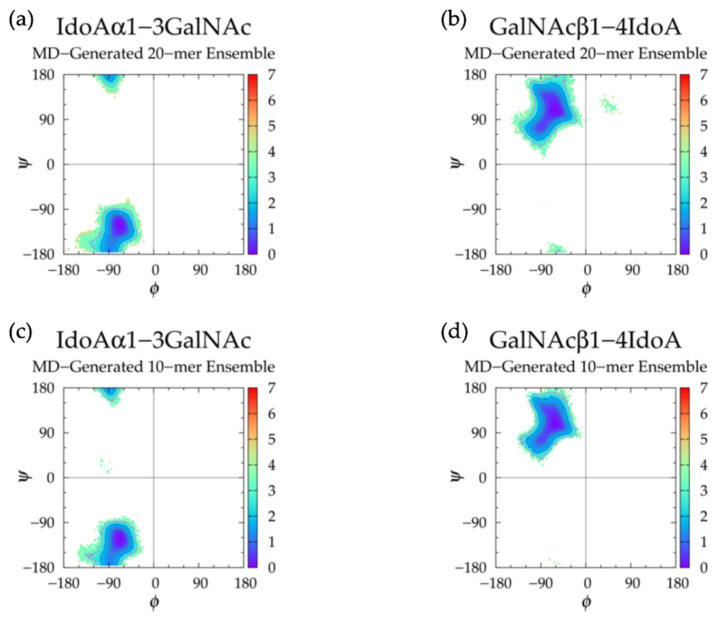
Δ*G*(*ϕ*, *ψ*) in the MD-generated nonsulfated dermatan ensembles for aggregated IdoAα1-3GalNAc and GalNAcβ1-4IdoA glycosidic linkage data in the (**a**,**b**) 20-mer and (**c**,**d**) 10-mer, respectively; contour lines every 1 kcal/mol.

Analysis of dihedral free energies revealed a single Δ*G*(*ϕ*, *ψ*) minimum for each of IdoAα1-3GalNAc and GalNAcβ1-4IdoA glycosidic linkages (Figure 11a,b and Table 4). This is consistent across all linkages in all runs for each linkage type. The lack of secondary basins in *ϕ*, *ψ* dihedral samples may explain the higher degree of rigidity and tendency toward extended backbone conformations of the nonsulfated dermatan 20-mer in comparison to hyaluronan and nonsulfated chondroitin [33] 20-mers in MD simulation. An X-ray diffraction study of sodium dermatan sulfate observed three different helical forms (i.e., right-handed, left-handed, and achiral) [118] and quantified their dihedral angles, which were defined by: *ϕ* = O_5_-C_1_-O-C*_n_* and *ψ* = C_1_-O-C*_n_*-C_(*n* + 1)_. Assuming C_1_-O-C_3_-C_2_ = C_1_-O-C_3_-C_4_ + 120° and C1-O-C_4_-C_3_ = C_1_-O-C_4_-C_5_ - 120°, we see that the right-handed and achiral helical forms corresponded to high Δ*G*(*ϕ*, *ψ*) values in our nonsulfated dermatan 20-mer MD simulations, and the left-handed helical form corresponded to glycosidic linkage conformations very close to the Δ*G*(*ϕ*, *ψ*) global minimum. In line with our results, force field studies that compared MD results to existing NMR and X-ray diffraction data of dermatan sulfate dismissed right-handed and achiral helical forms and confirmed the presence of left-handed helical structure [49,122]. A quantitative comparison of our MD-generated glycosidic linkage data to their findings and other NMR data for dermatan tetrasaccharides [47] showed close agreement if we assume O_5_-C_1_-O-C*_n_* = H_1_-C_1_-O-C*_n_* - 120° for all *ϕ* values, C_1_-O-C_3_-C_2_ = C_1_-O-C_3_-H_3_ - 120° for α1-3 linkage *ψ* values, and C1-O-C_4_-C_3_ = C_1_-O-C_4_-H_4_ + 120° for β1-4 linkage *ψ* values.

MD simulations of a nonsulfated dermatan 10-mer produced mostly linear backbone conformations (Figure S13c) and similar monosaccharide ring (Figure 9) and glycosidic linkage (Figure 11 and Table 4) geometries to the nonsulfated dermatan 20-mer. The major difference was that in 10-mer MD simulations, 9% of all IdoA rings in all runs were found in ^4^C_1_ form; this was almost exclusively in the nonreducing terminal IdoA (i.e., ring #10; Figure S17 and Table S2), which is mostly in line with the aforementioned NMR study of IdoA ring conformations [45]. However, based on the findings from the NMR study and from our dermatan 20-mer MD simulations, we would still expect the nonterminal IdoA rings in a GAG polymer to sample some ^4^C_1_ conformations. This supports our position that GAG 20-mers are better-suited for predictions of long-chain backbone conformations than short GAG oligosaccharides.

These findings suggest that (1) monosaccharide rings and glycosidic linkages in nonsulfated dermatan GAG 20-mers behave randomly and independently in MD simulation, (2) nonsulfated dermatan polymers take on rigid left-handed helical structure with a tendency toward linear backbone conformations in unbiased MD simulations, and (3) nonsulfated dermatan 20-mer conformations in MD simulation provide a realistic representation of longer nonsulfated dermatan polymer conformations.

#### 3.2.2. Construction Algorithm

**Figure 12:**
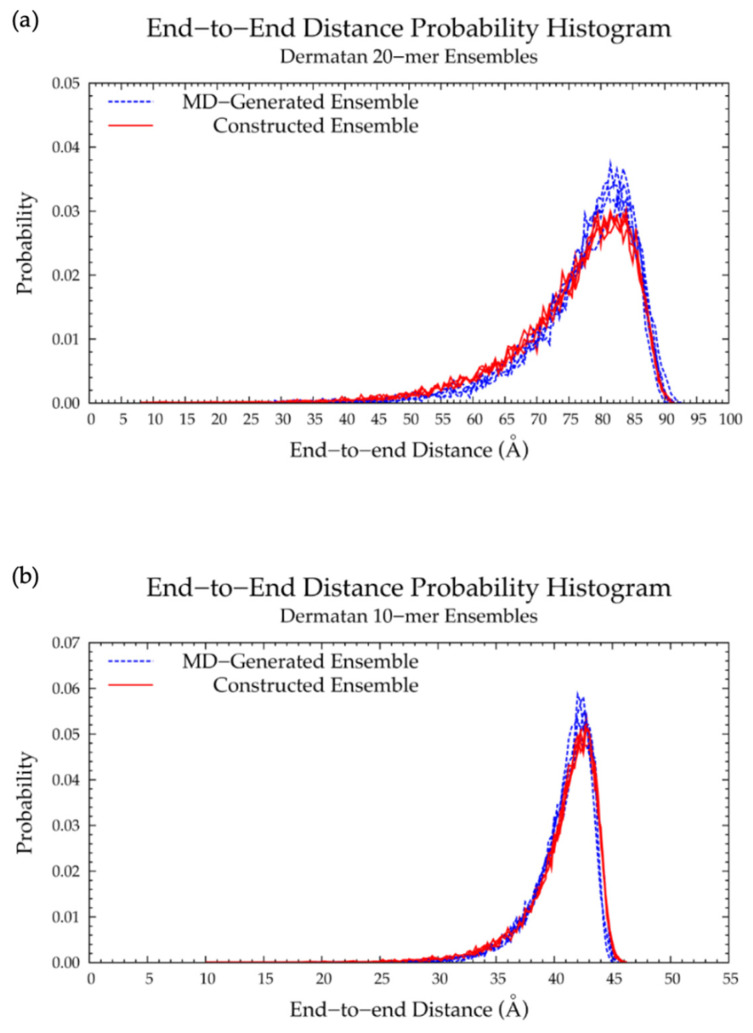
End-to-end distance probability distributions of MD-generated (blue dashed lines) and constructed (red solid lines) nonsulfated dermatan ensembles: (**a**) 20-mer and (**b**) 10-mer; probabilities were calculated for end-to-end distances sorted into 0.5 Å bins for the 20-mer and 0.25 Å bins for the 10-mer; each ensemble includes four sets of 10,000 conformations.

Four sets of 10,000 conformations of a nonsulfated dermatan 20-mer were constructed using our algorithm and compared to conformations seen in nonsulfated dermatan 20-mer MD. As expected, the C-P parameter plots (Figure S18) and glycosidic linkage free energies (Figure S19) in the constructed 20-mer ensemble matched those in the MD-generated 20-mer ensemble (Figure 9a,b and Figure 11a,b and Table 4). Furthermore, the end-to-end distances (Figure 12a) and radii of gyration (Figure S13a,b) in the constructed ensemble matched those in the MD-generated ensemble, with only a 2.42% difference in most probable end-to-end distance (Table 3). This demonstrates that our algorithm produces nonsulfated dermatan 20-mer ensembles that mimic 20-mer backbone flexibility seen in MD simulations.

**Figure 13:**
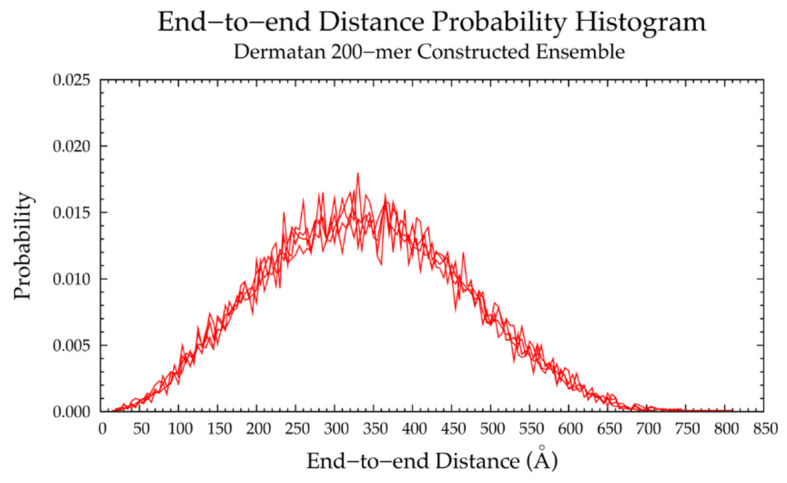
End-to-end distance probability distribution of constructed ensemble of nonsulfated dermatan200-mer; most probable end-to-end distance across all four sets is 365 Å; probabilities were calculated for end-to-end distances sorted into 5 Å bins; the ensemble contains four sets of 10,000 conformations.

The nonsulfated dermatan 10-mer ensemble with 40,000 conformations constructed by the algorithm had very similar end-to-end distance and radius of gyration data compared to the MD-generated ensemble (Figure 12b and Figure S13c,d and Table 3). The algorithm was also used to create a nonsulfated dermatan 200-mer ensemble. As expected, the 200-mer end-to-end distance distribution (Figure 13) and radii of gyration (Figure S13e) showed that conformations tended to be more compact as polymer length increased. The shift in skewness of the end-to-end distance distribution with increasing polymer length was more subtle in nonsulfated dermatan than in hyaluronan, as there were fewer linkage and ring conformations causing kinks in the polymer.

### 3.3. Nonsulfated Keratan

#### 3.3.1. Molecular Dynamics Simulations: Glycosidic Linkage and Monosaccharide Ring Geometry Effects on Polymer Backbone Flexibility

**Figure 14:**
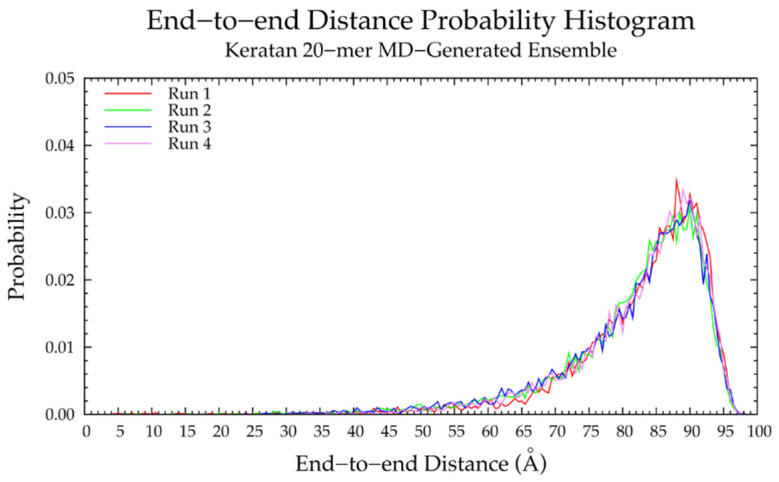
End-to-end distance probability distribution of MD-generated nonsulfated keratan 20-mer ensemble; each of the four runs includes 10,000 conformations; probabilities were calculated for end-to-end distances sorted into 0.5 Å bins.

In MD simulations, nonsulfated keratan 20-mers showed rigid backbone behavior and favored extended backbone conformations, as evidenced by end-to-end distance distributions (Figure 14 and Table 5) which were identical in all four MD runs, and the high correlation of end-to-end distance to radius of gyration (Figure S20a). To understand the causes of this rigid behavior, monosaccharide ring and glycosidic linkage conformations were analyzed.

**Figure 15:**
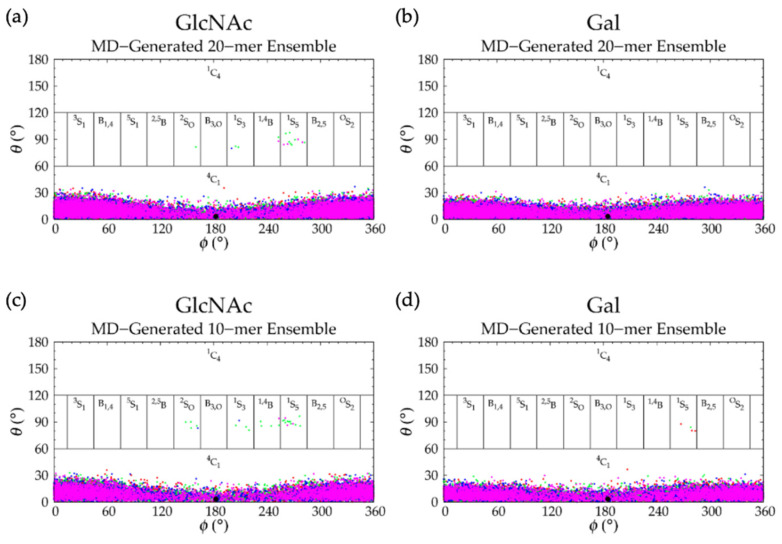
Cremer–Pople data for MD-generated nonsulfated keratan ensembles: (**a**) GlcNAc and (**b**) Gal in the 20-mer and (**c**) GlcNAc and (**d**) Gal in the 10-mer; geometries from the four sets of each ensemble are represented by red, green, blue, and magenta dots, respectively and the force-field geometry is represented by a single large black dot; Cremer–Pople parameters (*ϕ*, *θ*) for all rings in every tenth snapshot from each ensemble were plotted (i.e., 10 rings × 1000 snapshots per run × 4 runs = 40,000 parameter sets for the 20-mer and 20,000 parameter sets for the 10-mer).

C-P parameters of GlcNAc monosaccharides in keratan 20-mer MD (Figure 15a) showed similar conformations to GlcNAc in hyaluronan 20-mer. There were predominantly ^4^C_1_ chair conformations, which are also found in a keratan sulfate tetrasaccharide crystal structure [128], with very few transitions (i.e., no more than one per ring or two per run) to boat/skew-boat conformations (i.e., ^2^S_O_, ^1^S_3_, ^1,4^B, and ^1^S_5_), which were sampled for no more than 20 ns before returning to ^4^C_1_ chair (Figure S21). Analysis of end-to-end distance and radius of gyration of 20-mer conformations containing non-^4^C_1_ ring puckers showed that these ring puckers were not associated with compact 20-mer conformations (Figure S22). C-P analysis of Gal monosaccharides showed that for the duration of the keratan 20-mer MD simulations, Gal remained in ^4^C_1_ chair (Figure 15b), which was the predominant conformation found in NMR [119,129,130,131] and force field data [107] for Gal in general, and in a keratan sulfate tetrasaccharide crystal structure [128].

**Figure 16:**
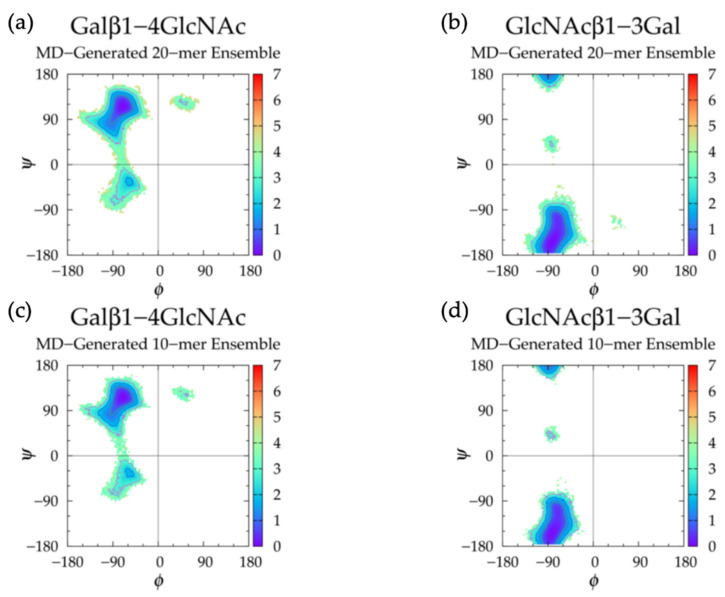
Δ*G*(*ϕ*, *ψ*) in the MD-generated nonsulfated keratan ensembles for aggregated Galβ1-4GlcNAc and GlcNAcβ1-3Gal glycosidic linkage data in the (**a**,**b**) 20-mer and (**c**,**d**) 10-mer, respectively; contour lines every 1 kcal/mol.

Glycosidic linkage free energy Δ*G*(*ϕ*, *ψ*) analysis of Galβ1-4GlcNAc linkages revealed a global minimum in the basin with −*ϕ*, +*ψ* and a secondary minimum in the basin with −*ϕ*, −*ψ* (Figure 16a and Table 6), similar to hyaluronan GlcNAcβ1-4GlcA linkages (Figure 5b,d and Table 2), but with additional rare conformations in +*ϕ*, +*ψ*. A molecular modeling study, which was shown to agree with experimental data, showed that β1-4 linkages in keratan sulfate disaccharides took on the same conformations as β1-4 linkages in hyaluronan disaccharides [117], confirming the primary and secondary basins sampled in our 20-mer MD simulations. Keratan 20-mer conformations with glycosidic linkage conformations in the tertiary basin (i.e., +*ϕ*, +*ψ*) were visualized and end-to-end distances were analyzed (Figure S23). These conformations formed a slight bend in the polymer chain and were associated with more compact polymer backbone conformations. However, there did not appear to be any close contacts between atoms of adjacent monosaccharides in this conformation or in previous snapshots that could explain these linkage bond rotations. Furthermore, this conformation was not specific to any particular region of the polymer. Therefore, this behavior was likely random. As −*ϕ*, −*ψ* β1-4 linkage conformations are associated with compact hyaluronan polymer conformations, and nonsulfated keratan 20-mer is relatively rigid and favors extended conformations in contrast to hyaluronan, we sought to determine the effects of Galβ1-4GlcNAc linkage conformations in the secondary (i.e., −*ϕ*, −*ψ*) basin on keratan 20-mer backbone flexibility in MD simulations. As in hyaluronan, these conformations caused a kink in the keratan 20-mer chain and were associated with more compact conformations (Figure S23 and Table S1). However, these conformations were slightly less stable in keratan 20-mer MD (Table 6) than in hyaluronan MD (Table 2), and were, therefore, less common.

Analysis of GlcNAcβ1-3Gal glycosidic linkages showed a global free energy minimum in the −*ϕ*, −*ψ* basin (Table 6) similar to that of IdoAβ1-3GalNAc linkages in nonsulfated dermatan MD (Figure 11a,c and Table 4), but with additional rare occurrences in a secondary basin in −*ϕ*, +*ψ*, confirmed by the aforementioned molecular modeling study [117], and a small tertiary basin in +*ϕ*, −*ψ* (Figure 16b). To determine the effects of GlcNAcβ1-3Gal linkage conformations in these secondary and tertiary basins on backbone conformation, we visualized 20-mer conformations containing these glycosidic linkage conformations and analyzed end-to-end distances (Figure S24). Glycosidic linkage conformations in the tertiary basin caused a kink and were associated with more compact 20-mer conformations. In contrast to hyaluronan, nonsulfated keratan β1-3 glycosidic linkage conformations in the secondary basin (i.e., −*ϕ*, +*ψ*) caused only a slight bend in the polymer chain, but were still associated with compact 20-mer conformations (Table S1). Both secondary and tertiary GlcNAcβ1-3Gal linkage conformations were rare in keratan 20-mer MD, which helps explain why the keratan 20-mer chain was relatively rigid and favored extended conformations in MD simulation.

Backbone conformational analysis of nonsulfated keratan 10-mer MD revealed that the 10-mer chain was rigid and favored extended conformations (Figure S20c and Table 5), which is similar behavior to that of nonsulfated keratan 20-mer in MD simulation. This stands to reason, as conformations of monosaccharide rings (Figure 15) and glycosidic linkages (Figure 16 and Table 6) in keratan 10-mer MD matched those in keratan 20-mer MD, with the only differences being that (1) Gal briefly (i.e., for < 5 ns) sampled a ^1^S_5_ skew-boat conformation once in each of two of the 10-mer MD runs (Figure S25), and (2) GlcNAcβ1-3Gal linkages in the 10-mer did not sample conformations in the tertiary basin (i.e., +*ϕ*, −*ψ*) sampled in the 20-mer. Boat/skew-boat conformations including ^1^S_5_ were shown to have a high secondary free energy minimum (i.e., > 4 kcal/mol) in biased MD simulations of Gal monosaccharides [107], indicating that while possible, these conformations are not very stable in Gal monosaccharides. In both biased and unbiased MD simulations of a tetrasaccharide with a central Gal-GlcNAc disaccharide unit, the free energy minimum of Gal boat/skew-boat conformations was much higher (i.e., ~7 kcal/mol) [108], suggesting that non-^4^C_1_ conformations are less stable in Gal linked to other monosaccharides. This could explain why Gal does not sample non-^4^C_1_ conformations in keratan 20-mer MD, and suggests that these conformations would not be seen in long polymers. Although +*ϕ*, −*ψ* conformations in GlcNAcβ1-3Gal linkages are rare, they are physical conformations [117] that minorly contribute to polymer backbone flexibility. This further supports our belief that conformational landscapes from 20-mer MD are better-suited to construct conformational ensembles of GAGs with biologically-relevant chain lengths than those of short GAG oligosaccharides.

The higher degree of rigidity and probability of extended conformations in nonsulfated keratan MD compared to hyaluronan MD can be explained by the facts that: (1) for each glycosidic linkage type, the conformations that caused a kink in the polymer chain were less stable and, thus, rarer in nonsulfated keratan MD than in hyaluronan MD; and (2) GlcNAc and Gal are mostly rigid ^4^C_1_ chair conformers in nonsulfated keratan whereas GlcNAc and GlcA took on more boat/skew-boat conformations that caused kinks in hyaluronan. Importantly, nonsulfated keratan 10- and 20-mers behaved randomly, and glycosidic linkages and monosaccharide rings behaved independently in MD simulations.

#### 3.3.2. Construction Algorithm

**Figure 17:**
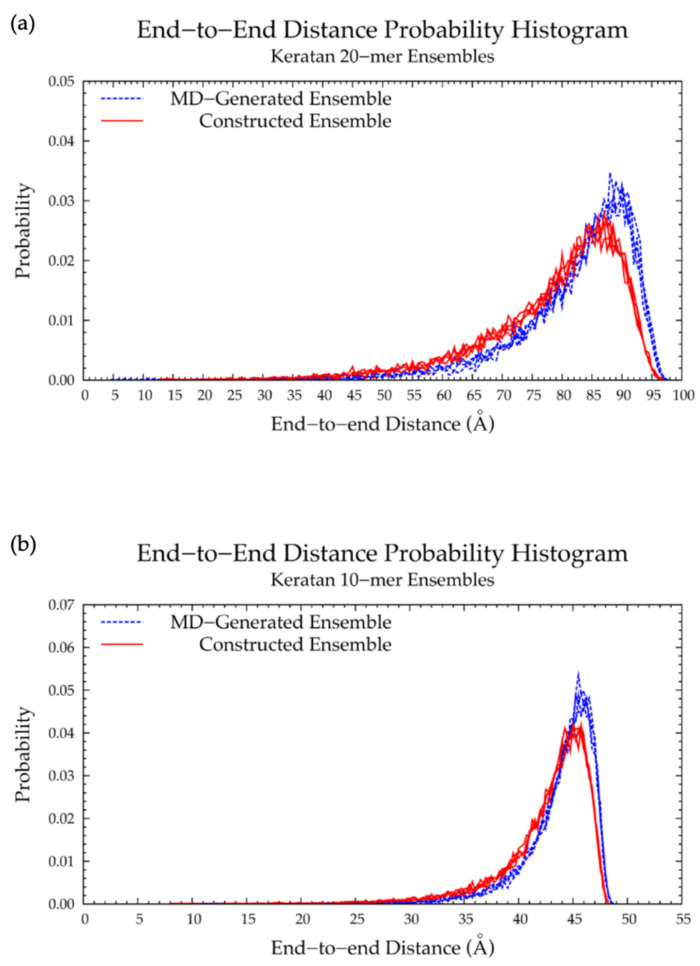
End-to-end distance probability distributions of MD-generated (blue dashed lines) and constructed (red solid lines) nonsulfated keratan ensembles: (**a**) 20-mer and (**b**) 10-mer; probabilities were calculated for end-to-end distances sorted into 0.5 Å bins for the 20-mer and 0.25 Å bins for the 10-mer; each ensemble includes four sets of 10,000 conformations.

Nonsulfated keratan 20-mer conformational ensembles (four sets of 10,000 conformations) were constructed using our algorithm and compared to MD-generated keratan 20-mer ensembles. The monosaccharide ring (Figure S26) and glycosidic linkage (Figure S27) conformations in the constructed keratan 20-mer ensemble were identical to the input data (i.e., MD-generated keratan 20-mer conformations; Figure 15a,b and Figure 16a,b and Table 6), as expected. The end-to-end distances (Figure 17a and Table 5) and radii of gyration (Figure S20a,b) were similar in constructed and MD-generated ensembles, demonstrating that nonsulfated keratan 20-mer conformational ensembles provide an accurate representation of backbone flexibility in nonsulfated keratan 20-mer MD simulations.

**Figure 18:**
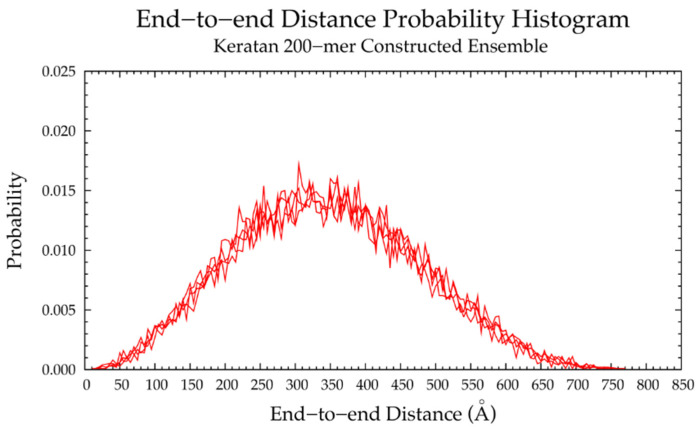
End-to-end distance probability distribution of constructed ensemble of nonsulfated keratan 200-mer; most probable end-to-end distance across all four sets is 305 Å; probabilities were calculated for end-to-end distances sorted into 5 Å bins; the ensemble contains four sets of 10,000 conformations.

Four sets of nonsulfated keratan 10-mer ensembles with 10,000 conformations each were constructed, and the results were compared to those of nonsulfated keratan 10-mer MD. The end-to-end distances (Figure 17b and Table 5) and radii of gyration (Figure S20c,d) in constructed 10-mer ensembles matched those of MD-generated 10-mer ensembles, demonstrating that MD-generated 20-mer conformations can be applied to construct ensembles of keratan polymers of different lengths that mimic backbone flexibility seen in MD simulation. A nonsulfated keratan 200-mer ensemble was constructed and, as expected, the end-to-end distance distribution (Figure 18) and radii of gyration (Figure S20e) showed that as the polymer length increased, conformations tended to be more compact. Notably, this shift in the end-to-end distance distribution curve was more subtle in keratan than in hyaluronan, which stands to reason, as monosaccharide rings and glycosidic linkages are more rigid and tend to be more extended in keratan 20-mer MD than in hyaluronan 20-mer MD.

### 3.4. Nonsulfated Heparan

#### 3.4.1. Molecular Dynamics Simulations: Glycosidic Linkage and Monosaccharide Ring Geometry Effects on Polymer Backbone Flexibility

**Figure 19:**
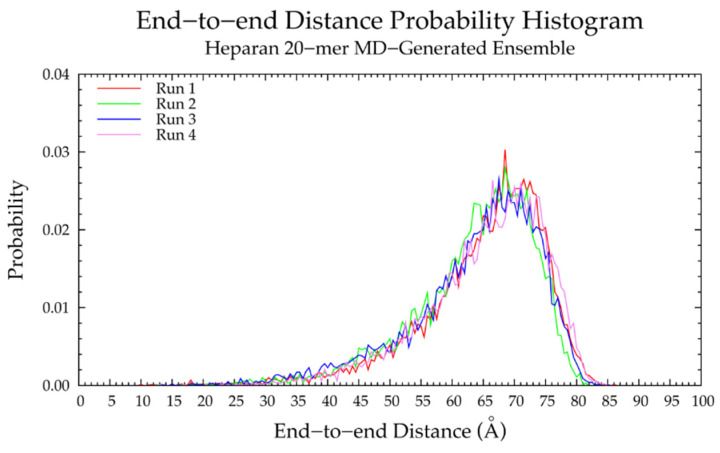
End-to-end distance probability distribution of MD-generated nonsulfated heparan 20-mer ensemble; each of the four runs includes 10,000 conformations; probabilities were calculated for end-to-end distances sorted into 0.5 Å bins.

The backbone flexibility of nonsulfated heparan 20-mer, quantified by the end-to-end distances (Figure 19 and Table 7) and radii of gyration (Figure S28a) in MD simulations was analyzed. The wide end-to-end distance distribution curves and tendency toward lower end-to-end distances, relative to those of the extended 20-mer conformation, indicated that nonsulfated heparan 20-mer was highly flexible and tended toward compact conformations in MD simulations. To determine factors contributing to this conformational flexibility, monosaccharide ring and glycosidic linkage conformations were examined for patterns.

**Figure 20:**
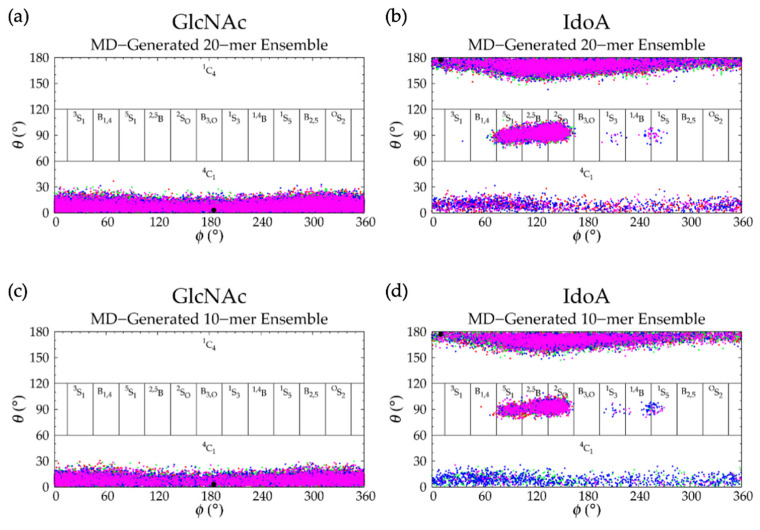
Cremer–Pople data for MD-generated nonsulfated heparan ensembles: (**a**) GlcNAc and (**b**) IdoA in the 20-mer and (**c**) GlcNAc and (**d**) IdoA in the 10-mer; geometries from the four sets of each ensemble are represented by red, green, blue, and magenta dots, respectively and the force-field geometry is represented by a single large black dot; Cremer–Pople parameters (*ϕ*, *θ*) for all rings in every tenth snapshot from each ensemble were plotted (i.e., 10 rings × 1000 snapshots per run × 4 runs = 40,000 parameter sets for the 20-mer and 20,000 parameter sets for the 10-mer).

C-P parameters of GlcNAc rings (Figure 20a) revealed all ^4^C_1_ chair conformations, in line with NMR and force field data for α-GlcNAc [119]. This stands to reason, as we would not expect much difference in ring conformation between α- and β-GlcNAc, because the only structural difference is the orientation of the exocyclic oxygen atom on C_1_ (i.e., the linker oxygen on nonterminal GlcNAc monosaccharides in GAG polysaccharides). This structural difference will impact glycosidic linkage conformation but is less likely to impact GlcNAc ring conformation. The IdoA monosaccharide C-P parameters (Figure 20b) from nonsulfated heparan 20-mer MD were similar to those of IdoA in nonsulfated dermatan 20-mer MD (Figure 9b): predominantly ^1^C_4_, ^2^S_O_, and some ^4^C_1_, which is also in line with NMR and force field data for nonsulfated IdoA in heparan sulfate oligosaccharides [120,123] and nonsulfated heparan trisaccharides [126], and occasional boat and skew-boat conformations (Figure 4b,c; -4IdoAα1- endocyclic ring and linker oxygen atoms in heparan are identical to those of -4GlcAβ1- in hyaluronan). As with GalNAc in dermatan, the presence of neighboring GlcNAc monosaccharides substantially decreased sampling of ^4^C_1_ conformations [123], which is in line with our results. One NMR and force field study reported 19–49% ^2^S_O_ conformations of nonsulfated IdoA in heparan sulfate hexasaccharides, and found that as the degree of GlcNAc sulfation increased, the percentage of ^2^S_O_ conformations in adjacent nonsulfated IdoA decreased [120]. As we studied only nonsulfated heparan, we would expect to see a higher proportion of IdoA ^2^S_O_ conformations (i.e., close to 49%) if simulated under the same conditions. However, the aforementioned study performed MD in aqueous solution with only neutralizing Na^+^ ions, whereas our systems contained an additional 140 mM NaCl. Another NMR and molecular modeling study showed that increasing NaCl salt concentrations caused a shift in equilibrium of ^1^C_4_ and ^2^S_O_ toward ^1^C_4_ conformations of 2-O-sulfated IdoA flanked by sulfated glucosamine [123]. This may explain the tendency toward ^1^C_4_ IdoA conformations, with ~54% of IdoA rings across all four 20-mer MD runs in ^1^C_4_, ~25% in ^2^S_O_, and ~4% in ^4^C_1_ (Table S3). Furthermore, very few IdoA rings sampled ^4^C_1_ chair conformations, and one run contained no IdoA ^4^C_1_ chair conformations (Figure S29), which is in contrast to the more random distribution of IdoA ^4^C_1_ chair conformations in nonsulfated dermatan 20-mer MD (Figure S16). This is likely the result of random kinetic trapping during nonsulfated heparan 20-mer MD simulations, which means IdoA only rarely overcame energy barriers between boat/skew-boat and ^4^C_1_ conformations. Importantly, it appears that all relevant IdoA conformations (i.e., those in line with the literature) were sampled in nonsulfated heparan 20-mer MD simulations. Therefore, we believe our database of MD-generated 20-mer conformations contains a full conformational landscape of IdoA rings in nonsulfated heparan.

The primary goal of our algorithm is to predict backbone conformations of long GAG chains, which requires that (1) monosaccharide rings in GAG 20-mers behave independently in MD simulation and (2) contributions of ring puckers to backbone flexibility match those expected in nonsulfated heparan polymers in aqueous solution. There did not appear to be any interdependency between adjacent rings, suggesting that monosaccharide rings behave independently in nonsulfated heparan 20-mer MD simulations. To determine the effects of IdoA ring puckering on polymer backbone conformations, end-to-end distances of 20-mer conformations containing monosaccharides in (1) boat and skew-boat conformations that caused a kink in the polymer chain and (2) ^2^S_O_ conformations, were analyzed (Figure S30). The end-to-end distance distribution of 20-mer conformations with the ring puckers that caused a kink was similar to that of the average of the four MD runs and the most probable end-to-end distance was only 1 Å lower in conformations with ring puckers that caused a kink, suggesting that boat and skew-boat conformations that introduce a kink do not necessarily give less compact 20-mer conformations, and thus are not major factors contributing to backbone flexibility. The distribution curve of 20-mer conformations with ^2^S_O_ IdoA ring puckers and that of 20-mer conformations with non-^2^S_O_ boat/skew-boat puckers that caused a kink were qualitatively similar to that of the full 20-mer MD ensemble, but had additional peaks in probability of higher end-to-end distances. These findings suggest that IdoA boat/skew-boat conformations are associated with the full range of 20-mer backbone conformations in MD simulations with only a slight tendency toward extended 20-mer conformations. Therefore, the proportion of IdoA ^2^S_O_ conformations likely does not have a major effect on nonsulfated heparan 20-mer backbone flexibility.

**Figure 21:**
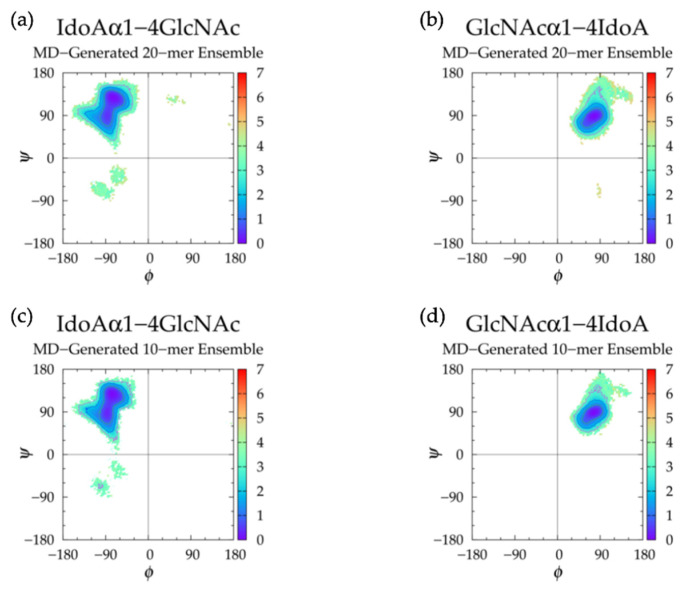
Δ*G*(*ϕ*, *ψ*) in the MD-generated nonsulfated heparan ensembles for aggregated IdoAα1-4GlcNAc and GlcNAcα1-4IdoA glycosidic linkage data in the (**a**,**b**) 20-mer and (**c**,**d**) 10-mer, respectively; contour lines every 1 kcal/mol.

Next, glycosidic linkage dihedral free energies Δ*G*(*ϕ*, *ψ*) were examined (Figure 21 and Table 8). IdoAα1-4GlcNAc glycosidic linkages sampled primarily −*ϕ*, +*ψ* with secondary basins in −*ϕ*, −*ψ* and GlcNAcα1-4IdoA glycosidic linkages sampled conformations in a single basin (+*ϕ*, +*ψ*). We compared our results to those of an NMR and force field study that performed two sets of MD on each of nonsulfated IdoAα1-4GlcNAc and GlcNAcα1-4IdoA disaccharides, one with IdoA restrained to ^1^C_4_ and the other with IdoA restrained to ^2^S_O_, each with a biasing potential on glycosidic linkage dihedrals defined by *ϕ* = H_1_-C_1_-O-C_4_ and *ψ* = C_1_-O-C_4_-H_4_ [30]. If we assume O_5_-C_1_-O-C_4_ = H_1_-C_1_-O-C_4_ - 120° in IdoAα1-4GlcNAc linkages, O_5_-C_1_-O-C_4_ = H_1_-C_1_-O-C_4_ + 120° in GlcNAcα1-4IdoA linkages, and C_1_-O-C_4_-C_3_ = C_1_-O-C_4_-H_4_ + 120° in both linkage types, our data are in close agreement with theirs.

GlcNAcα1-4IdoA glycosidic linkage conformations differ from any other GAG glycosidic linkage conformation because of the orientation of the linker oxygen atom with respect to GlcNAc. Specifically, the oxygen atom on C_1_ of α-GlcNAc is in the opposite orientation as that on C_1_ of any other GAG monosaccharide (Figure 1). The GlcNAcα1-4IdoA linkages have a coiled conformation (Figure S31), which helps explain the high tendency toward compact conformations in nonsulfated heparan compared to other GAGs.

As IdoAα1-4GlcNAc linkages have secondary conformations, we sought to determine if their behavior was random and independent. Δ*G*(*ϕ*, *ψ*) was plotted for each individual IdoAα1-4GlcNAc linkage in each MD run and there did not appear to be any connection between adjacent IdoAα1-4GlcNAc linkages or any patterns across different MD runs. To determine the effects of IdoAα1-4GlcNAc linkages with −*ϕ*, −*ψ* dihedrals on 20-mer backbone flexibility, end-to-end distances of 20-mer conformations with these glycosidic linkage conformations were analyzed. Although these linkage conformations caused a kink in the polymer chain (as with hyaluronan GlcNAcβ1-4GlcA linkages), they were not associated with more compact conformations (Figure S32). In fact, the end-to-end distance distribution of heparan 20-mer conformations with IdoAα1-4GlcNAc linkage −*ϕ*, −*ψ* dihedrals was similar to that of the full MD-generated 20-mer ensemble. This was likely because these linkage conformations occurred infrequently in nonsulfated heparan 20-mer MD, meaning a single heparan 20-mer conformation was not likely to have many kinks resulting from these −*ϕ*, −*ψ* linkage dihedrals. These findings are in line with the literature, which suggests that IdoA ring conformational flexibility in heparin/heparan sulfate oligo- and polysaccharides does not affect glycosidic linkage conformation or overall backbone shape [132].

To determine if there was any interdependency between different IdoA ring puckers and flanking IdoAα1-4GlcNAc linkage geometries in 20-mer MD simulations, conformations of all IdoAα1-4GlcNAc linkages flanking each of ^1^C_4_, ^2^S_O_, ^4^C_1_, and boat/skew-boat (non-^2^S_O_) conformations were analyzed separately (Figure S33). There did not appear to be strong associations between any particular IdoA ring conformation and flanking IdoAα1-4GlcNAc linkage conformation, which is in line with IdoAα1-4GlcNAc disaccharide data from NMR and MD with restrained ^1^C_4_ and ^2^S_O_ IdoA ring conformations and a biasing potential on glycosidic linkage dihedrals [30]. This supports our hypothesis that glycosidic linkages and monosaccharide rings behave independently in MD simulation of nonsulfated heparan 20-mer.

Backbone conformational analysis of nonsulfated heparan 10-mer in MD simulation revealed flexible, compact conformations (Figure S28c and Table 7), as seen in nonsulfated heparan 20-mer MD. Monosaccharide ring and glycosidic linkage conformations in nonsulfated heparan 10-mer MD were similar to those of nonsulfated heparan 20-mer MD. These findings indicate that in MD simulations of nonsulfated heparan 10- and 20-mers, (1) IdoAα1-4GlcNAc glycosidic linkages with −*ϕ*, −*ψ* dihedrals cause a kink in the polymer chain but are rare and thus do not contribute to compact polymer conformations and (2) monosaccharide rings and glycosidic linkages behave randomly and independently.

#### 3.4.2. Construction Algorithm

**Figure 22:**
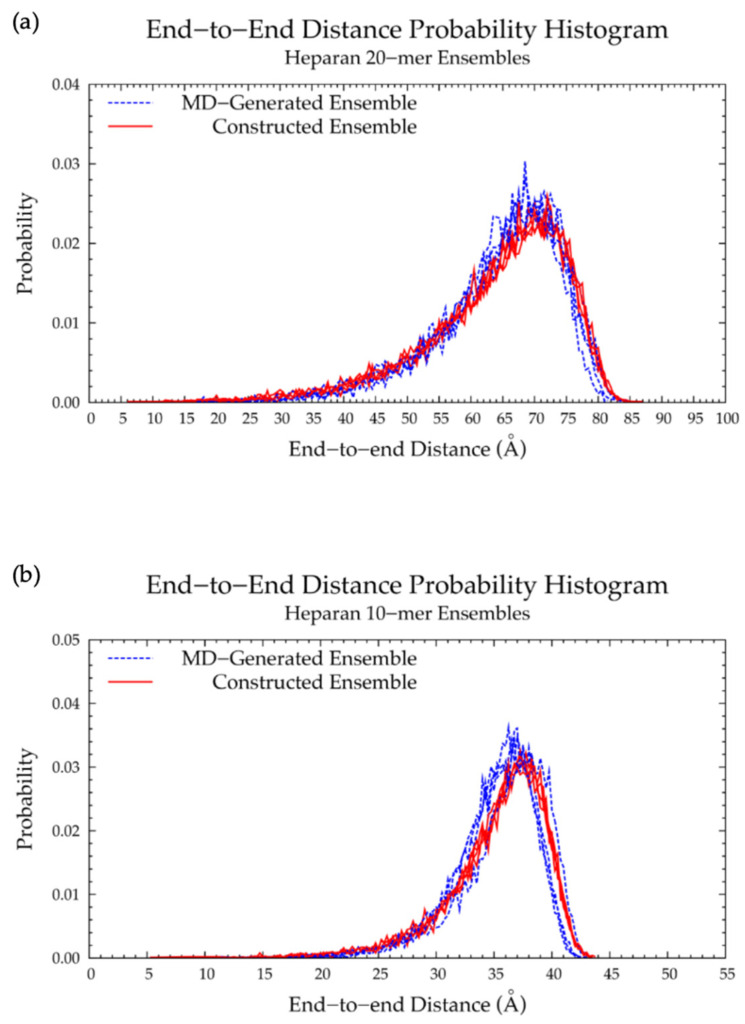
End-to-end distance probability distributions of MD-generated (blue dashed lines) and constructed (red solid lines) nonsulfated heparan ensembles: (**a**) 20-mer and (**b**) 10-mer; probabilities were calculated for end-to-end distances sorted into 0.5 Å bins for the 20-mer and 0.25 Å bins for the 10-mer; each ensemble includes four sets of 10,000 conformations.

A nonsulfated heparan 20-mer ensemble with 40,000 conformations was constructed by the algorithm. Monosaccharide ring C-P parameters (Figure S34) and glycosidic linkage conformations (Figure S35) post-minimization in the constructed ensemble matched the input data (Figure 20a,b and Figure 21a,b), as expected. Additionally, the end-to-end distance distribution and radii of gyration of the constructed ensemble closely resembled those of the MD-generated 20-mer ensemble (Figure 22a and Figure S28a,b and Table 7), demonstrating that our algorithm generates nonsulfated heparan 20-mer ensembles with backbone conformations that mimic backbone flexibility seen in 20-mer MD.

A nonsulfated heparan 10-mer ensemble with 40,000 conformations was also constructed by the algorithm and had end-to-end distances and radii of gyration that matched those of MD-generated nonsulfated heparan 10-mer ensembles (Figure 22b and Figure S28c,d and Table 7). This demonstrates that nonsulfated heparan 20-mer conformations from MD can be used to construct 10-mer ensembles that mimic the backbone flexibility seen in nonsulfated heparan 10-mer MD. 

**Figure 23:**
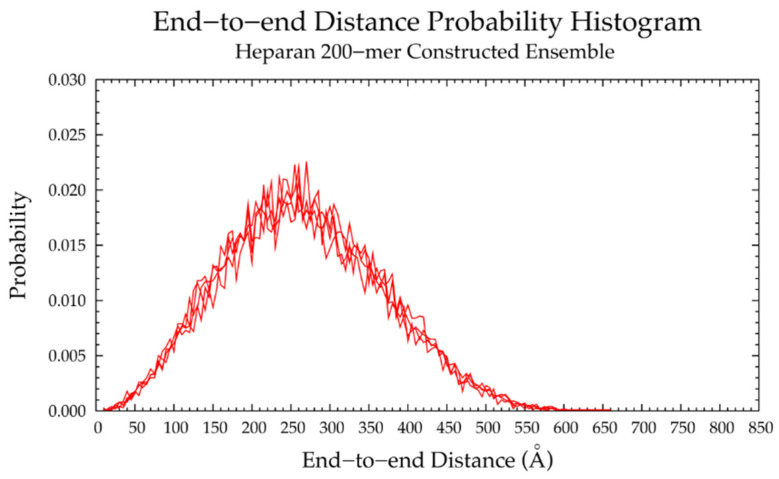
End-to-end distance probability distribution of constructed ensemble of nonsulfated heparan 200-mer; most probable end-to-end distance across all four sets is 260 Å; probabilities were calculated for end-to-end distances sorted into 5 Å bins; the ensemble contains four sets of 10,000 conformations.

The constructed nonsulfated heparan 200-mer ensemble had reasonably expected end-to-end distance probability distributions (Figure 23), i.e., the skewness of the curves shifted toward the right, indicating more compact conformations with increasing polymer length. The heparan 200-mer end-to-end distance distribution curve was qualitatively similar to that of hyaluronan 200-mer (i.e., the probability peak was of similar magnitude and most probable end-to-end distances were similar), which stands to reason, as ring and linkage conformations in nonsulfated heparan 20-mer MD cause more compact backbone conformations, as in hyaluronan 20-mer MD.

## 4. Conclusions

Collectively, our findings support our hypotheses that (1) glycosidic linkages and monosaccharide rings in hyaluronan and nonsulfated dermatan, keratan, and heparan GAG 20-mers behave randomly and independently in MD simulation and (2) using a database of conformations from corresponding GAG 20-mer MD simulations and treating glycosidic linkages and monosaccharide rings independently, our algorithm can efficiently construct conformational ensembles of hyaluronan and nonsulfated dermatan, keratan, and heparan GAG 10- and 20-mers that mimic backbone flexibility observed in corresponding MD simulations. Additionally, our algorithm constructed sets of 10,000 molecular conformations of nonsulfated GAG 200-mers with backbone conformations that we would reasonably expect to see in MD simulation and did so within 12 h. This suggests that the algorithm can generate conformational ensembles of nonsulfated heterogeneous GAG polymers of arbitrary length in under a day. For perspective, 1-µs MD simulations, each producing 10,000 snapshots (i.e., 3-D atomic coordinate sets), of GAG 10- and 20-mers were completed in about 1–2 weeks and 1–2 months, respectively, and using modern GPU-accelerated hardware and software. Furthermore, the algorithm’s potential energy minimization and bond potential energy cutoff criterion exclude nonphysical conformations from constructed ensembles, leaving only conformations with reasonably expected bond energies (Figures S36–S39).

A comparison of the different GAG 20-mer MD simulations provided further insights into the relationship between GAG structure and conformation. For example, no associations between adjacent monosaccharide conformations were seen in any of the GAG 20-mer MD simulations, but differences in conformations of the same monosaccharide ring type in different GAGs were observed. This indicates that the structure of adjacent monosaccharides contributes to ring conformation. Furthermore, IdoA monosaccharides were much more conformationally flexible than GlcA monosaccharides in GAG 10- and 20-mer MD simulations, even though the structural difference between these two monosaccharide types (specifically, the orientation of the carboxylate group on C_5_) is subtle. Interestingly, dermatan and heparan are the only GAGs with IdoA monosaccharides, yet hyaluronan and heparan showed the most conformational flexibility, and heparan showed the greatest tendency toward compact conformations compared to other GAGs. This was likely because of variability in glycosidic linkage conformation, which is independent of monosaccharide ring conformation. Independence of glycosidic linkages and monosaccharide rings was further evidenced by the fact that despite differences in flanking monosaccharide structure in different GAG types, there were similarities between all 1–3 linkage conformations and between all 1–4 linkage conformations, with the exception of GlcNAcα1–4IdoA linkages in heparan.

A comparison of MD-generated GAG 20-mer conformations to those of GAG 10-mers and to existing experimentally-determined conformations of monosaccharides and GAG oligosaccharides supported the use of 20-mer data for the construction of longer GAG polymers. For example, certain IdoA conformations (namely ^4^C_1_ chair) were found more in unbound monosaccharide rings and terminal rings of GAG oligosaccharides than in central rings. Similarly, galactose occasionally sampled non-^4^C_1_ conformations in monosaccharide rings and short oligosaccharides, including nonsulfated keratan 10-mer in MD simulation, but boat/skew-boat conformations of galactose were decreasingly common in central rings of polymers of increasing length. This is in line with our observation that only ^4^C_1_ conformations of galactose were sampled in nonsulfated keratan 20-mer MD. Additionally, some nonhelical glycosidic linkage conformations that caused a kink in the polymer chain, and thus contributed to compact GAG backbone conformations, were found more in GAG 20-mers than in short GAG oligosaccharides. Therefore, conformational landscapes from GAG 20-mer MD simulations likely provide a better representation of conformations of long GAG polymers than existing conformational landscapes of monosaccharides and GAG oligosaccharides.

A comparison of backbone conformational analyses of 200-mers of different GAG types provided insights into structural features and conformational behaviors contributing to GAG polymer backbone flexibility. For example, 200-mer end-to-end distance distribution curves were qualitatively similar in nonsulfated dermatan (Figure 13), keratan (Figure 18), and chondroitin [33], whereas hyaluronan and nonsulfated heparan polymer end-to-end distance distributions (Figure 7 and Figure 23, respectively) showed an even higher tendency toward compact conformations with increasing polymer length than other nonsulfated GAGs. To find possible explanations for this, we compared observations from MD analyses of all GAG types. In hyaluronan 10- and 20-mer MD simulations, (1) GlcNAc conformations were similar to those in nonsulfated keratan and heparan MD, and (2) GlcA conformations were similar to those in nonsulfated chondroitin MD [33]. Furthermore, IdoA rings showed more flexibility in MD simulation of nonsulfated dermatan and heparan than GlcA rings in hyaluronan and chondroitin MD [33]. However, boat/skew-boat conformations (except for ^2^S_O_) in IdoA did not appear to cause more compact polymer backbone conformations than ^1^C_4_, ^2^S_O_, and ^4^C_1_ IdoA conformations, while boat/skew-boat GlcA ring conformations were more highly associated with more compact polymer backbone conformations in hyaluronan and chondroitin [33]. Additionally, in hyaluronan 10- and 20-mer MD simulations, (1) GlcNAcβ1-4GlcA linkages took on the same conformations as Galβ1-4GlcNAc linkages in nonsulfated keratan MD, IdoAα1-4GlcNAc linkages in nonsulfated heparan MD, and GalNAcβ1-4GlcA in nonsulfated chondroitin MD [33] (i.e., primarily −*ϕ*, +*ψ* with a secondary basin at −*ϕ*, −*ψ*) and (2) GlcAβ1-3GlcNAc linkages took on the same conformations as IdoAα1-3GalNAc in nonsulfated dermatan MD, GlcNAcβ1-3Gal in nonsulfated keratan MD, and GlcAβ1-3GalNAc linkages in nonsulfated chondroitin MD [33] (i.e., primarily −*ϕ*, −*ψ*), but with more conformations at −*ϕ*, +*ψ*. All secondary and tertiary conformations (i.e., −*ϕ*, −*ψ* in 1–4 linkages and −*ϕ*, +*ψ* in 1–3 linkages) were nonhelical and caused a kink or slight bend in the polymer chain, and were, therefore, associated with more compact conformations. The differences in energetic stability of secondary and tertiary linkage conformations between different GAG types can be explained by the adjacent monosaccharide structure. The major observation that is unique to hyaluronan is that both glycosidic linkage types take on more of these nonhelical secondary conformations in 10- and 20-mer MD simulations than any of nonsulfated dermatan, keratan, or chondroitin [33] in 10- and 20-mer MD simulations. Heparan is unique, in that it has all 1–4 linkages, while all other GAGs have alternating 1–3 and 1–4 linkages, and it contains α-GlcNAc, which gives unique conformations in GlcNAcα1-4IdoA linkages. These characteristics give nonsulfated heparan a tendency toward more coiled structures than other GAGs.

**Figure 24:**
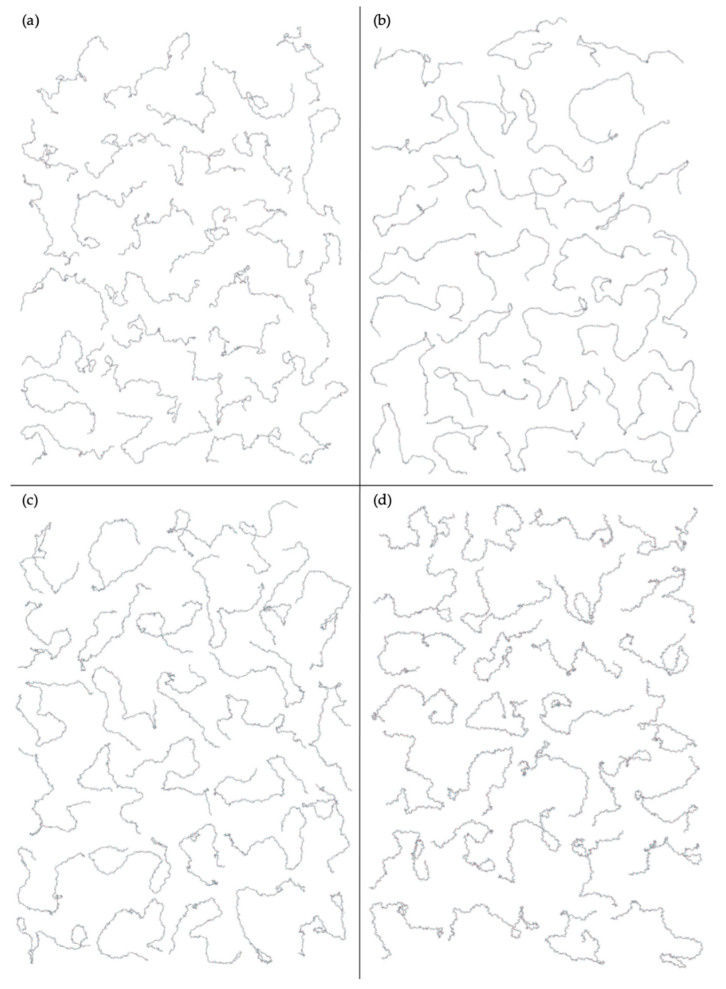
Constructed 200-mer conformations with probable end-to-end distances for each GAG type: (**a**) hyaluronan, (**b**) nonsulfated dermatan, (**c**) nonsulfated keratan, (**d**) nonsulfated heparan; n.b. depth is not shown, so some conformations appear to have overlap.

Based on these observations and the fact that nonsulfated dermatan, keratan, and chondroitin showed more extended polymer backbone conformations than hyaluronan and nonsulfated heparan in MD simulations, it is likely that: (1) monosaccharide structure and conformational flexibility determine adjacent monosaccharide conformational flexibility; (2) although there is a much higher degree of ring flexibility in IdoA than in GlcA, the flexibility of GlcA rings is more highly associated with GAG polymer backbone flexibility than that of IdoA rings; and (3) nonhelical glycosidic linkage conformations, which cause kinks in GAG polymer chains, contribute to more compact GAG polymer backbone conformations and, thus, a higher degree of GAG polymer backbone flexibility. These are valuable insights that would be difficult to obtain from conformational analyses of only solid-state structures and MD-generated conformational ensembles of short GAG oligosaccharides. Other important insights that can be gained from 3-D atomic-resolution conformational ensembles of GAG polymers include potential binding properties, and thus, predictions of binding poses with other biomolecules of interest. Models of long-chain GAG polymers (Figure 24 and [33]) can help characterize complete PGs and GAG-mediated complexes between multiple biomolecules, and consequently, improve our understanding of the bioactivity and function of GAG biopolymers in animal tissue.

## Figures and Tables

**Table 1:** Most Probable End-to-End Distances (*d*) in MD-Generated and Constructed Hyaluronan Ensembles ^1^.

	20-mer Ensembles			10-mer Ensembles		
	MD-Generated *d* (Å)	Constructed *d* (Å)	% Difference	MD-Generated *d* (Å)	Constructed *d* (Å)	% Difference
Run 1	81.5	80.5		43.00	42.25	
Run 2	81.0	78.0		42.50	41.75	
Run 3	83.0	80.5		44.00	42.75	
Run 4	85.0	78.0		43.50	42.75	
All ^2^	80.0	78.0	2.53%	43.50	41.75	4.11%

^1^ Probabilities were calculated for end-to-end distances sorted into 0.5 Å bins for the 20-mer ensembles and 0.25 Å bins for the 10-mer ensembles. ^2^ All = end-to-end distance distribution aggregated across all four runs.

**Table 2:** Hyaluronan Glycosidic Linkage Dihedrals (*ϕ*,*ψ*) ^1^ and Free Energy (Δ*G*(*ϕ*,*ψ*) kcal/mol) Minima.

	MD-Generated 20-mer Ensemble				MD-Generated 10-mer Ensemble				Constructed 20-mer Ensemble			
	GlcAβ1-3 GlcNAc		GlcNAcβ1-4 GlcA		GlcAβ1-3 GlcNAc		GlcNAcβ1-4 GlcA		GlcAβ1-3 GlcNAc		GlcNAcβ1-4 GlcA	
Min	*ϕ*, *ψ*	Δ*G*	*ϕ*, *ψ*	Δ*G*	*ϕ*, *ψ*	Δ*G*	*ϕ*, *ψ*	Δ*G*	*ϕ*, *ψ*	Δ*G*	*ϕ*, *ψ*	Δ*G*
I	−71.25°, −123.75°	0.00	−68.75°, 116.25°	0.00	−71.25°, −123.75°	0.00	−68.75°, 118.75°	0.00	−71.25°, −123.75°	0.00	−71.25°, 116.25°	0.00
II	−53.75°, 91.25°	3.06	−83.75°, −73.75°	1.31	−58.75°, 86.25°	3.08	−81.25°, −76.25°	1.16	−56.25°, 91.25°	3.01	−83.75°, −73.75°	1.29
II’			−58.75°, −33.75°	1.42			−53.75°, −36.25°	1.27			−56.25°, −36.25°	1.40

^1^*ϕ*, *ψ* dihedral angles were sorted into 2.5° bins.

**Table 3:** Most Probable End-to-End Distances (*d*) in MD-Generated and Constructed Nonsulfated Dermatan Ensembles ^1^.

	20-mer Ensembles			10-mer Ensembles		
	MD-Generated *d* (Å)	Constructed *d* (Å)	% Difference	MD-Generated *d* (Å)	Constructed *d* (Å)	% Difference
Run 1	81.5	81.5		42.25	42.75	
Run 2	82.5	83.5		42.50	42.00	
Run 3	83.5	84.0		42.00	42.75	
Run 4	81.0	81.5		42.25	42.75	
All ^2^	83.5	81.5	2.42%	42.25	42.75	1.18%

^1^ Probabilities were calculated for end-to-end distances sorted into 0.5 Å bins for the 20-mer ensembles and 0.25 Å bins for the 10-mer ensembles. ^2^ All = end-to-end distance distribution aggregated across all four runs.

**Table 4:** Nonsulfated Dermatan Glycosidic Linkage Dihedrals (*ϕ*, *ψ*) ^1^ and Free Energy (Δ*G*(*ϕ*, *ψ*) kcal/mol) Minima.

	MD-Generated 20-mer Ensemble				MD-Generated 10-mer Ensemble				Constructed 20-mer Ensemble			
	IdoAβ1-3 GalNAc		GalNAcβ1-4 IdoA		IdoAβ1-3 GalNAc		GalNAcβ1-4 IdoA		IdoAβ1-3 GalNAc		GalNAcβ1-4 IdoA	
Min	*ϕ*, *ψ*	Δ*G*	*ϕ*, *ψ*	Δ*G*	*ϕ*, *ψ*	Δ*G*	*ϕ*, *ψ*	Δ*G*	*ϕ*, *ψ*	Δ*G*	*ϕ*, *ψ*	Δ*G*
I	−66.25°, −123.75°	0.00	−61.25°, 106.25°	0.00	−68.75°, −121.25°	0.00	−58.75°, 108.75°	0.00	−68.75°, −121.25°	0.00	−61.25°, 108.75°	0.00

^1^*ϕ*, *ψ* dihedral angles were sorted into 2.5° bins.

**Table 5:** Most Probable End-to-End Distances (*d*) in MD-Generated and Constructed Nonsulfated Keratan Ensembles ^1^.

	20-mer Ensembles			10-mer Ensembles		
	MD-Generated *d* (Å)	Constructed *d* (Å)	% Difference	MD-Generated *d* (Å)	Constructed *d* (Å)	% Difference
Run 1	88.0	86.0		45.50	45.00	
Run 2	90.0	87.5		46.00	45.75	
Run 3	90.0	87.0		45.75	45.00	
Run 4	89.0	86.0		45.50	45.00	
All ^2^	90.0	87.0	3.39%	45.50	45.00	1.10%

^1^ Probabilities were calculated for end-to-end distances sorted into 0.5 Å bins for the 20-mer ensembles and 0.25 Å bins for the 10-mer ensembles. ^2^ All = end-to-end distance distribution aggregated across all four runs.

**Table 6:** Nonsulfated Keratan Glycosidic Linkage Dihedrals (*ϕ*, *ψ*) ^1^ and Free Energy (Δ*G*(*ϕ*, *ψ*) kcal/mol) Minima.

	MD-Generated 20-mer Ensemble				MD-Generated 10-mer Ensemble				Constructed 20-mer Ensemble			
	Galβ1-4 GlcNAc		GlcNAcβ1-3 Gal		Galβ1-4 GlcNAc		GlcNAcβ1-3 Gal		Galβ1-4 GlcNAc		GlcNAcβ1-3 Gal	
Min	*ϕ*, *ψ*	Δ*G*	*ϕ*, *ψ*	Δ*G*	*ϕ*, *ψ*	Δ*G*	*ϕ*, *ψ*	Δ*G*	*ϕ*, *ψ*	Δ*G*	*ϕ*, *ψ*	Δ*G*
I	−73.75°, 116.25°	0.00	−81.25°, −156.25°	0.00	−71.25°, 116.25°	0.00	−81.25°, −156.25°	0.00	−71.25°, 118.75°	0.00	−81.25°, −158.75°	0.00
II	−56.25°, −31.25°	1.75			−56.25°, −36.25°	1.67			−53.75°, −33.75°	1.75		

^1^*ϕ*, *ψ* dihedral angles were sorted into 2.5° bins.

**Table 7:** Most Probable End-to-End Distances (*d*) in MD-Generated and Constructed Nonsulfated Heparan Ensembles ^1^.

	20-mer Ensembles			10-mer Ensembles		
	MD-Generated *d* (Å)	Constructed *d* (Å)	% Difference	MD-Generated *d* (Å)	Constructed *d* (Å)	% Difference
Run 1	68.5	70.0		37.00	37.25	
Run 2	68.5	72.0		36.25	38.25	
Run 3	67.5	67.5		37.50	37.25	
Run 4	66.5	71.5		36.75	36.75	
All ^2^	68.5	72.0	4.98%	37.00	37.25	0.67%

^1^ Probabilities were calculated for end-to-end distances sorted into 0.5 Å bins for the 20-mer ensembles and 0.25 Å bins for the 10-mer ensembles. ^2^ All = end-to-end distance distribution aggregated across all four runs.

**Table 8:** Nonsulfated Heparan Glycosidic Linkage Dihedrals (*ϕ*, *ψ*) ^1^ and Free Energy (Δ*G*(*ϕ*, *ψ*) kcal/mol) Minima.

	MD-Generated 20-mer Ensemble				MD-Generated 10-mer Ensemble				Constructed 20-mer Ensemble			
	IdoAα1-4 GlcNAc		GlcNAcα1-4 IdoA		IdoAα1-4 GlcNAc		GlcNAcα1-4 IdoA		IdoAα1-4 GlcNAc		GlcNAcα1-4 IdoA	
Min	*ϕ*, *ψ*	Δ*G*	*ϕ*, *ψ*	Δ*G*	*ϕ*, *ψ*	Δ*G*	*ϕ*, *ψ*	Δ*G*	*ϕ*, *ψ*	Δ*G*	*ϕ*, *ψ*	Δ*G*
I	−73.75°, 128.75°	0.00	76.25°, 88.75°	0.00	−76.25°, 128.75°	0.00	73.75°, 88.75°	0.00	−76.25°, 128.75°	0.00	−71.25°, 116.25°	0.00
II	−103.75°, −68.75°	3.13			−103.75°, −63.75°	2.66			−98.75°, −68.75°	3.23		
II’	−66.25°, −31.25°	3.38			−66.25°, −33.75°	2.99			−61.25°, −36.25°	3.34		

^1^*ϕ*, *ψ* dihedral angles were sorted into 2.5° bins.

## Supplementary Materials

The following are available online https://www.mdpi.com/1422-0067/21/20/7699/s1, Table S1: Most Probable End-to-End Distances (*d*) in MD-Generated 20-mer Conformations with Glycosidic Linkage Conformations in Secondary Basins, Table S2: Percent of Occurrences of Different IdoA Ring Puckers in Nonsulfated Dermatan MD Simulations, Table S3: Percent of Occurrences of Different IdoA Ring Puckers in Nonsulfated Heparan MD Simulations, Figure S1: Constructed hyaluronan 20-mer conformation with a pierced ring, Figure S2: Scatterplots of radius of gyration as a function of end-to-end distance in MD-generated and constructed hyaluronan ensembles, Figure S3: System potential energy probability distribution of the MD-generated hyaluronan 20-mer ensemble, Figure S4: C-P plots and C-P parameter *θ* timeseries for each GlcNAc monosaccharide ring in the MD-generated hyaluronan 20-mer ensemble, Figure S5: C-P plots and C-P parameter *θ* timeseries for each GlcA monosaccharide ring in the MD-generated hyaluronan 20-mer ensemble, Figure S6: Scatterplot of radius of gyration as a function of end-to-end distance in MD-generated hyaluronan 20-mer conformations with non-^4^C_1_ ring puckers, Figure S7: Δ*G(ϕ,ψ)* plots for each glycosidic linkage in the MD-generated hyaluronan 20-mer ensemble, Figure S8: MD snapshots of hyaluronan 20-mer GlcAβ1-3GlcNAc glycosidic linkages with dihedrals in different basins and end-to-end distance distributions of 20-mer conformations with linkages in secondary basin, Figure S9: Δ*G(ϕ,ψ)* plots for glycosidic linkages flanking non-^4^C_1_ ring puckers in the MD-generated hyaluronan 20-mer ensemble, Figure S10: MD snapshots of hyaluronan 20-mer GlcNAcβ1-4GlcA glycosidic linkages with dihedrals in different basins and end-to-end distance distributions of 20-mer conformations with linkages in secondary basin, Figure S11: C-P plots for GlcNAc and GlcA in the constructed hyaluronan 20-mer ensemble, Figure S12: Δ*G*(*ϕ*, *ψ*) for aggregated glycosidic linkage data from the constructed hyaluronan 20-mer ensemble, Figure S13: Scatterplots of radius of gyration as a function of end-to-end distance in MD-generated and constructed nonsulfated dermatan ensembles, Figure S14: C-P parameter *θ* timeseries for each GalNAc monosaccharide ring in the MD-generated nonsulfated dermatan 20-mer ensemble, Figure S15: End-to-end distance distributions of MD-generated nonsulfated dermatan 20-mer conformations with boat/skew-boat ring puckers that cause a kink in the polymer chain and ^2^S_O_ conformations, Figure S16: C-P plots and C-P parameter *θ* timeseries for each IdoA monosaccharide ring in the MD-generated nonsulfated dermatan 20-mer ensemble, Figure S17: C-P plots and C-P parameter *θ* timeseries for each IdoA monosaccharide ring in the MD-generated nonsulfated dermatan 10-mer ensemble, Figure S18: C-P plots for GalNAc and IdoA in the constructed nonsulfated dermatan 20-mer ensemble, Figure S19: Δ*G*(*ϕ*, *ψ*) for aggregated glycosidic linkage data from the constructed nonsulfated dermatan 20-mer ensemble, Figure S20: Scatterplots of radius of gyration as a function of end-to-end distance in MD-generated and constructed nonsulfated keratan ensembles, Figure S21: C-P plots and C-P parameter *θ* timeseries for each GlcNAc monosaccharide ring in the MD-generated nonsulfated keratan 20-mer ensemble, Figure S22: Scatterplot of radius of gyration as a function of end-to-end distance in MD-generated nonsulfated keratan 20-mer conformations with non-^4^C_1_ ring puckers, Figure S23: MD snapshots of nonsulfated keratan 20-mer Galβ1-4GlcNAc glycosidic linkages with dihedrals in different basins and end-to-end distance distributions of 20-mer conformations with linkages in secondary and tertiary basins, Figure S24: MD snapshots of nonsulfated keratan 20-mer GlcNAcβ1-3Gal glycosidic linkages with dihedrals in different basins and end-to-end distance distributions of 20-mer conformations with linkages in secondary and tertiary basins, Figure S25: C-P plots and C-P parameter *θ* timeseries for each Gal monosaccharide ring in the MD-generated nonsulfated keratan 10-mer ensemble, Figure S26: C-P plots for GlcNAc and Gal in the constructed nonsulfated keratan 20-mer ensemble, Figure S27: Δ*G*(*ϕ*, *ψ*) for aggregated glycosidic linkage data from the constructed nonsulfated keratan 20-mer ensemble, Figure S28: Scatterplots of radius of gyration as a function of end-to-end distance in MD-generated and constructed nonsulfated heparan ensembles, Figure S29: C-P parameter *θ* timeseries for each IdoA monosaccharide ring in the MD-generated nonsulfated heparan 20-mer ensemble, Figure S30: End-to-end distance distributions of MD-generated nonsulfated heparan 20-mer conformations with boat/skew-boat ring puckers that cause a kink in the polymer chain and ^2^S_O_ conformations, Figure S31: Snapshot of nonsulfated heparan 20-mer MD-generated ensemble highlighting GlcNAcα1-4IdoA linkages, Figure S32: End-to-end distance distributions of MD-generated nonsulfated heparan 20-mer conformations with linkage dihedrals in secondary basins, Figure S33: Δ*G(ϕ,ψ)* plots for IdoAα1-4GlcNAc linkages flanking different IdoA conformations in the MD-generated nonsulfated heparan 20-mer ensemble, Figure S34: C-P plots for GlcNAc and IdoA in the constructed nonsulfated heparan 20-mer ensemble, Figure S35: Δ*G*(*ϕ*, *ψ*) for aggregated glycosidic linkage data from the constructed nonsulfated heparan 20-mer ensemble, Figure S36: Bond potential energy probability distributions from constructed hyaluronan ensembles, Figure S37: Bond potential energy probability distributions from constructed nonsulfated dermatan ensembles, Figure S38: Bond potential energy probability distributions from constructed nonsulfated keratan ensembles, Figure S39: Bond potential energy probability distributions from constructed nonsulfated heparan ensembles.

## Abbreviations

PG	Proteoglycan
GAG	Glycosaminoglycan
MD	Molecular dynamics
GlcA	Glucuronate
IdoA	Iduronate
Gal	Galactose
GlcNAc	N-acetylglucosamine
GalNAc	N-acetylgalactosamine
