# Supplementary material for: Constructing 3-Dimensional Atomic-Resolution Models of Nonsulfated Glycosaminoglycans with Arbitrary Lengths Using Conformations from Molecular Dynamics
Source: Int J Mol Sci. 2020 Oct 18;21(20):7699. doi: 10.3390/ijms21207699 (PMC7589010; doi:10.3390/ijms21207699)
Supplement: Supplementary file 1 [file ijms-21-07699-s001.pdf]

Supporting Information for:

Constructing 3-Dimensional Atomic-Resolution Models of Nonsulfated Glycosaminoglycans with Arbitrary Lengths Using Conformations from Molecular Dynamics

Elizabeth K. Whitmore [1,2], Devon Martin[1,2], and Olgun Guvench [1,2,*]

[1] Department of Pharmaceutical Sciences and Administration, University of New England School of Pharmacy, 716 Stevens Avenue, Portland, Maine, 04103, USA; ewhitmore@une.edu (E.K.W.); dmartin11@une.edu (D.M.)
[2] Graduate School of Biomedical Science and Engineering, University of Maine, 5775 Stodder Hall, Orono, Maine, 04469, USA
[*] Correspondence: oguvench@une.edu; Tel.: +01-207-221-4171

Table S1. Most Probable End-to-End Distances (*d*) in MD-Generated 20-mer Conformations with Glycosidic Linkage Conformations in Secondary Basins [1]

	**Hyaluronan**		**Non-Sulfated Keratan**	
	**GlcAβ1-3GlcNAc** **-ϕ, +ψ** ***d* (Å)**	**GlcNAcβ1-4GlcA** **-ϕ, -ψ** ***d* (Å)**	**Galβ1-4GlcNAc** **-ϕ, -ψ** ***d* (Å)**	**GlcNAcβ1-3Gal** **-ϕ, +ψ** ***d* (Å)**
**Run 1**	81.5	74.0	88.5	79.5
**Run 2**	73.0	73.5	85.0	84.0
**Run 3**	61.0	74.0	84.5	88.5
**Run 4**	77.0	76.5	84.5	73.5
**All [2]**	66.0	74.0	84.5	84.0

[1]Probabilities were calculated for end-to-end distances sorted into 0.5 Å bins. [2]All = end-to-end distance distribution aggregated across all four runs.

Table S2. Percent of Occurrences of Different IdoA Ring Puckers in Non-Sulfated Dermatan MD Simulations

	**20-mer Ensemble**	**10-mer Ensemble**
**$^{1}C_{4}$**	68.727%	70.100%
**$^{4}C_{1}$**	7.062%	9.025%
**$B_{1,4}$**	0.018%	0.005%
**$^{5}S_{1}$**	0.740%	0.685%
**$^{2,5}B$**	3.213%	2.610%
**$^{2}S_{O}$**	19.702%	17.065%
**$B_{3,O}$**	0.080%	0.070%
**$^{1}S_{3}$**	0.125%	0.125%
**$^{1,4}B$**	0.178%	0.115%
**$^{1}S_{5}$**	0.155%	0.200%

Table S3. Percent of Occurrences of Different IdoA Ring Puckers in Non-Sulfated Heparan MD Simulations

	**20-mer Ensemble**	**10-mer Ensemble**
$^{1}C_{4}$	54.222%	64.645%
$^{4}C_{1}$	4.410%	6.730%
$^{3}S_{1}$	0.003%	0.000%
$B_{1,4}$	0.085%	0.035%
$^{5}S_{1}$	4.120%	3.110%
$^{2,5}B$	12.510%	8.550%
$^{2}S_{O}$	24.505%	16.520%
$B_{3,O}$	0.008%	0.000%
$^{1}S_{3}$	0.035%	0.085%
$^{1,4}B$	0.047%	0.155%
$^{1}S_{5}$	0.055%	0.170%

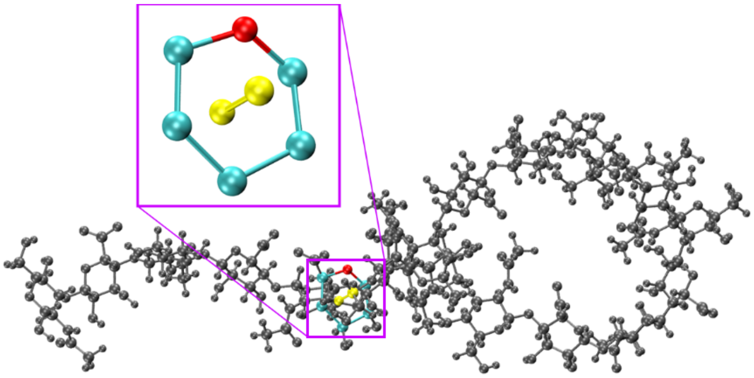

Figure S1. Excluded constructed hyaluronan 20-mer conformation with GlcA 20 ring pierced by C_1-O_3 bond in GlcA 6 β1-3 GlcNAc 5 linkage post-minimization (E_b = 690.5 kcal/mol; closeup shows atoms involved in the ring pierce); $E_{b,\ cutoff}$ = 128.5 kcal/mol.

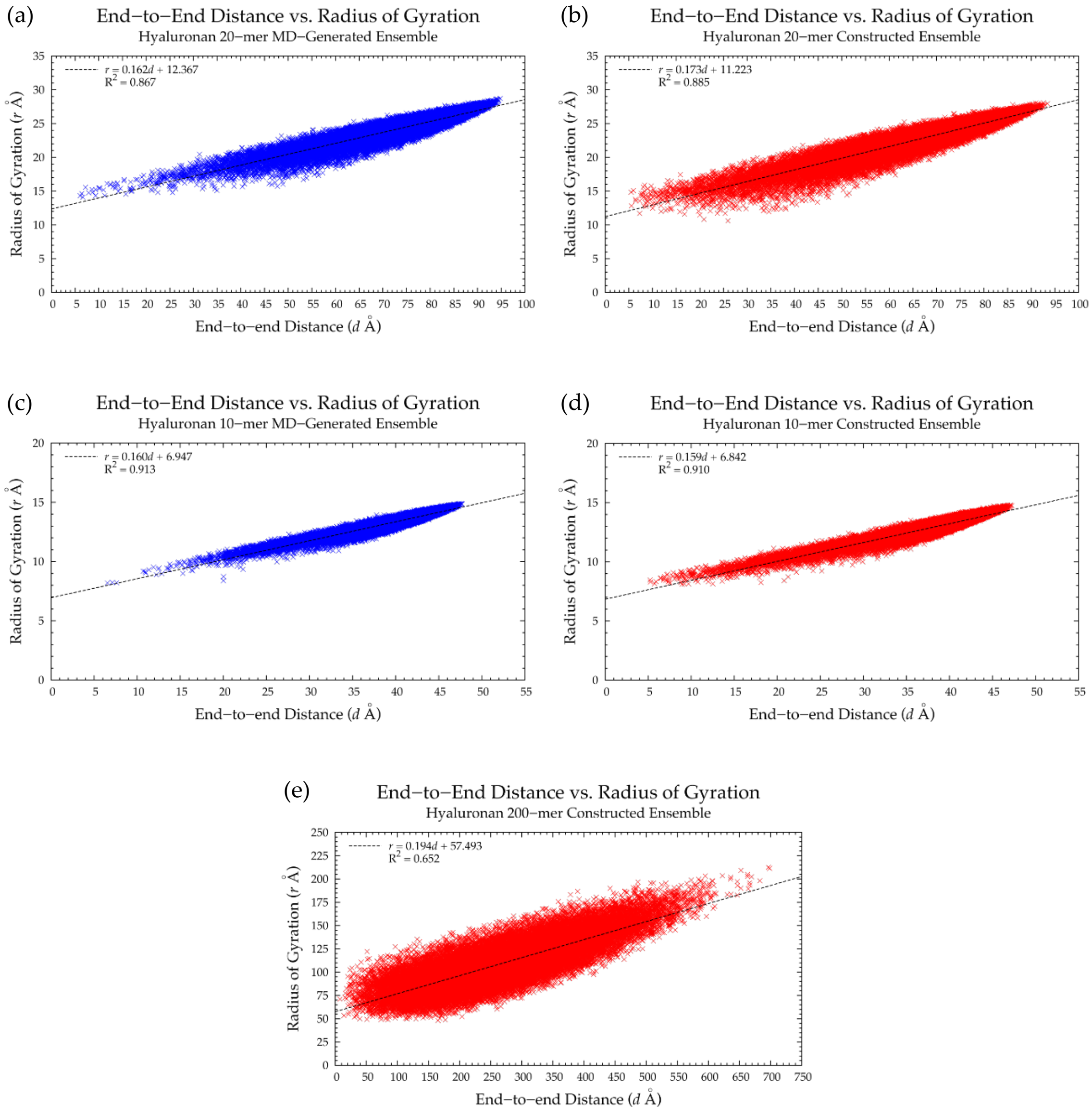

Figure S2. Scatterplots of radius of gyration as a function of end-to-end distance in MD-generated and constructed ensembles of hyaluronan (a,b) 20-mer and (c,d) 10-mer, respectively, and (e) constructed ensemble of hyaluronan 200-mer; each plot has 40,000 samples and shows linear regression and R^2.

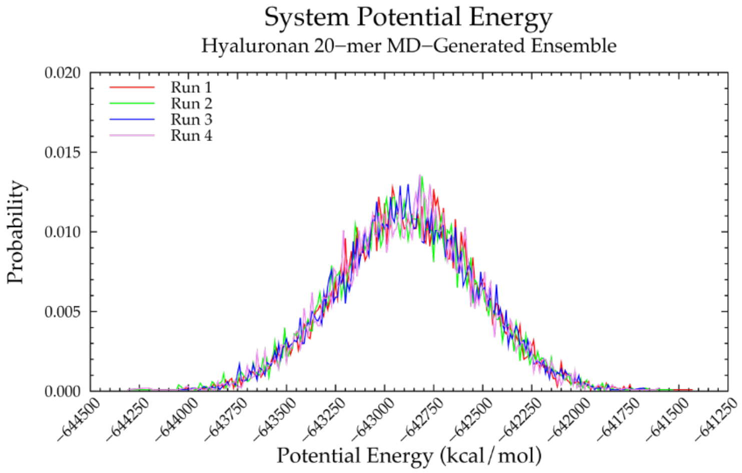

Figure S3. System potential energy probability distribution of the MD-generated hyaluronan 20-mer ensemble; each MD run is represented by a different color.

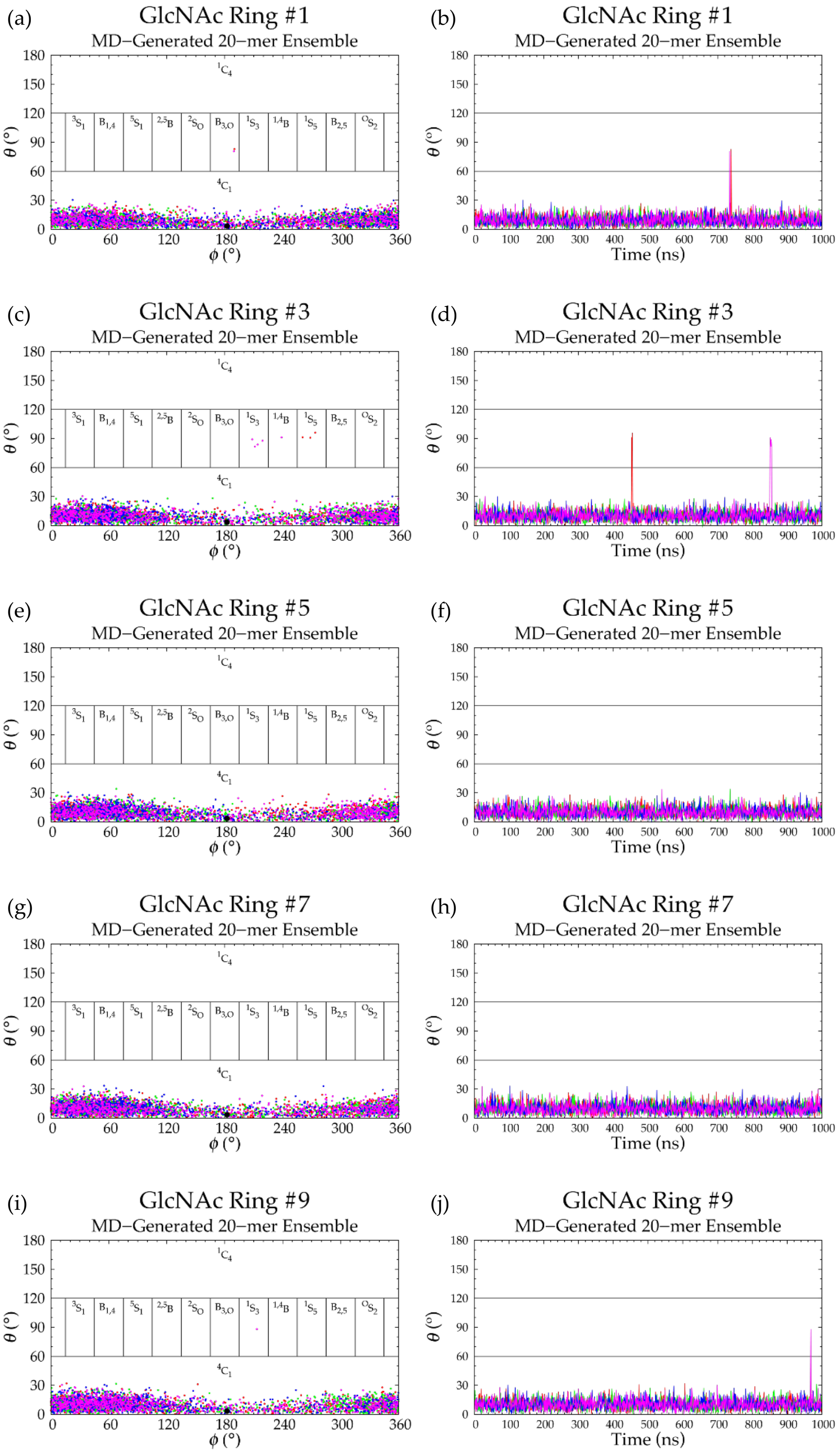

(a)
GlcNAc Ring #1
MD−Generated 20−mer Ensemble
(b)
GlcNAc Ring #1
MD−Generated 20−mer Ensemble
(c)
GlcNAc Ring #3
MD−Generated 20−mer Ensemble
(d)
GlcNAc Ring #3
MD−Generated 20−mer Ensemble
(e)
GlcNAc Ring #5
MD−Generated 20−mer Ensemble
(f)
GlcNAc Ring #5
MD−Generated 20−mer Ensemble
(g)
GlcNAc Ring #7
MD−Generated 20−mer Ensemble
(h)
GlcNAc Ring #7
MD−Generated 20−mer Ensemble
(i)
GlcNAc Ring #9
MD−Generated 20−mer Ensemble
(j)
GlcNAc Ring #9
MD−Generated 20−mer Ensemble
θ (°)
ϕ (°)
Time (ns)

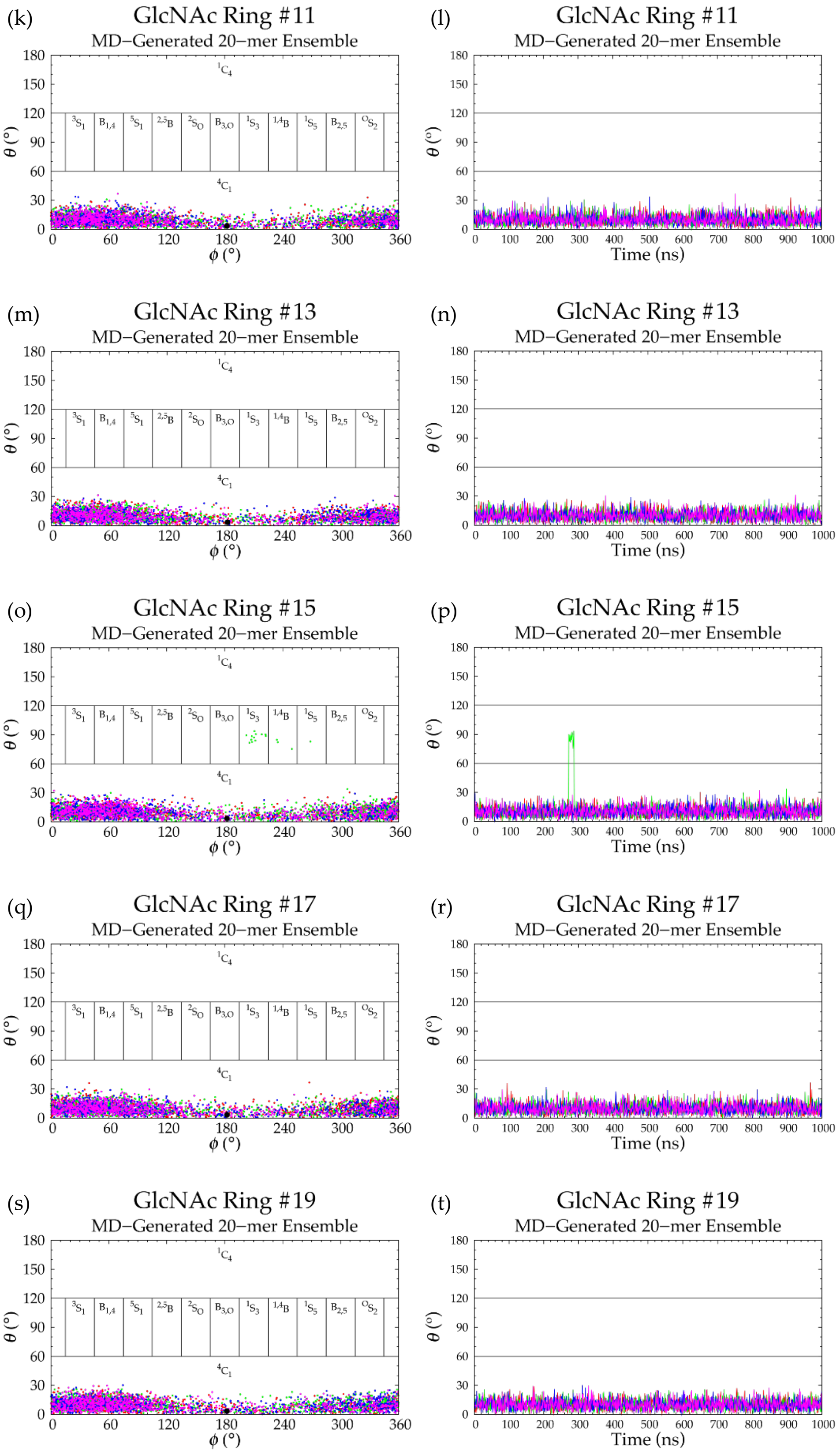
(k)
GlcNAc Ring #11
MD−Generated 20−mer Ensemble
(l)
GlcNAc Ring #11
MD−Generated 20−mer Ensemble
(m)
GlcNAc Ring #13
MD−Generated 20−mer Ensemble
(n)
GlcNAc Ring #13
MD−Generated 20−mer Ensemble
(o)
GlcNAc Ring #15
MD−Generated 20−mer Ensemble
(p)
GlcNAc Ring #15
MD−Generated 20−mer Ensemble
(q)
GlcNAc Ring #17
MD−Generated 20−mer Ensemble
(r)
GlcNAc Ring #17
MD−Generated 20−mer Ensemble
(s)
GlcNAc Ring #19
MD−Generated 20−mer Ensemble
(t)
GlcNAc Ring #19
MD−Generated 20−mer Ensemble
$^1C_4$
$^3S_1$ $B_{1,4}$ $^5S_1$ $^{2,5}B$ ^2S_O $B_{3,O}$ $^1S_3$ $^{1,4}B$ $^1S_5$ $B_{2,5}$ $^OS_2$
$^4C_1$
θ (°)
ϕ (°)
Time (ns)

Figure S4. (a,c,e,g,i,k,m,o,q,s) Cremer-Pople plots and (b,d,f,h,j,l,n,p,r,t) Cremer-Pople parameter θ timeseries for each GlcNAc monosaccharide ring in the MD-generated hyaluronan 20-mer ensemble; monosaccharides are numbered from reducing to non-reducing end; each of the 4 runs is represented by different color.

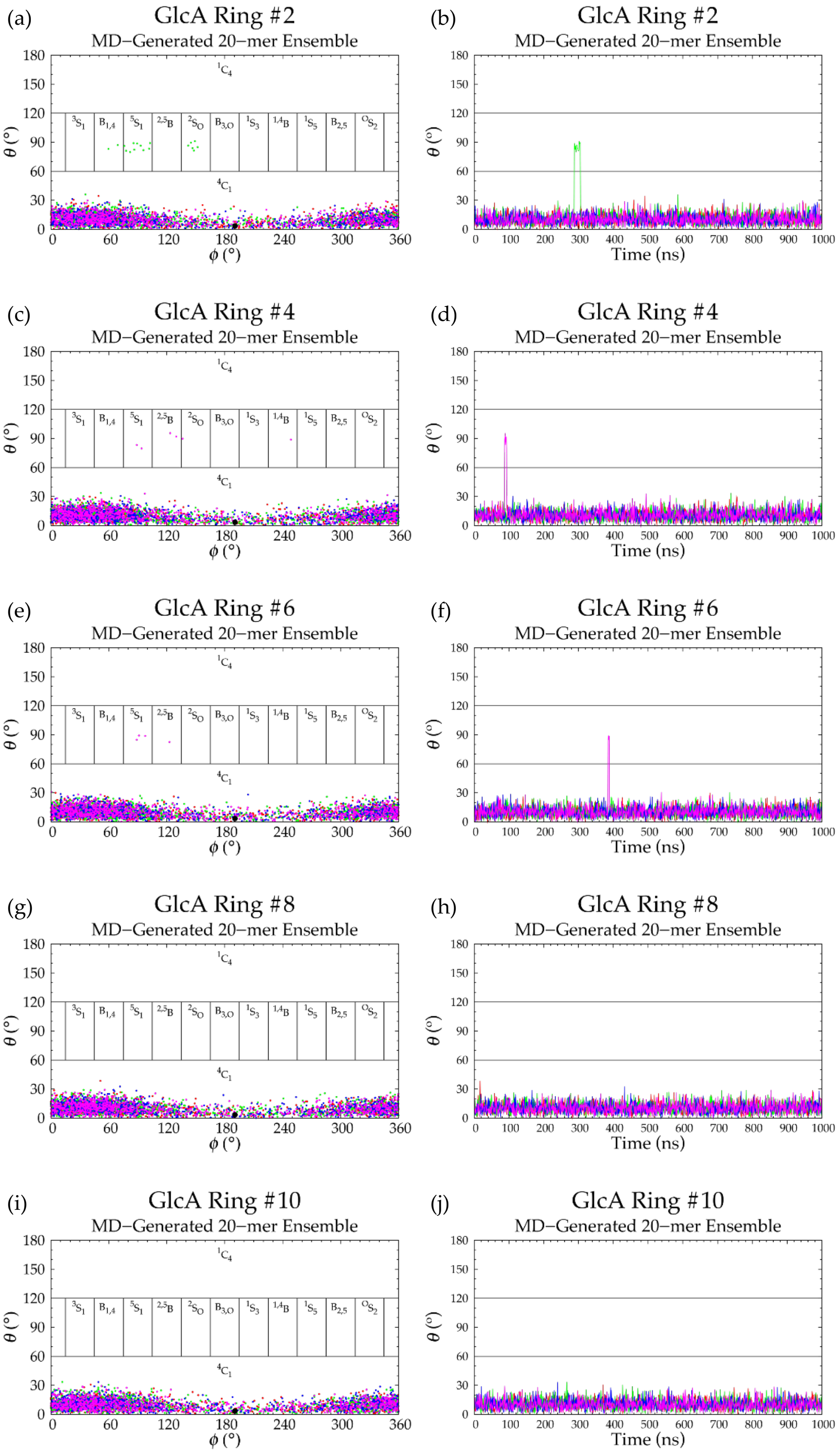

(a)
GlcA Ring #2
MD−Generated 20−mer Ensemble
(b)
GlcA Ring #2
MD−Generated 20−mer Ensemble
(c)
GlcA Ring #4
MD−Generated 20−mer Ensemble
(d)
GlcA Ring #4
MD−Generated 20−mer Ensemble
(e)
GlcA Ring #6
MD−Generated 20−mer Ensemble
(f)
GlcA Ring #6
MD−Generated 20−mer Ensemble
(g)
GlcA Ring #8
MD−Generated 20−mer Ensemble
(h)
GlcA Ring #8
MD−Generated 20−mer Ensemble
(i)
GlcA Ring #10
MD−Generated 20−mer Ensemble
(j)
GlcA Ring #10
MD−Generated 20−mer Ensemble
$^1C_4$
$^3S_1$ $B_{1,4}$ $^5S_1$ $^{2,5}B$ ^2S_O $B_{3,O}$ $^1S_3$ $^{1,4}B$ $^1S_5$ $B_{2,5}$ $^OS_2$
$^4C_1$
θ (°)
ϕ (°)
Time (ns)

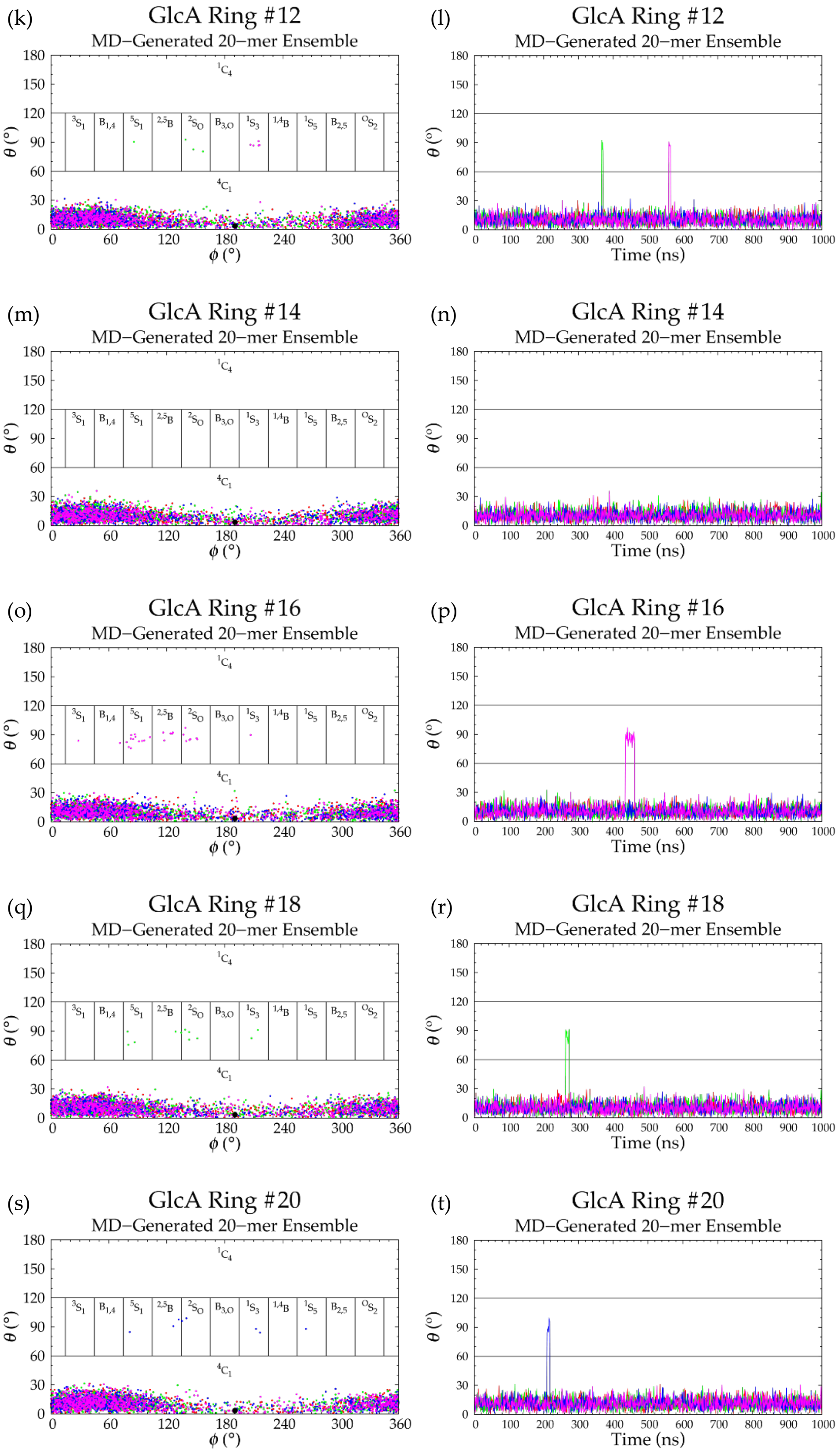
(k)
GlcA Ring #12
MD−Generated 20−mer Ensemble
(l)
GlcA Ring #12
MD−Generated 20−mer Ensemble
(m)
GlcA Ring #14
MD−Generated 20−mer Ensemble
(n)
GlcA Ring #14
MD−Generated 20−mer Ensemble
(o)
GlcA Ring #16
MD−Generated 20−mer Ensemble
(p)
GlcA Ring #16
MD−Generated 20−mer Ensemble
(q)
GlcA Ring #18
MD−Generated 20−mer Ensemble
(r)
GlcA Ring #18
MD−Generated 20−mer Ensemble
(s)
GlcA Ring #20
MD−Generated 20−mer Ensemble
(t)
GlcA Ring #20
MD−Generated 20−mer Ensemble
$^{1}C_{4}$
$^{3}S_{1}$ $B_{1,4}$ $^{5}S_{1}$ $^{2,5}B$ $^{2}S_{O}$ $B_{3,O}$ $^{1}S_{3}$ $^{1,4}B$ $^{1}S_{5}$ $B_{2,5}$ $^{O}S_{2}$
$^{4}C_{1}$
θ (°)
ϕ (°)
Time (ns)
0 30 60 90 120 150 180
0 60 120 180 240 300 360
0 100 200 300 400 500 600 700 800 900 1000

Figure S5. (a,c,e,g,i,k,m,o,q,s) Cremer-Pople plots and (b,d,f,h,j,l,n,p,r,t) Cremer-Pople parameter θ timeseries for each GlcA monosaccharide ring in the MD-generated hyaluronan 20-mer ensemble; monosaccharides are numbered from reducing to non-reducing end; each of the 4 runs is represented by different color.

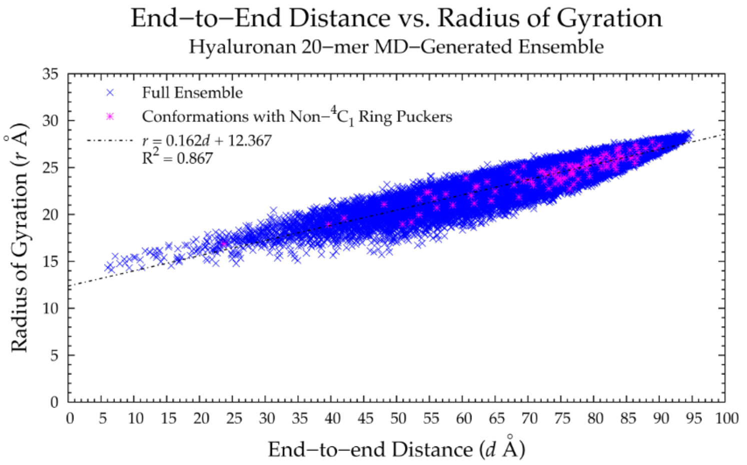

Figure S6. Scatterplot of radius of gyration as a function of end-to-end distance in MD-generated hyaluronan 20-mer conformations with non-$^4C_1$ ring puckers (pink) overlaid on data for full MD-generated hyaluronan 20-mer ensemble (blue) and corresponding linear regression (*Figure S1a*).

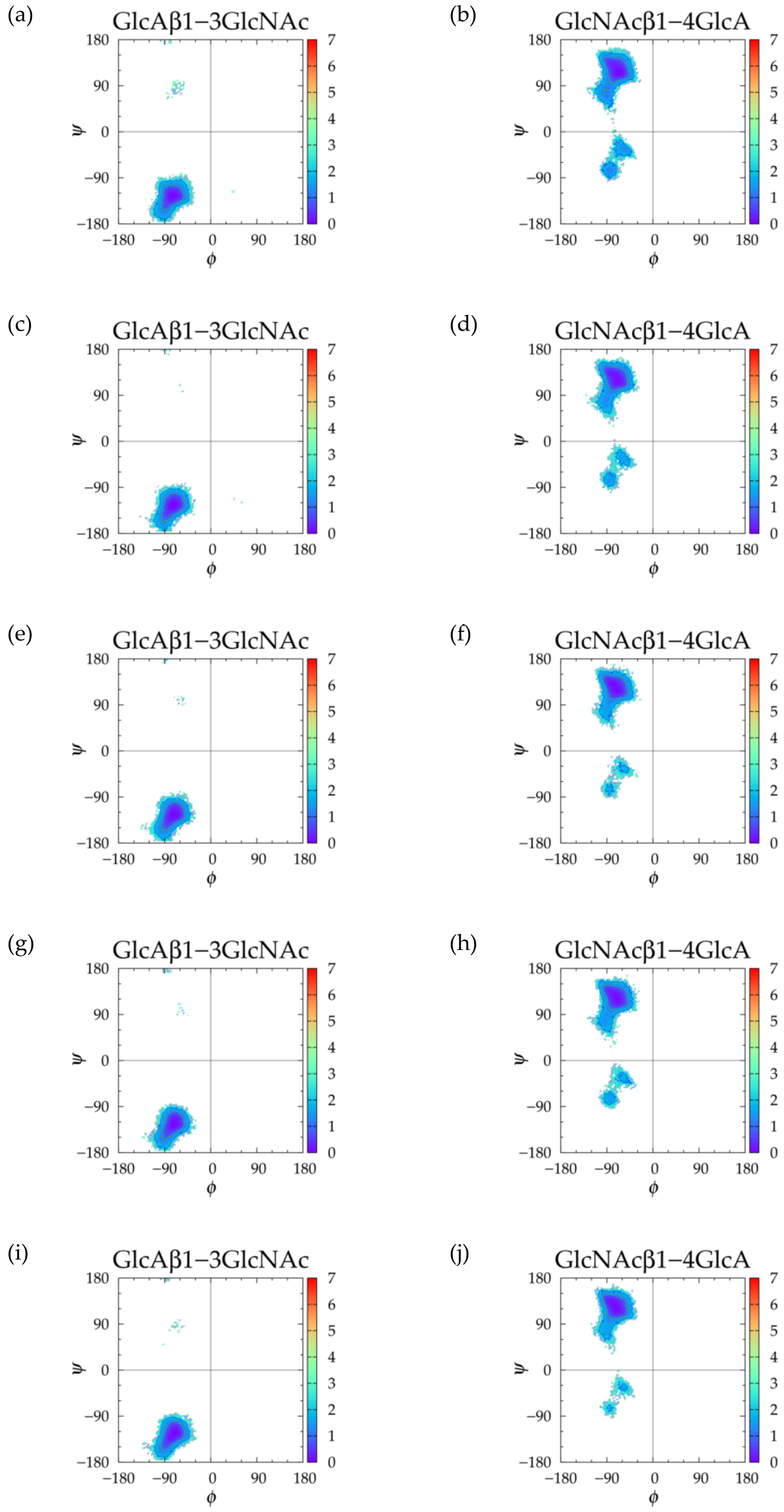
(a)
GlcAβ1−3GlcNAc
(b)
GlcNAcβ1−4GlcA
(c)
GlcAβ1−3GlcNAc
(d)
GlcNAcβ1−4GlcA
(e)
GlcAβ1−3GlcNAc
(f)
GlcNAcβ1−4GlcA
(g)
GlcAβ1−3GlcNAc
(h)
GlcNAcβ1−4GlcA
(i)
GlcAβ1−3GlcNAc
(j)
GlcNAcβ1−4GlcA
ψ
ϕ
180
90
0
−90
−180
0
1
2
3
4
5
6
7

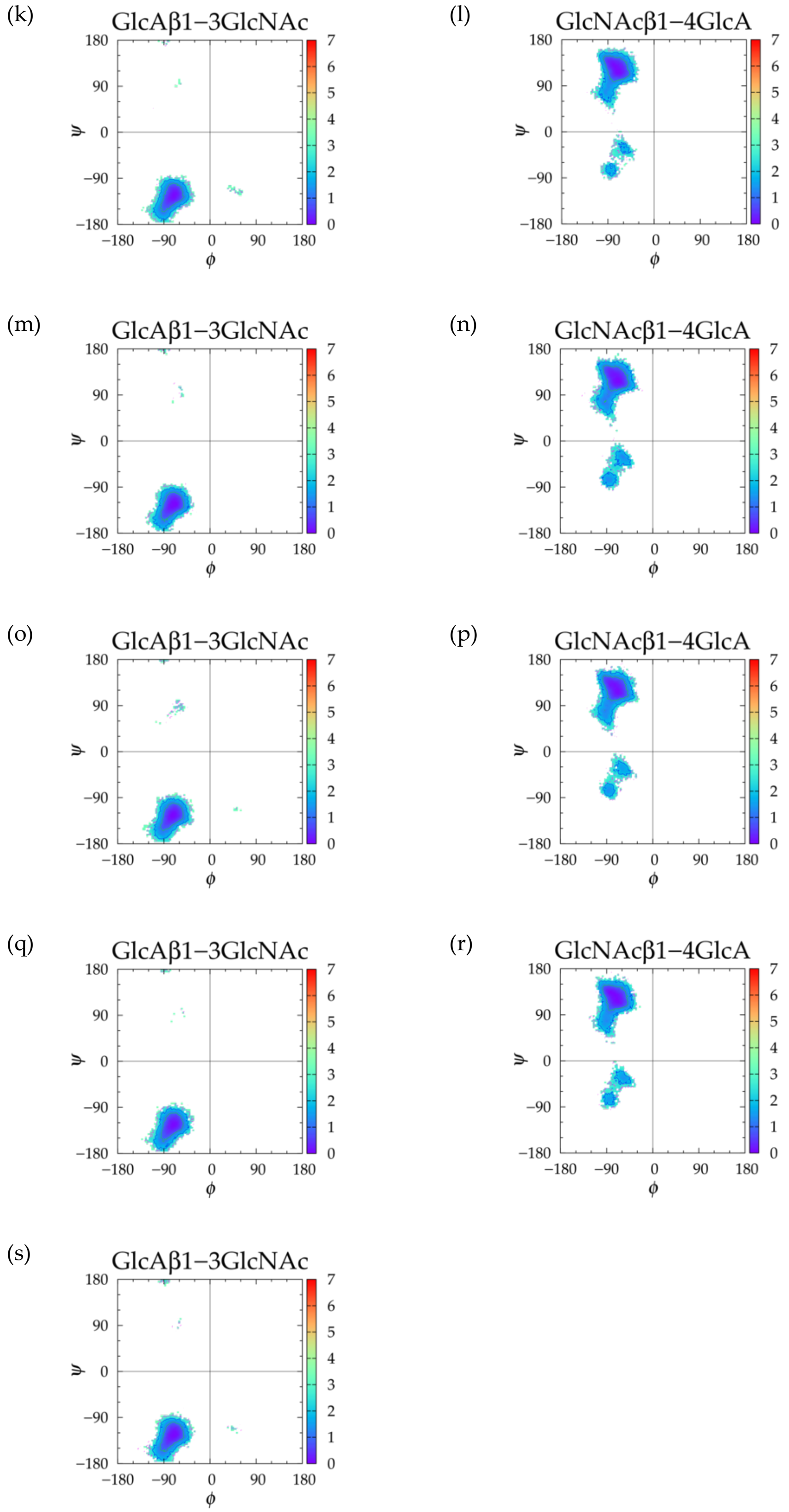

(k)
GlcAβ1−3GlcNAc
(l)
GlcNAcβ1−4GlcA
(m)
GlcAβ1−3GlcNAc
(n)
GlcNAcβ1−4GlcA
(o)
GlcAβ1−3GlcNAc
(p)
GlcNAcβ1−4GlcA
(q)
GlcAβ1−3GlcNAc
(r)
GlcNAcβ1−4GlcA
(s)
GlcAβ1−3GlcNAc
ψ
ϕ
−180 −90 0 90 180
0 1 2 3 4 5 6 7

Figure S7. $\Delta G(\phi, \psi)$ plots for each glycosidic linkage in the MD-generated hyaluronan 20-mer ensemble; (a) GlcA2→GlcNAc1, (b) GlcNAc3→GlcA2, (c) GlcA4→GlcNAc3, (d) GlcNAc5→GlcA4, (e) GlcA6→GlcNAc5, (f) GlcNAc7→GlcA6, (g) GlcA8→GlcNAc7, (h) GlcNAc9→GlcA8, (i) GlcA10→GlcNAc9, (j) GlcNAc11→GlcA10, (k) GlcA12→GlcNAc11, (l) GlcNAc13→GlcA12, (m) GlcA14→GlcNAc13, (n) GlcNAc15→GlcA14, (o) GlcA16→GlcNAc15, (p) GlcNAc17→GlcA16, (q) GlcA18→GlcNAc17, (r) GlcNAc19→GlcA18, and (s) GlcA20→GlcNAc19; monosaccharides are numbered from reducing to non-reducing end; ϕ, ψ separated into 2.5° bins.

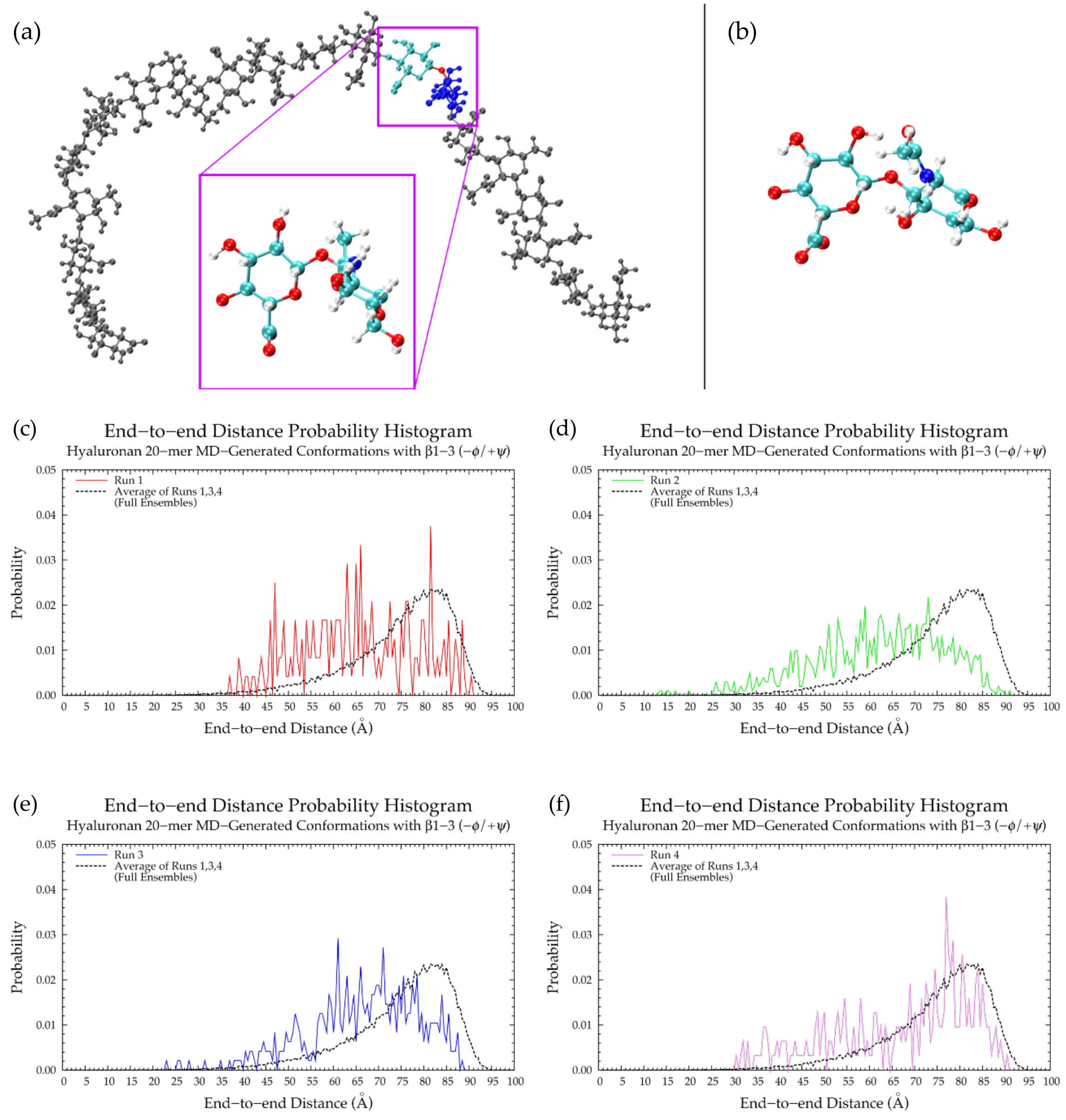

Figure S8. Snapshots from hyaluronan 20-mer MD: (a) 20-mer conformation with GlcA 8 (cyan) β1-3 GlcNAc 7 (blue) linkage dihedrals near $\Delta G(\phi, \psi)$ min II (ϕ = -52.1° and ψ = +91.0°), which causes a kink (linker oxygen is red), and closeup of this disaccharide unit, (b) closeup of the same disaccharide unit with linkage dihedrals near $\Delta G(\phi, \psi)$ min I (ϕ = -70.9° and ψ = -119.1°). (c-f) End-to-end distance probability distributions of MD-generated hyaluronan 20-mer conformations with β1-3 linkages with -ϕ, +ψ dihedrals in each of the four runs; as the end-to-end distance distribution from run 2 appeared to be an outlier, these data were compared to the average end-to-end distance distribution of all snapshots in MD runs 1, 3, and 4 (black dashed line).

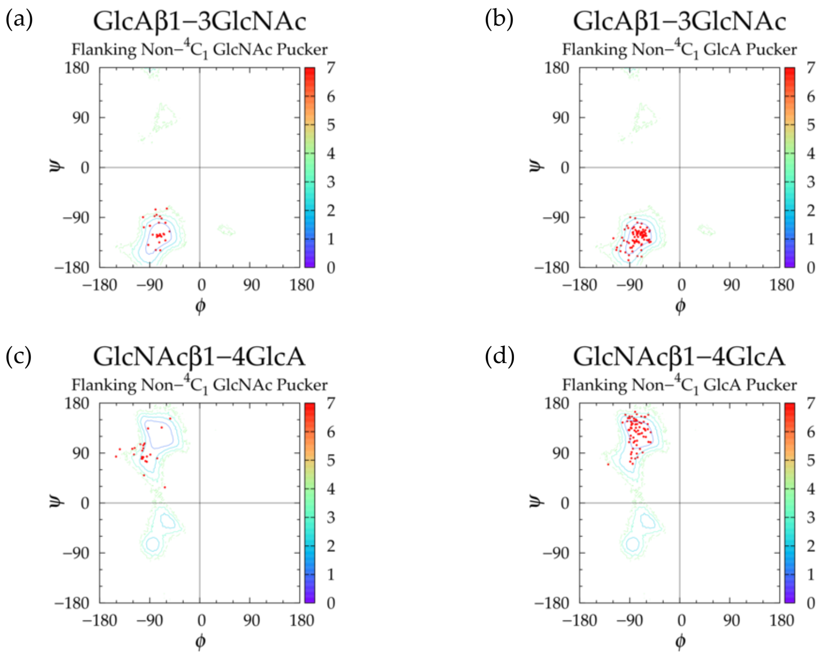

Figure S9. Scatterplots for dihedrals ϕ and ψ of glycosidic linkages flanking non-$^4C_1$ ring puckers in the MD-generated hyaluronan 20-mer ensemble: (a) GlcAβ1-3GlcNAc flanking GlcNAc, (b) GlcAβ1-3GlcNAc flanking GlcA, (c) GlcNAcβ1-4GlcA flanking GlcNAc, (d) GlcNAcβ1-4GlcA flanking GlcA; contour lines come from corresponding aggregated MD-generated $\Delta G(\phi,\psi)$ data.

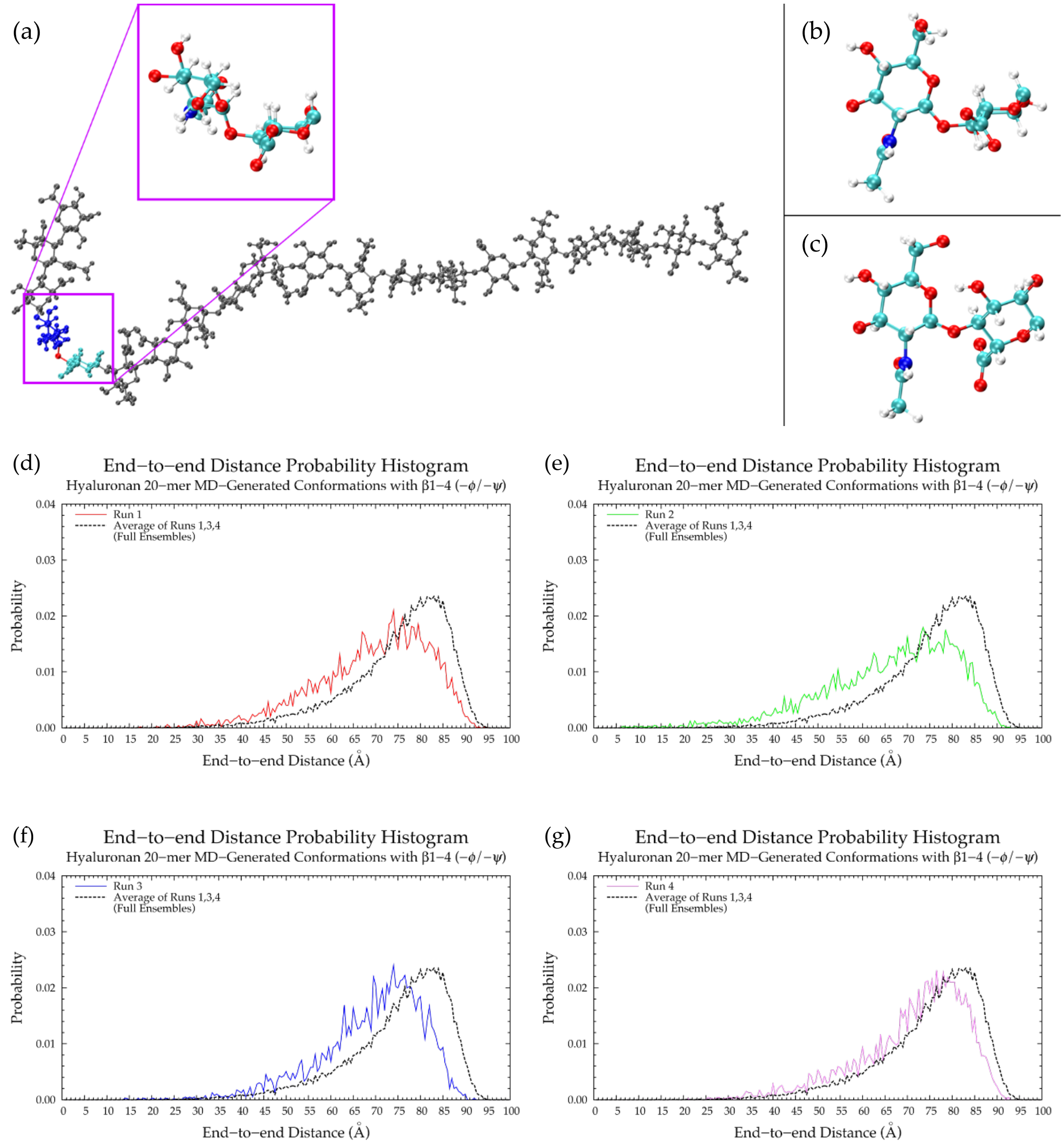

Figure S10. Snapshots from hyaluronan 20-mer MD: (a) 20-mer conformation with GlcNAc 17 (blue) β1-4 GlcA 16 (cyan) linkage dihedrals near $\Delta G(\phi, \psi)$ min II (ϕ = -86.8° and ψ = -78.6°), which causes a kink (linker oxygen is red), and closeup of this disaccharide unit, (b) closeup of this disaccharide unit with linkage dihedrals near $\Delta G(\phi, \psi)$ min II′ (ϕ = -55.7° and ψ = -39.7°), (c) closeup of the same disaccharide unit with linkage dihedrals near $\Delta G(\phi, \psi)$ min I (ϕ = -68.3° and ψ = +115.2°). (d-g) End-to-end distance probability distributions of MD-generated hyaluronan 20-mer conformations with β1-4 linkages with -ϕ, -ψ dihedrals in each of the four runs; as the end-to-end distance distribution from run 2 appeared to be an outlier, these data were compared to the average end-to-end distance distribution of all snapshots in MD runs 1, 3, and 4 (black dashed line).

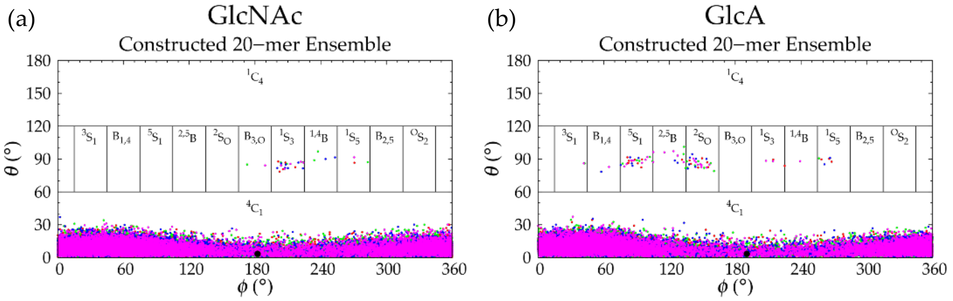

Figure S11. Cremer–Pople data for (a) GlcNAc and (b) GlcA in the constructed hyaluronan 20-mer ensemble; each of the 4 runs is represented by different color and the force-field geometry is represented by a single large black dot; each run contains 10,000 parameter sets.

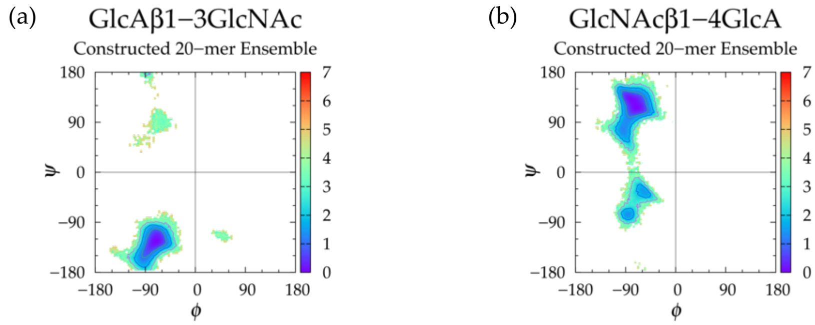

Figure S12. $\Delta G(\phi,\ \psi)$ in the constructed hyaluronan 20-mer ensemble for aggregated (a) GlcAβ1-3GlcNAc and (b) GlcNAcβ1-4GlcA glycosidic linkage data; contour lines every 1 kcal/mol.

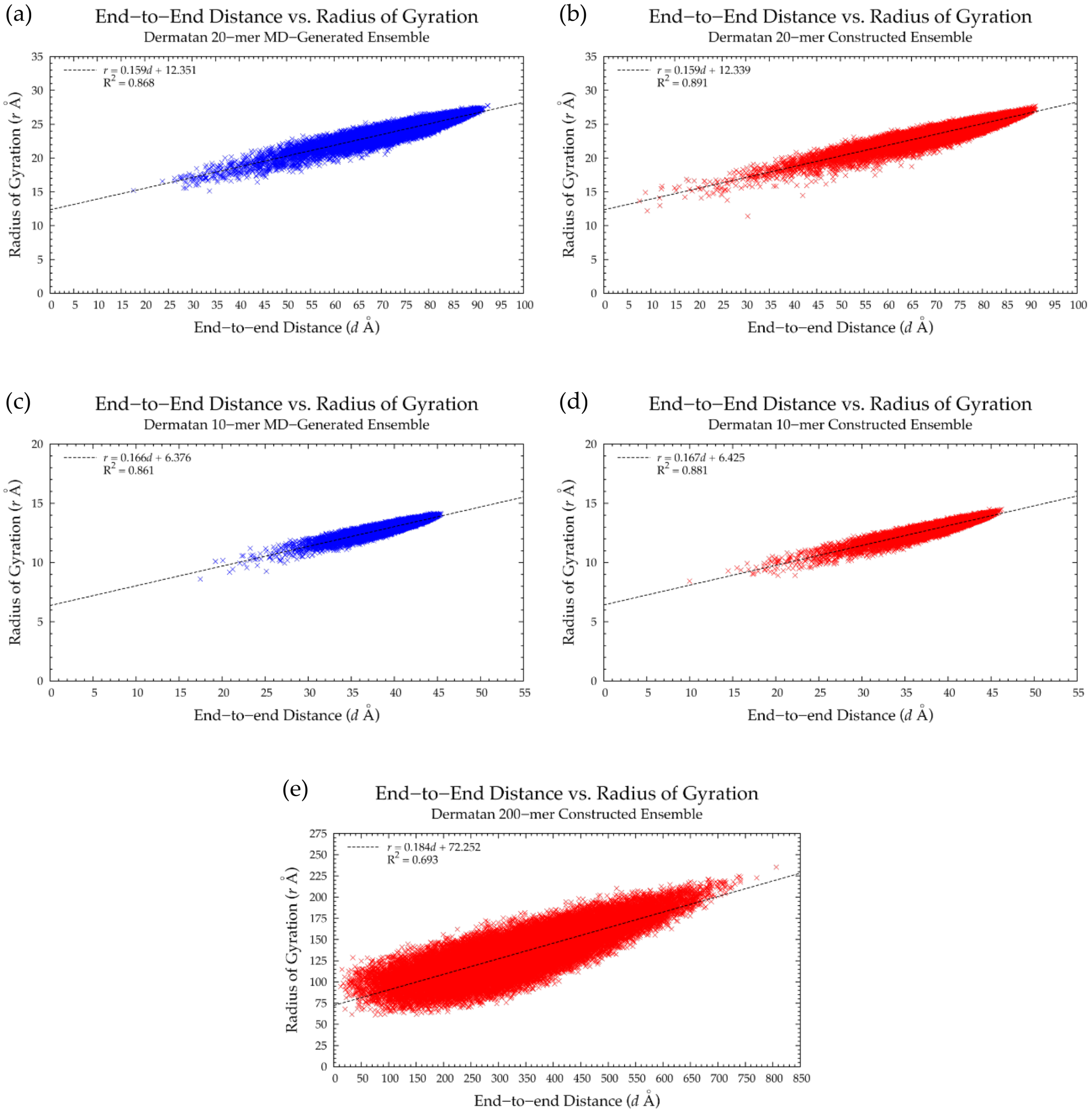

Figure S13. Scatterplots of radius of gyration as a function of end-to-end distance in MD-generated and constructed ensembles of non-sulfated dermatan (a,b) 20-mer and (c,d) 10-mer, respectively, and (e) constructed ensemble of non-sulfated dermatan 200-mer; each plot has 40,000 samples and shows linear regression and R^2.

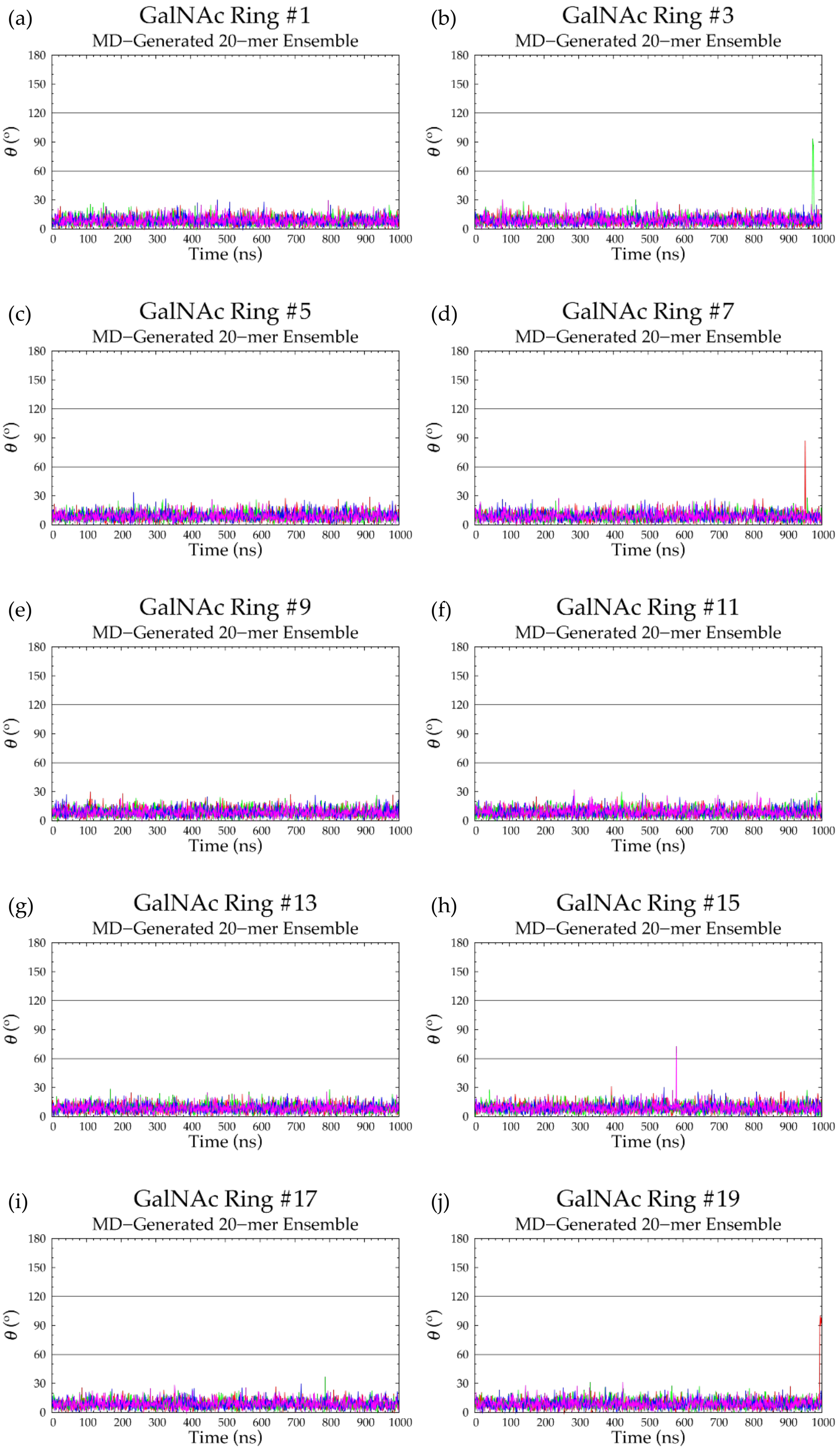

(a)
GalNAc Ring #1
MD−Generated 20−mer Ensemble
(b)
GalNAc Ring #3
MD−Generated 20−mer Ensemble
(c)
GalNAc Ring #5
MD−Generated 20−mer Ensemble
(d)
GalNAc Ring #7
MD−Generated 20−mer Ensemble
(e)
GalNAc Ring #9
MD−Generated 20−mer Ensemble
(f)
GalNAc Ring #11
MD−Generated 20−mer Ensemble
(g)
GalNAc Ring #13
MD−Generated 20−mer Ensemble
(h)
GalNAc Ring #15
MD−Generated 20−mer Ensemble
(i)
GalNAc Ring #17
MD−Generated 20−mer Ensemble
(j)
GalNAc Ring #19
MD−Generated 20−mer Ensemble
θ (°)
Time (ns)

Figure S14. (a-j) Cremer-Pople parameter θ timeseries for each GalNAc monosaccharide ring in the MD-generated non-sulfated dermatan 20-mer ensemble; monosaccharides are numbered from reducing to non-reducing end; each of the 4 runs is represented by different color.

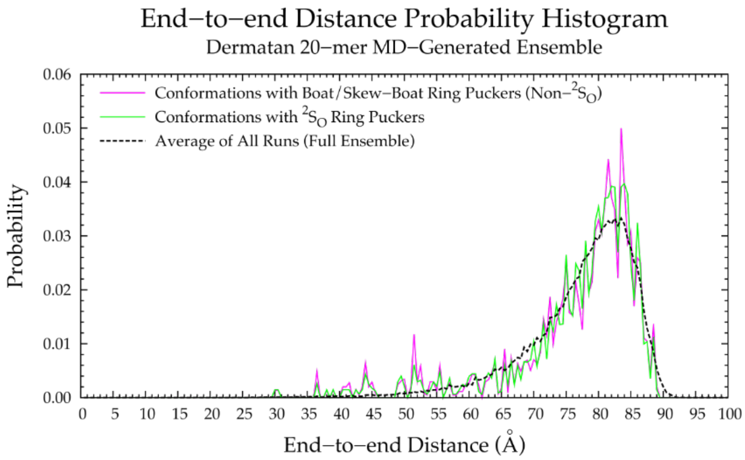

Figure S15. End-to-end distance distributions of MD-generated non-sulfated dermatan 20-mer conformations with boat/skew-boat ring puckers that cause a kink in the polymer chain, i.e. non-^2S_O (pink solid line; most probable end-to-end distance is 83.5 Å) and ^2S_O conformations (green solid line; most probable end-to-end distance is 84.0 Å) and the average of all four runs in the full MD-generated ensemble (black dashed line; most probable end-to-end distance is 83.5 Å); probabilities were calculated for end-to-end distances sorted into 0.5 Å bins.

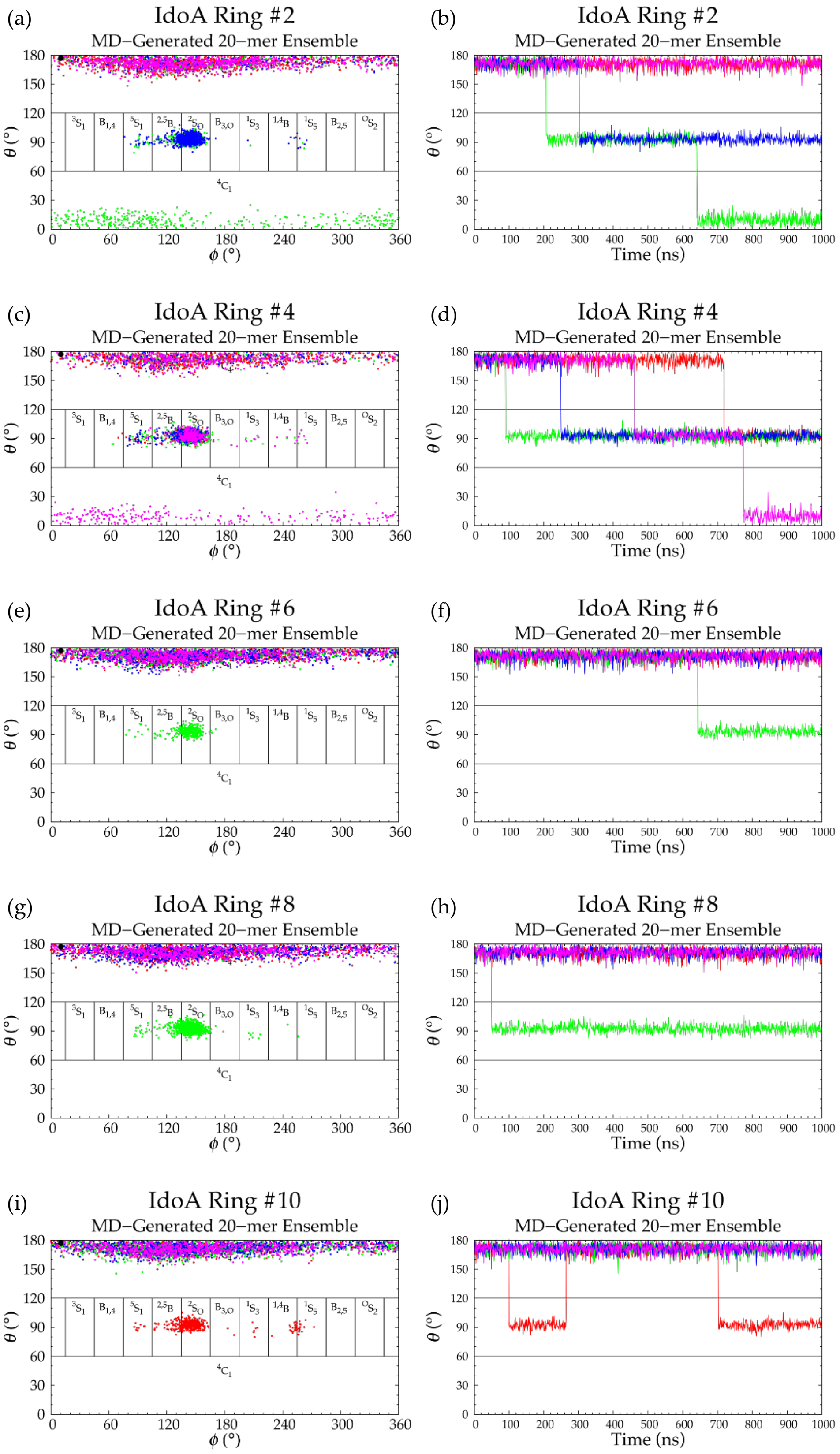

(a)
IdoA Ring #2
MD−Generated 20−mer Ensemble
(b)
IdoA Ring #2
MD−Generated 20−mer Ensemble
(c)
IdoA Ring #4
MD−Generated 20−mer Ensemble
(d)
IdoA Ring #4
MD−Generated 20−mer Ensemble
(e)
IdoA Ring #6
MD−Generated 20−mer Ensemble
(f)
IdoA Ring #6
MD−Generated 20−mer Ensemble
(g)
IdoA Ring #8
MD−Generated 20−mer Ensemble
(h)
IdoA Ring #8
MD−Generated 20−mer Ensemble
(i)
IdoA Ring #10
MD−Generated 20−mer Ensemble
(j)
IdoA Ring #10
MD−Generated 20−mer Ensemble
θ (°)
ϕ (°)
Time (ns)
$^{3}S_{1}$ $B_{1,4}$ $^{5}S_{1}$ $^{2,5}B$ $^{2}S_{O}$ $B_{3,O}$ $^{1}S_{3}$ $^{1,4}B$ $^{1}S_{5}$ $B_{2,5}$ $^{O}S_{2}$
$^{4}C_{1}$

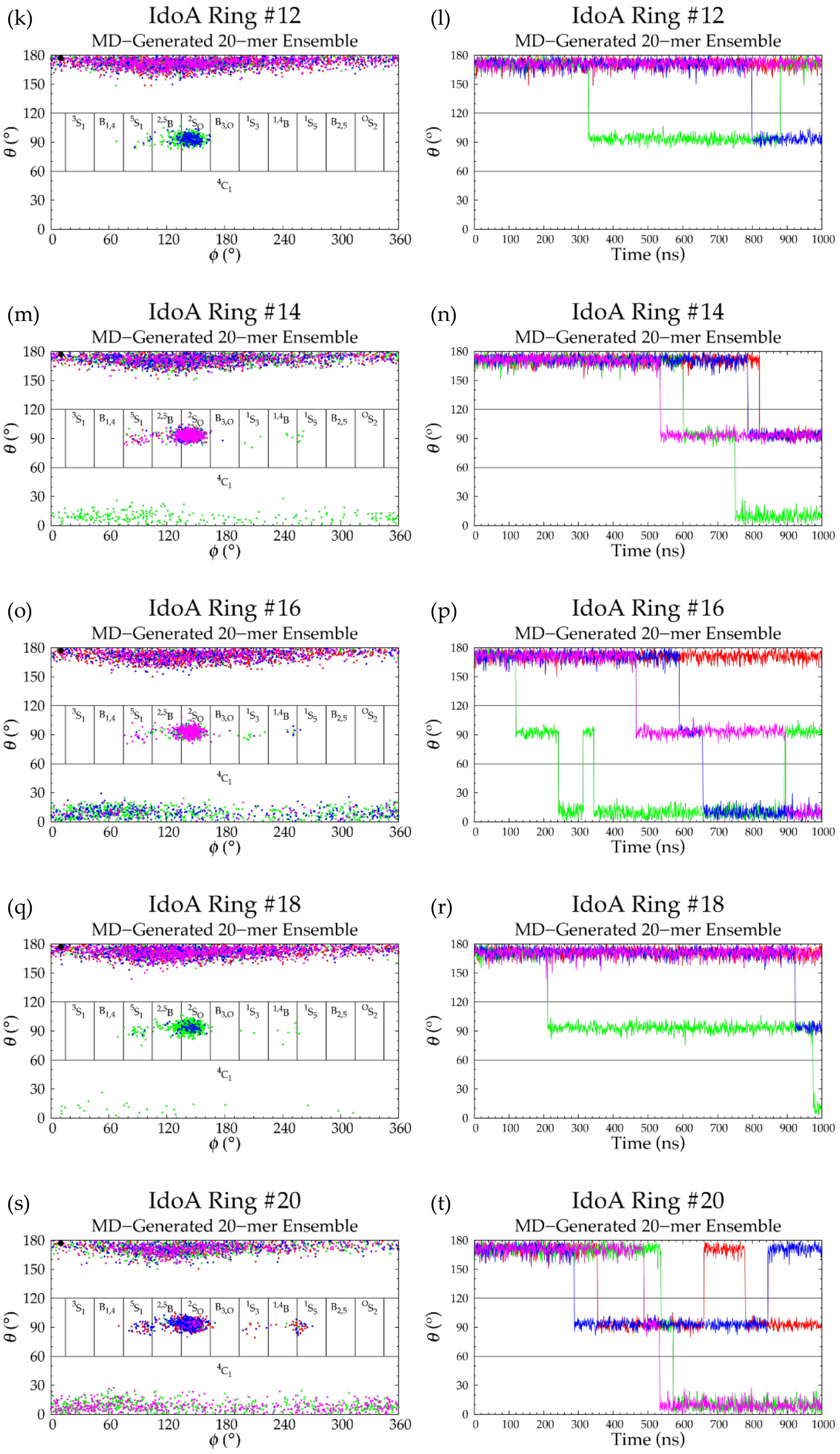
(k)
IdoA Ring #12
MD−Generated 20−mer Ensemble
θ (°)
ϕ (°)
$^3S_1$ $B_{1,4}$ $^5S_1$ $^{2,5}B$ ^2S_O $B_{3,O}$ $^1S_3$ $^{1,4}B$ $^1S_5$ $B_{2,5}$ $^OS_2$
$^4C_1$
(l)
IdoA Ring #12
MD−Generated 20−mer Ensemble
θ (°)
Time (ns)
(m)
IdoA Ring #14
MD−Generated 20−mer Ensemble
θ (°)
ϕ (°)
(n)
IdoA Ring #14
MD−Generated 20−mer Ensemble
θ (°)
Time (ns)
(o)
IdoA Ring #16
MD−Generated 20−mer Ensemble
θ (°)
ϕ (°)
(p)
IdoA Ring #16
MD−Generated 20−mer Ensemble
θ (°)
Time (ns)
(q)
IdoA Ring #18
MD−Generated 20−mer Ensemble
θ (°)
ϕ (°)
(r)
IdoA Ring #18
MD−Generated 20−mer Ensemble
θ (°)
Time (ns)
(s)
IdoA Ring #20
MD−Generated 20−mer Ensemble
θ (°)
ϕ (°)
(t)
IdoA Ring #20
MD−Generated 20−mer Ensemble
θ (°)
Time (ns)

Figure S16. (a,c,e,g,i,k,m,o,q,s) Cremer-Pople plots and (b,d,f,h,j,l,n,p,r,t) Cremer-Pople parameter θ timeseries for each IdoA monosaccharide ring in the MD-generated non-sulfated dermatan 20-mer ensemble; monosaccharides are numbered from reducing to non-reducing end; each of the 4 runs is represented by different color.

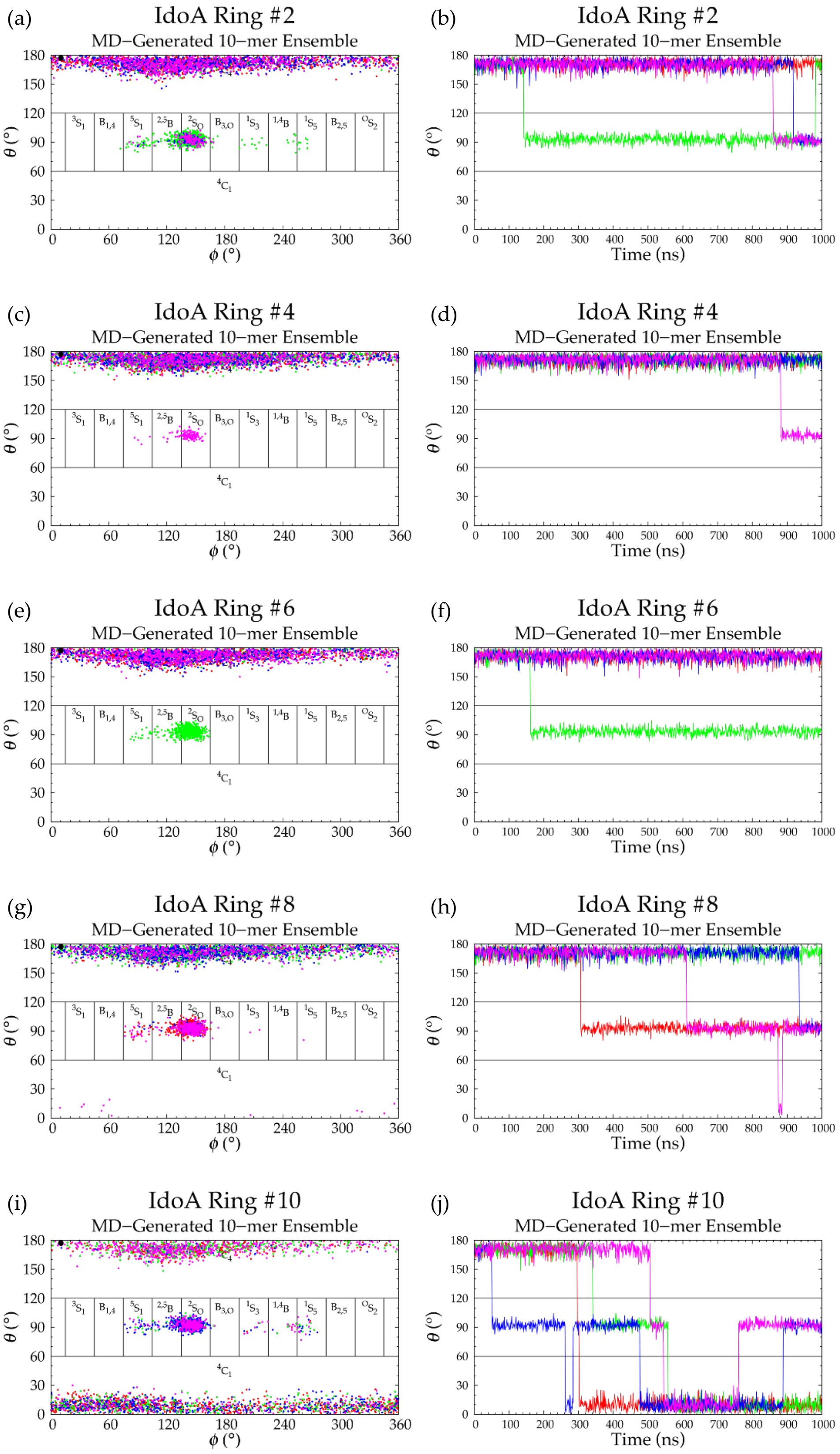

(a)
IdoA Ring #2
MD−Generated 10−mer Ensemble
(b)
IdoA Ring #2
MD−Generated 10−mer Ensemble
(c)
IdoA Ring #4
MD−Generated 10−mer Ensemble
(d)
IdoA Ring #4
MD−Generated 10−mer Ensemble
(e)
IdoA Ring #6
MD−Generated 10−mer Ensemble
(f)
IdoA Ring #6
MD−Generated 10−mer Ensemble
(g)
IdoA Ring #8
MD−Generated 10−mer Ensemble
(h)
IdoA Ring #8
MD−Generated 10−mer Ensemble
(i)
IdoA Ring #10
MD−Generated 10−mer Ensemble
(j)
IdoA Ring #10
MD−Generated 10−mer Ensemble
θ (°)
ϕ (°)
Time (ns)
$^3S_1$ $B_{1,4}$ $^5S_1$ $^{2,5}B$ ^2S_O $B_{3,O}$ $^1S_3$ $^{1,4}B$ $^1S_5$ $B_{2,5}$ $^OS_2$
$^4C_1$

Figure S17. (a,c,e,g,i) Cremer-Pople plots and (b,d,f,h,j) Cremer-Pople parameter θ timeseries for each IdoA monosaccharide ring in the MD-generated non-sulfated dermatan 10-mer ensemble; monosaccharides are numbered from reducing to non-reducing end; each of the 4 runs is represented by different color.

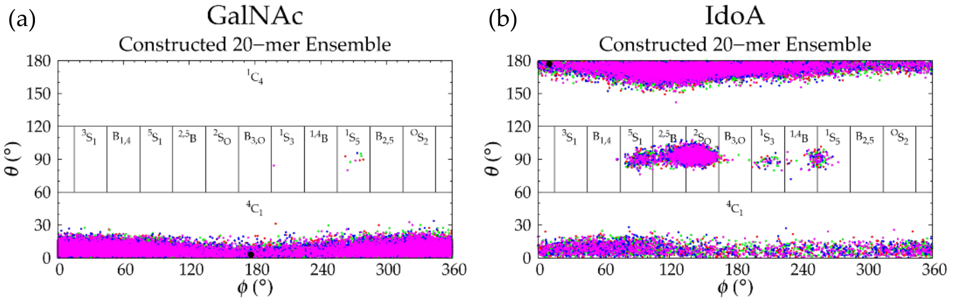

Figure S18. Cremer–Pople data for (a) GalNAc and (b) IdoA in the constructed non-sulfated dermatan 20-mer ensemble; each of the 4 runs is represented by different color and the force-field geometry is represented by a single large black dot; each run contains 10,000 parameter sets.

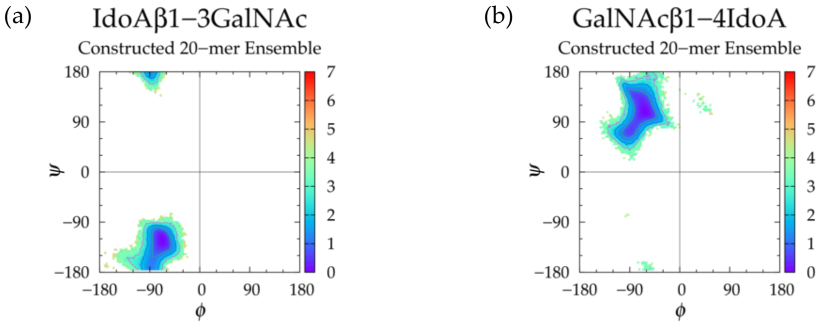

Figure S19. $\Delta G(\phi,\ \psi)$ in the constructed non-sulfated dermatan 20-mer ensemble for aggregated (a) IdoAβ1-3GalNAc and (b) GalNAcβ1-4IdoA glycosidic linkage data; contour lines every 1 kcal/mol.

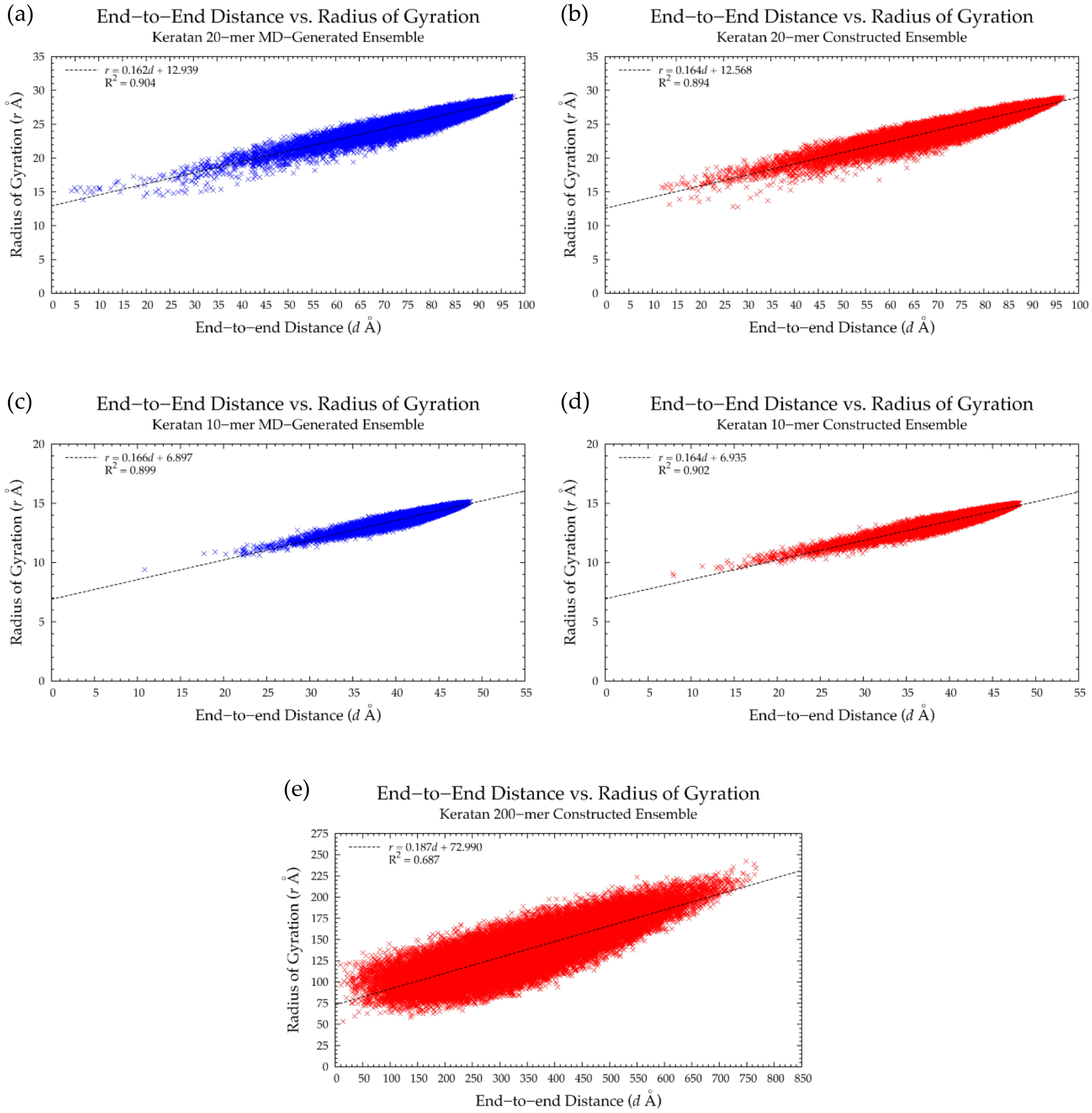

Figure S20. Scatterplots of radius of gyration as a function of end-to-end distance in MD-generated and constructed ensembles of non-sulfated keratan (a,b) 20-mer and (c,d) 10-mer, respectively, and (e) constructed ensemble of non-sulfated keratan 200-mer; each plot has 40,000 samples and shows linear regression and R^2.

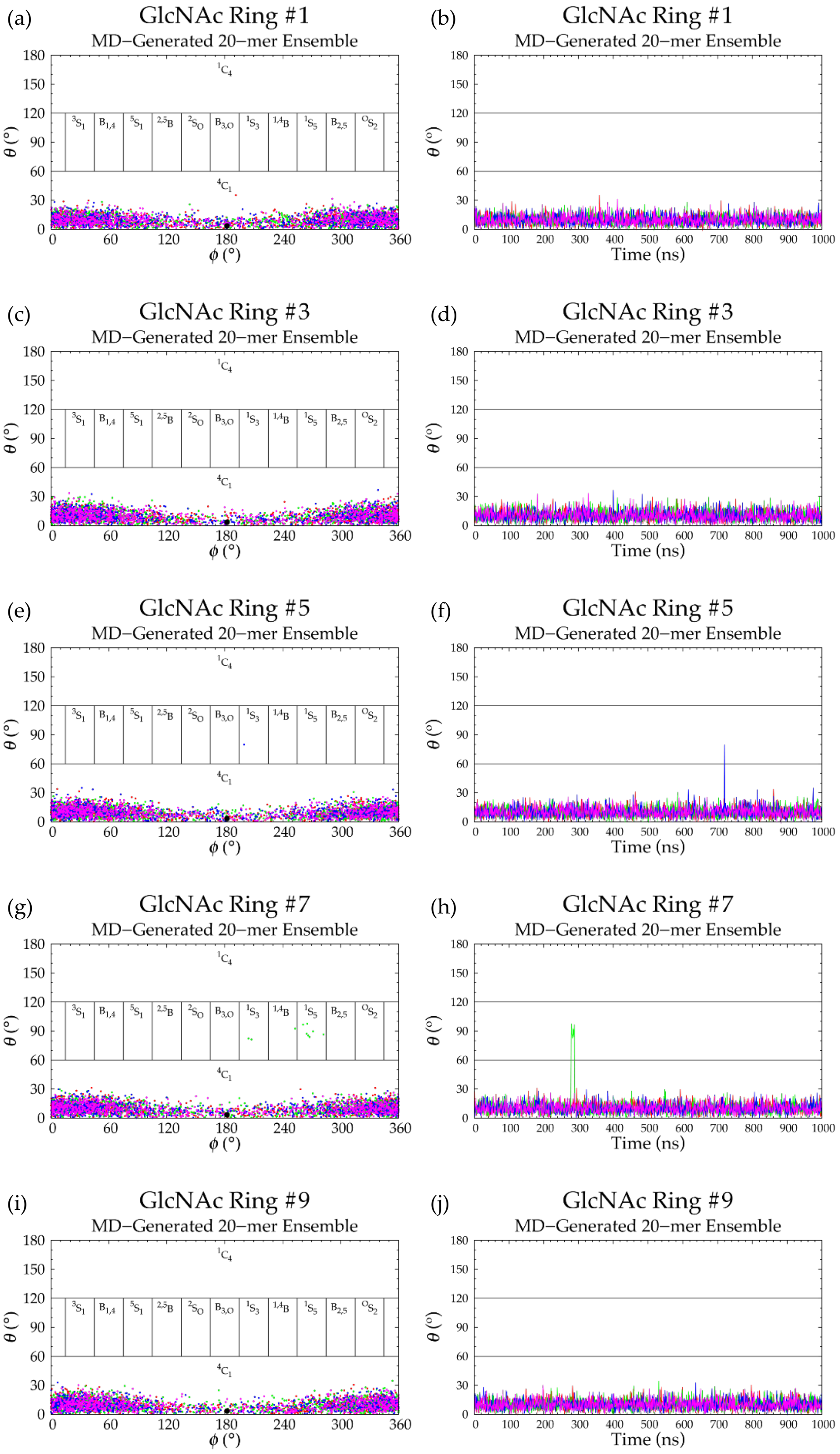

(a)
GlcNAc Ring #1
MD−Generated 20−mer Ensemble
(b)
GlcNAc Ring #1
MD−Generated 20−mer Ensemble
(c)
GlcNAc Ring #3
MD−Generated 20−mer Ensemble
(d)
GlcNAc Ring #3
MD−Generated 20−mer Ensemble
(e)
GlcNAc Ring #5
MD−Generated 20−mer Ensemble
(f)
GlcNAc Ring #5
MD−Generated 20−mer Ensemble
(g)
GlcNAc Ring #7
MD−Generated 20−mer Ensemble
(h)
GlcNAc Ring #7
MD−Generated 20−mer Ensemble
(i)
GlcNAc Ring #9
MD−Generated 20−mer Ensemble
(j)
GlcNAc Ring #9
MD−Generated 20−mer Ensemble
$^1C_4$
$^3S_1$ $B_{1,4}$ $^5S_1$ $^{2,5}B$ ^2S_O $B_{3,O}$ $^1S_3$ $^{1,4}B$ $^1S_5$ $B_{2,5}$ $^OS_2$
$^4C_1$
θ (°)
ϕ (°)
Time (ns)

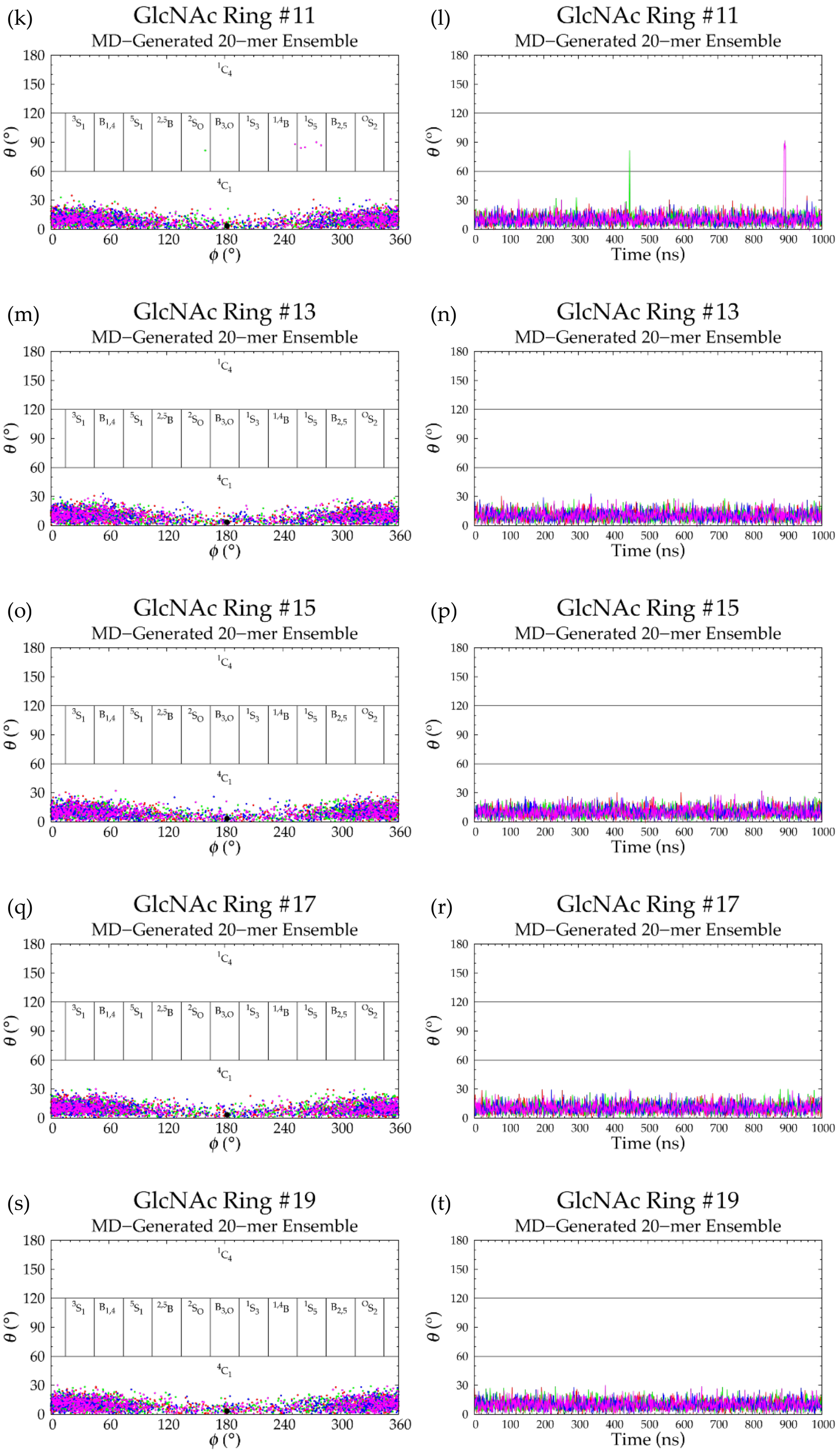
(k)
GlcNAc Ring #11
MD−Generated 20−mer Ensemble
(l)
GlcNAc Ring #11
MD−Generated 20−mer Ensemble
(m)
GlcNAc Ring #13
MD−Generated 20−mer Ensemble
(n)
GlcNAc Ring #13
MD−Generated 20−mer Ensemble
(o)
GlcNAc Ring #15
MD−Generated 20−mer Ensemble
(p)
GlcNAc Ring #15
MD−Generated 20−mer Ensemble
(q)
GlcNAc Ring #17
MD−Generated 20−mer Ensemble
(r)
GlcNAc Ring #17
MD−Generated 20−mer Ensemble
(s)
GlcNAc Ring #19
MD−Generated 20−mer Ensemble
(t)
GlcNAc Ring #19
MD−Generated 20−mer Ensemble
θ (°)
ϕ (°)
Time (ns)
$^{1}C_{4}$
$^{4}C_{1}$
$^{3}S_{1}$
$B_{1,4}$
$^{5}S_{1}$
$^{2,5}B$
$^{2}S_{O}$
$B_{3,O}$
$^{1}S_{3}$
$^{1,4}B$
$^{1}S_{5}$
$B_{2,5}$
$^{O}S_{2}$

Figure S21. (a,c,e,g,i,k,m,o,q,s) Cremer-Pople plots and (b,d,f,h,j,l,n,p,r,t) Cremer-Pople parameter θ timeseries for each GlcNAc monosaccharide ring in the MD-generated non-sulfated keratan 20-mer ensemble; monosaccharides are numbered from reducing to non-reducing end; each of the 4 runs is represented by different color.

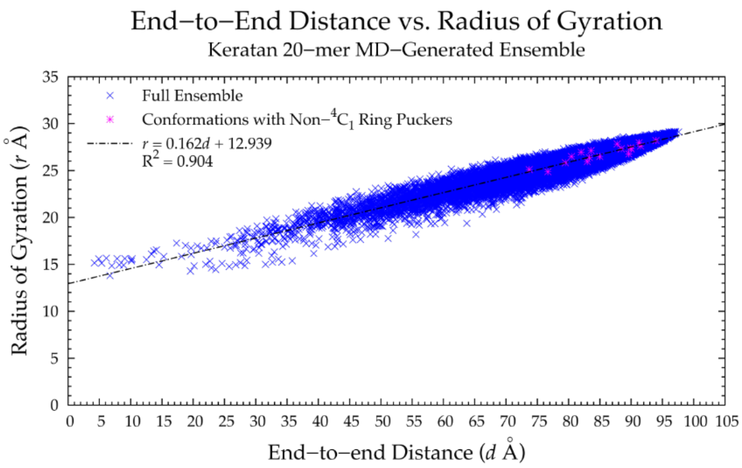

Figure S22. Scatterplot of radius of gyration as a function of end-to-end distance in MD-generated non-sulfated keratan 20-mer conformations with non-$^4C_1$ ring puckers overlaid on data for full MD-generated non-sulfated keratan 20-mer ensemble and corresponding linear regression (*Figure S34a*).

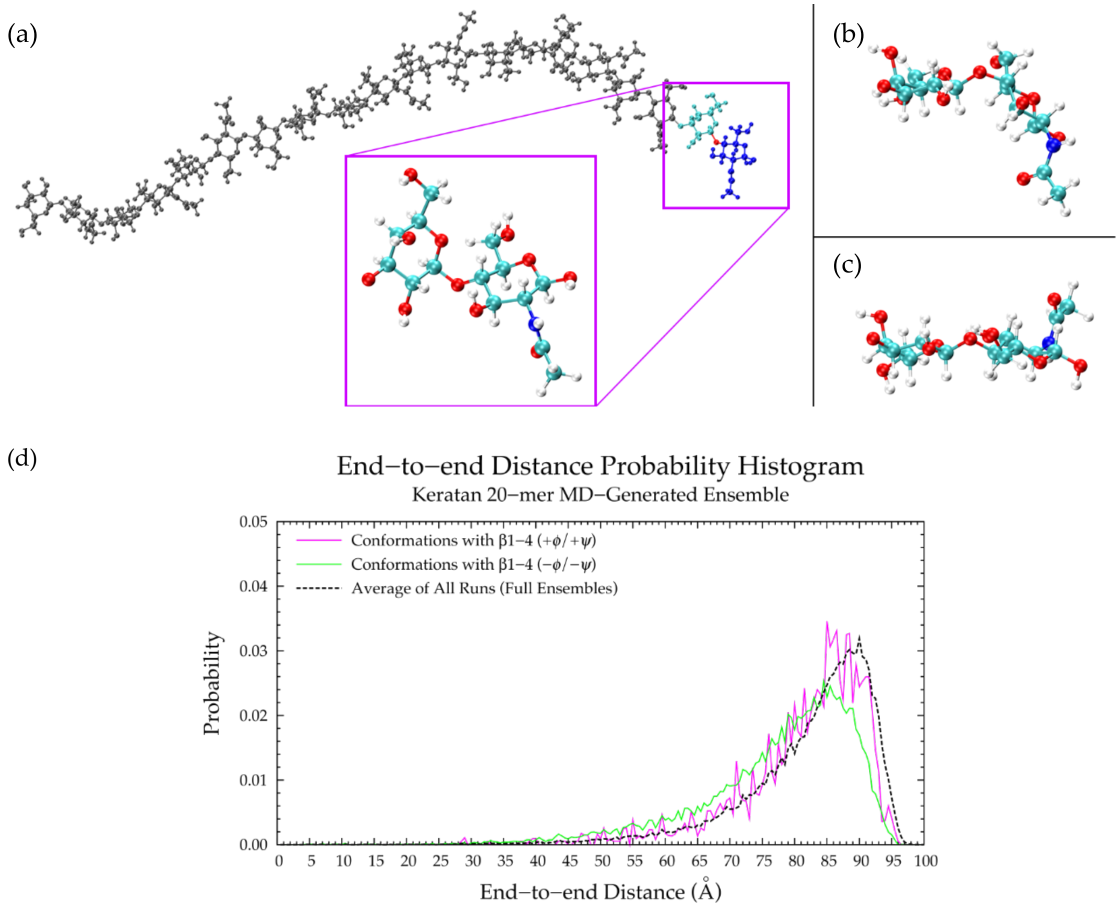

Figure S23. Snapshots from non-sulfated keratan 20-mer MD: (a) 20-mer conformation with Gal 2 (cyan) β1-4 GlcNAc 1 (blue) linkage dihedrals in tertiary basin (ϕ = +13.8° and ψ = +131.1°), which causes a slight bend (linker oxygen is red), and closeup of this disaccharide unit, (b) closeup of this disaccharide unit with linkage dihedrals in secondary basin, i.e. near $\Delta G(\phi, \psi)$ min II (ϕ = -98.8° and ψ = -71.0°), (c) closeup of the same disaccharide unit with linkage dihedrals in primary basin near $\Delta G(\phi, \psi)$ min I (ϕ = -86.2° and ψ = +100.5°). (d) End-to-end distance probability distributions of MD-generated non-sulfated keratan 20-mer conformations with β1-4 linkages with +ϕ, +ψ (pink solid line; most probable end-to-end distance is 85.0 Å) and -ϕ, -ψ (green solid line; most probable end-to-end distance is 84.5 Å) dihedrals in all four runs; these data were compared to the average end-to-end distance distribution of all snapshots in all four MD runs (black dashed line; most probable end-to-end distance is 90.0 Å).

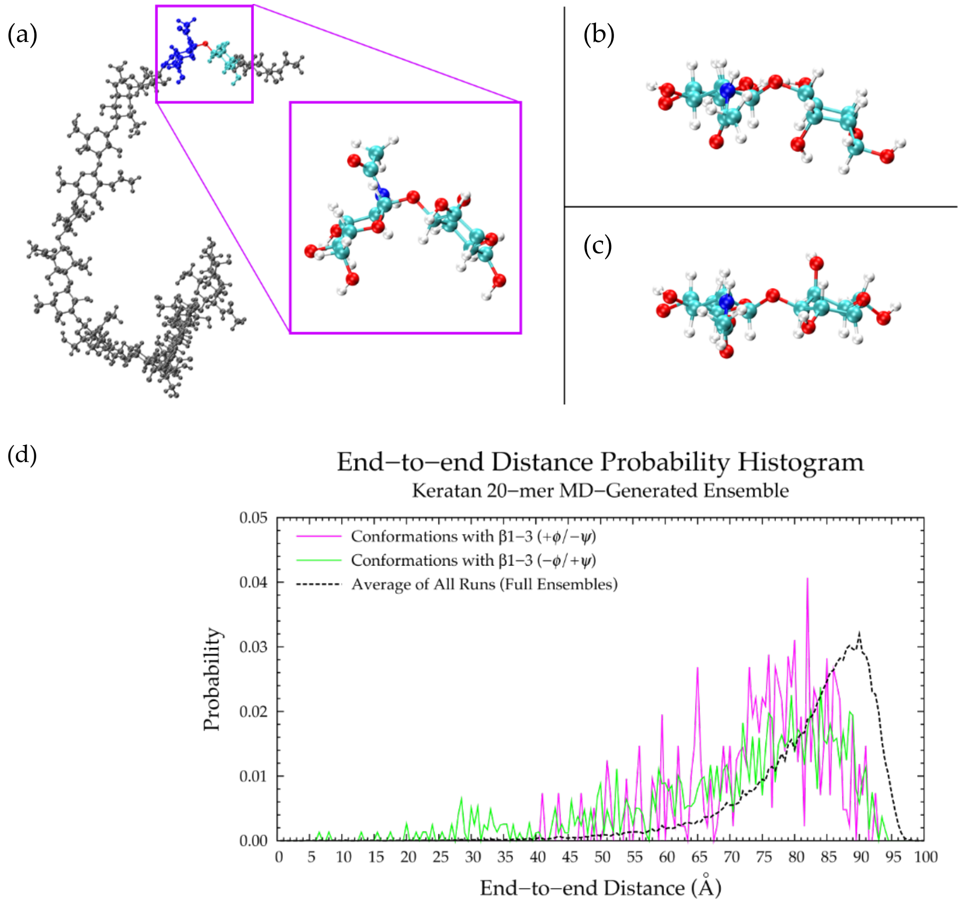

Figure S24. Snapshots from non-sulfated keratan 20-mer MD: (a) 20-mer conformation with GlcNAc 3 (blue) β1-3 Gal 2 (cyan) linkage dihedrals in tertiary basin (ϕ = +38.8° and ψ = -127.3°), which causes a kink (linker oxygen is red), and closeup of this disaccharide unit, (b) closeup of GlcNAc 5 (blue) β1-3 Gal 4 (cyan) disaccharide unit with linkage dihedrals in secondary basin (ϕ = -95.3° and ψ = +39.9°), (c) closeup of the same disaccharide unit with linkage dihedrals in primary basin (ϕ = -80.7° and ψ = -138.2°). (d) End-to-end distance probability distributions of MD-generated non-sulfated keratan 20-mer conformations with β1-3 linkages with +ϕ, -ψ (pink solid line; most probable end-to-end distance is 82.0 Å) and -ϕ, +ψ (green solid line; most probable end-to-end distance is 84.0 Å) dihedrals in all four runs; these data were compared to the average end-to-end distance distribution of all snapshots in all four MD runs (black dashed line; most probable end-to-end distance is 90.0 Å).

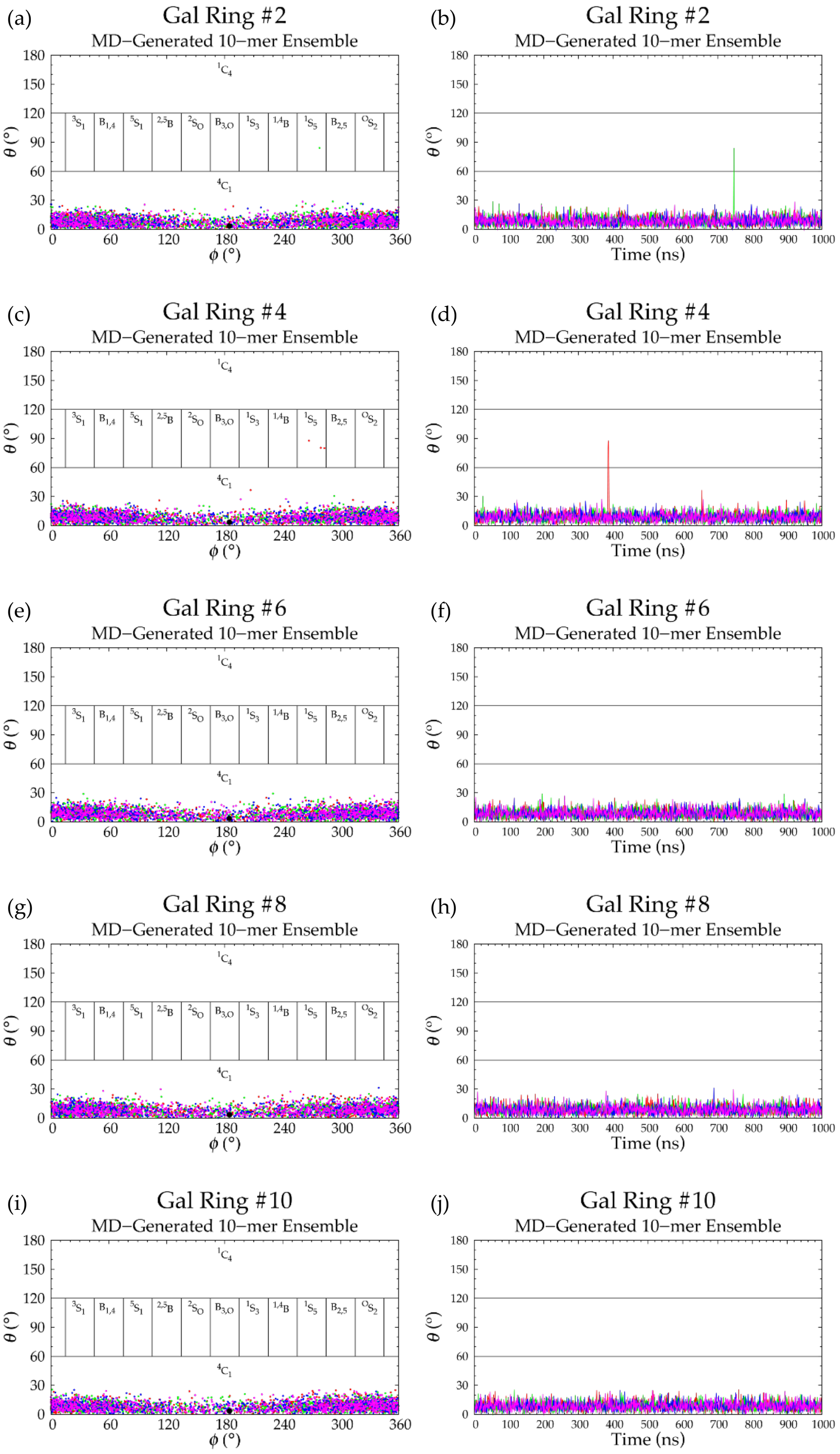

(a)
Gal Ring #2
MD−Generated 10−mer Ensemble
(b)
Gal Ring #2
MD−Generated 10−mer Ensemble
(c)
Gal Ring #4
MD−Generated 10−mer Ensemble
(d)
Gal Ring #4
MD−Generated 10−mer Ensemble
(e)
Gal Ring #6
MD−Generated 10−mer Ensemble
(f)
Gal Ring #6
MD−Generated 10−mer Ensemble
(g)
Gal Ring #8
MD−Generated 10−mer Ensemble
(h)
Gal Ring #8
MD−Generated 10−mer Ensemble
(i)
Gal Ring #10
MD−Generated 10−mer Ensemble
(j)
Gal Ring #10
MD−Generated 10−mer Ensemble
$^1C_4$
$^3S_1$ $B_{1,4}$ $^5S_1$ $^{2,5}B$ ^2S_O $B_{3,O}$ $^1S_3$ $^{1,4}B$ $^1S_5$ $B_{2,5}$ $^OS_2$
$^4C_1$
θ (°)
ϕ (°)
Time (ns)
0 30 60 90 120 150 180
0 60 120 180 240 300 360
0 100 200 300 400 500 600 700 800 900 1000

Figure S25. (a,c,e,g,i) Cremer-Pople plots and (b,d,f,h,j) Cremer-Pople parameter θ timeseries for each Gal monosaccharide ring in the MD-generated non-sulfated keratan 10-mer ensemble; monosaccharides are numbered from reducing to non-reducing end; each of the 4 runs is represented by different color.

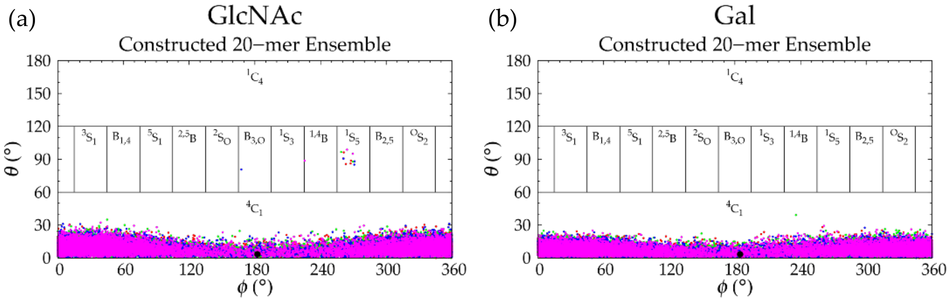

Figure S26. Cremer–Pople data for (a) GlcNAc and (b) Gal in the constructed non-sulfated keratan 20-mer ensemble; each of the 4 runs is represented by different color and the force-field geometry is represented by a single large black dot; each run contains 10,000 parameter sets.

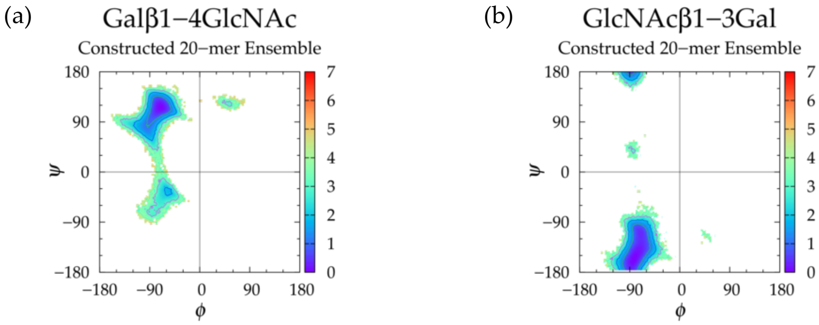

Figure S27. $\Delta G(\phi,\ \psi)$ in the constructed non-sulfated keratan 20-mer ensemble for aggregated (a) Galβ1-4GlcNAc and (b) GlcNAcβ1-3Gal glycosidic linkage data; contour lines every 1 kcal/mol.

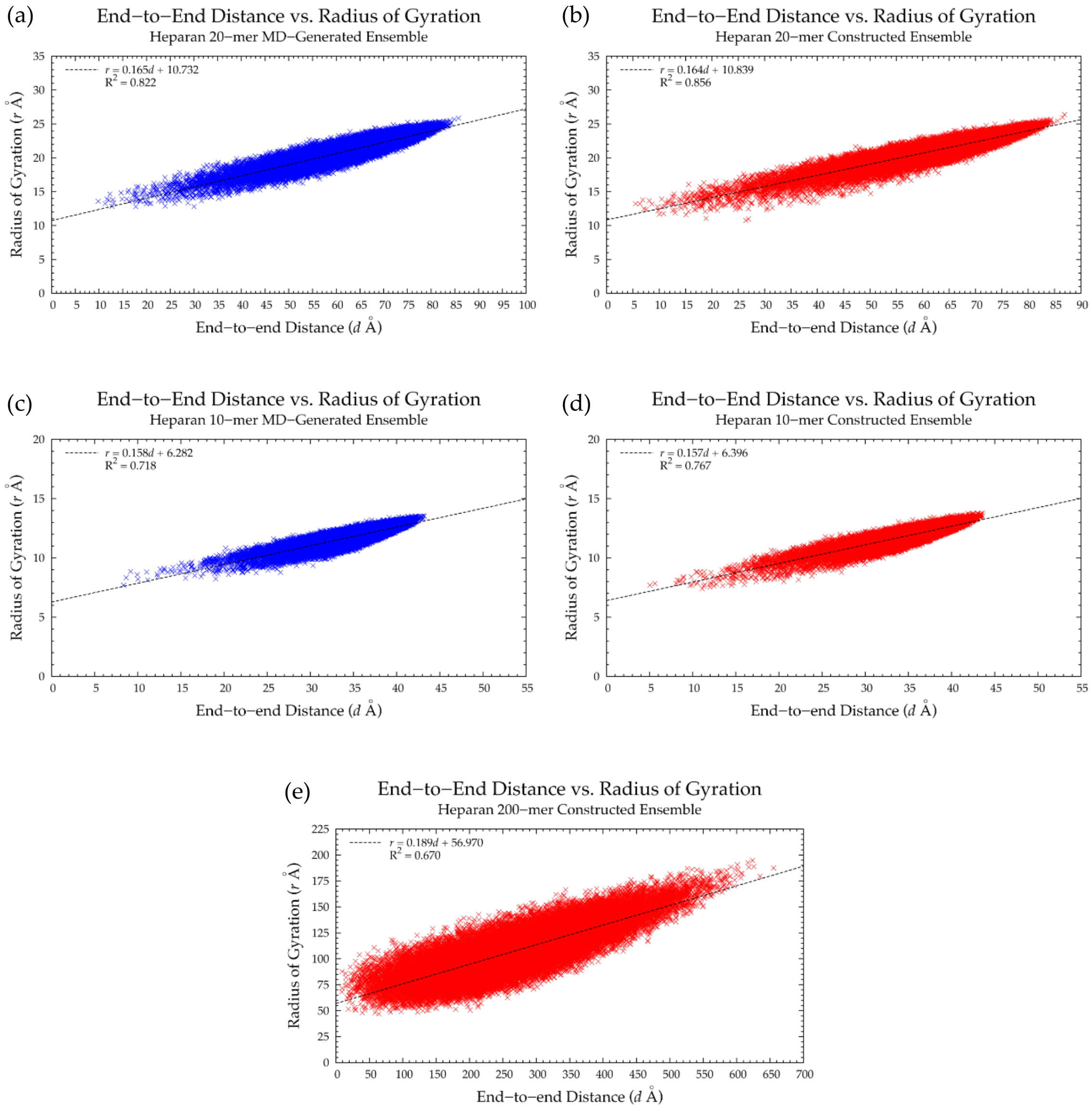

Figure S28. Scatterplots of radius of gyration as a function of end-to-end distance in MD-generated and constructed ensembles of non-sulfated heparan (a,b) 20-mer and (c,d) 10-mer, respectively, and (e) constructed ensemble of non-sulfated heparan 200-mer; each plot has 40,000 samples and shows linear regression and R^2.

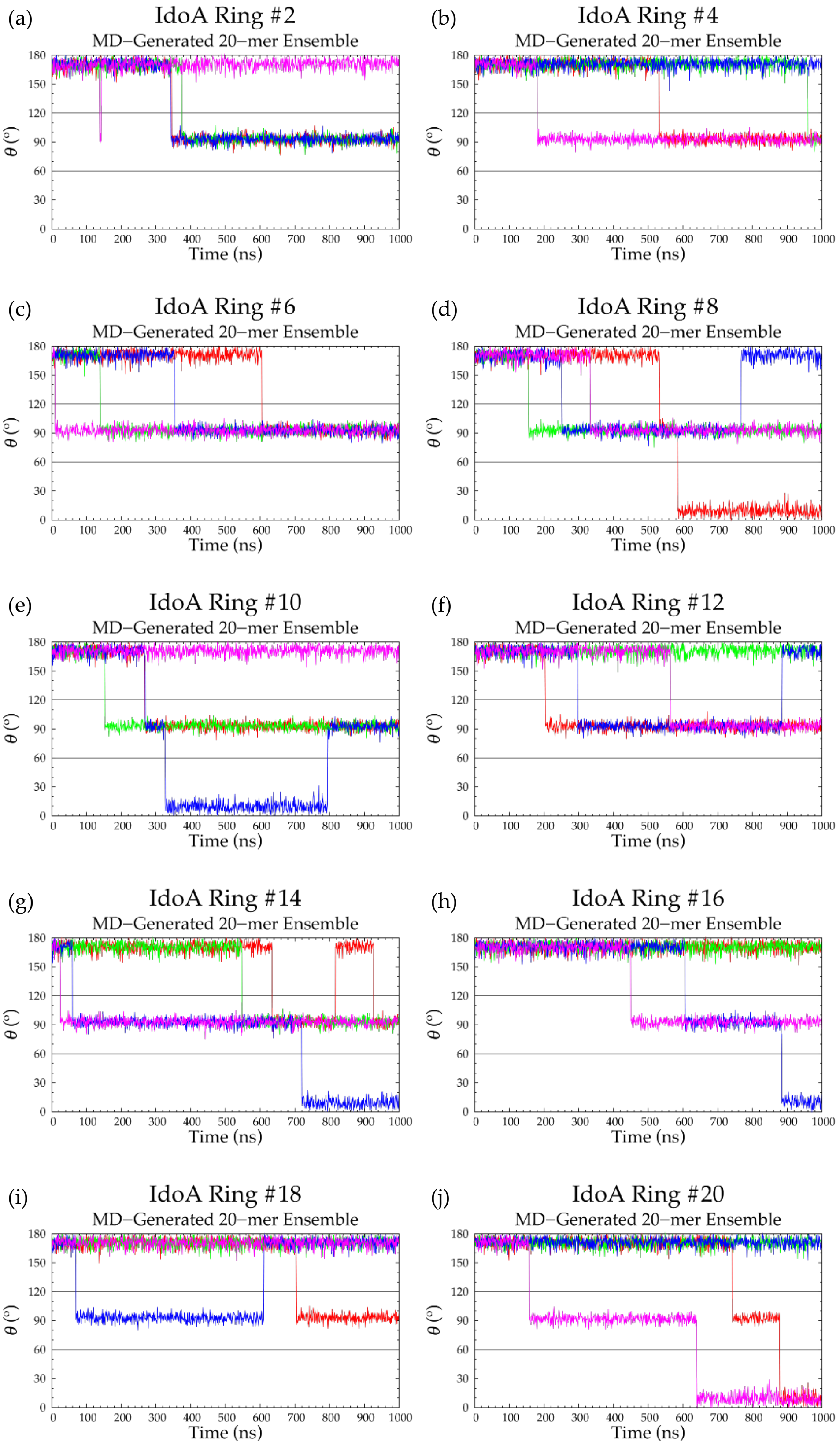
(a)
IdoA Ring #2
MD−Generated 20−mer Ensemble
(b)
IdoA Ring #4
MD−Generated 20−mer Ensemble
(c)
IdoA Ring #6
MD−Generated 20−mer Ensemble
(d)
IdoA Ring #8
MD−Generated 20−mer Ensemble
(e)
IdoA Ring #10
MD−Generated 20−mer Ensemble
(f)
IdoA Ring #12
MD−Generated 20−mer Ensemble
(g)
IdoA Ring #14
MD−Generated 20−mer Ensemble
(h)
IdoA Ring #16
MD−Generated 20−mer Ensemble
(i)
IdoA Ring #18
MD−Generated 20−mer Ensemble
(j)
IdoA Ring #20
MD−Generated 20−mer Ensemble
θ (°)
0 30 60 90 120 150 180
Time (ns)
0 100 200 300 400 500 600 700 800 900 1000

Figure S29. (a-j) Cremer-Pople parameter θ timeseries for each IdoA monosaccharide ring in the MD-generated non-sulfated heparan 20-mer ensemble; monosaccharides are numbered from reducing to non-reducing end; each of the 4 runs is represented by different color.

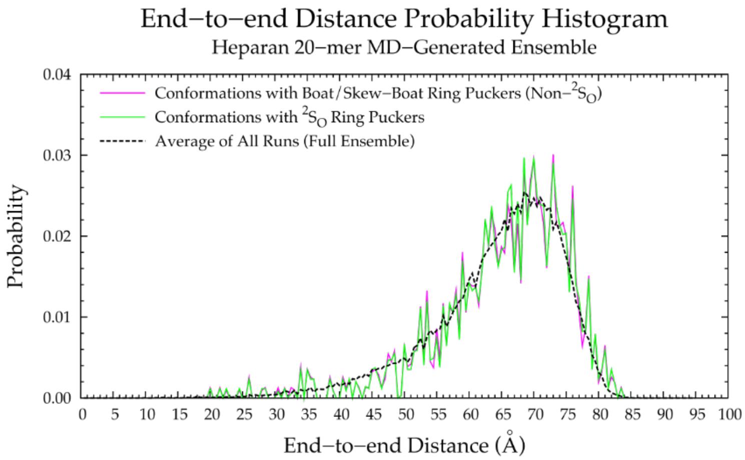

Figure S30. End-to-end distance distributions of MD-generated non-sulfated heparan 20-mer conformations with boat/skew-boat ring puckers that cause a kink in the polymer chain, i.e. non-^2S_O (pink solid line; most probable end-to-end distance is 73.0 Å) and ^2S_O conformations (green solid line; most probable end-to-end distance is 68.5 Å) and the average of all four runs in the full MD-generated ensemble (black dashed line; most probable end-to-end distance is 68.5 Å); probabilities were calculated for end-to-end distances sorted into 0.5 Å bins.

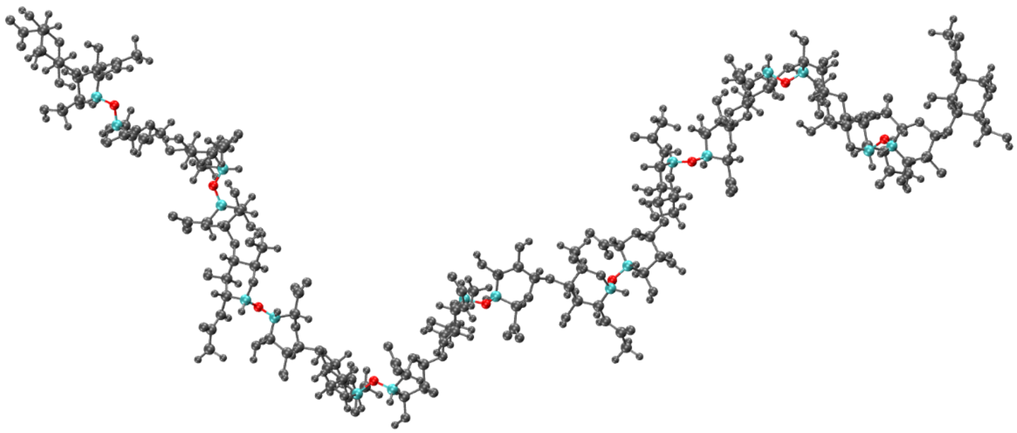

Figure S31. Snapshot of non-sulfated heparan 20-mer from MD simulation; GlcNAcα1-4IdoA linkages are highlighted; linker oxygen atoms in red and carbon atoms (GlcNAc C_1 and IdoA C_4) in cyan.

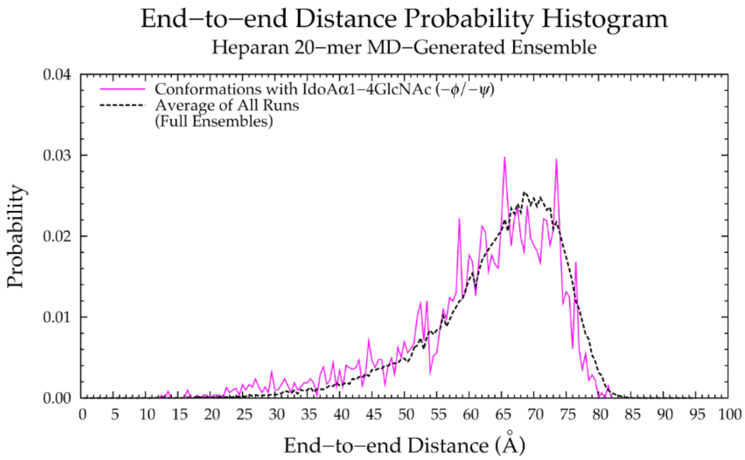

Figure S32. End-to-end distance probability distribution of MD-generated non-sulfated heparan 20-mer conformations with IdoA*α*1-4GlcNAc linkages with -*ϕ*, -*ψ* dihedrals aggregated across all four MD runs (pink solid line; top two most probable end-to-end distances are 65.5 Å and 73.5 Å); these data were compared to the average end-to-end distance distribution of all snapshots in all four MD runs (black dashed line; most probable end-to-end distance is 68.5 Å).

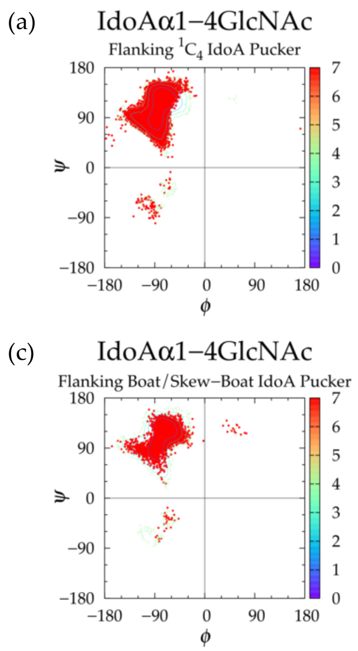

Figure S33. Scatterplots for dihedrals ϕ and ψ of IdoAα1-4GlcNAc linkages flanking different IdoA conformations in the MD-generated non-sulfated heparan 20-mer ensemble: (a) $^{1}C_4$, (b) $^{2}S_O$, (c) boat/skew-boat (non-$^{2}S_O$), and (d) $^{4}C_1$; contour lines come from corresponding aggregated MD-generated $\Delta G(\phi, \psi)$ data.

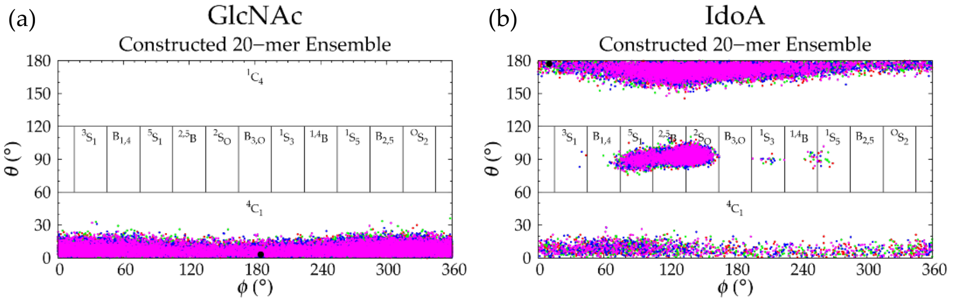

Figure S34. Cremer–Pople data for (a) GlcNAc and (b) IdoA in the constructed non-sulfated heparan 20-mer ensemble; each of the 4 runs is represented by different color and the force-field geometry is represented by a single large black dot; each run contains 10,000 parameter sets.

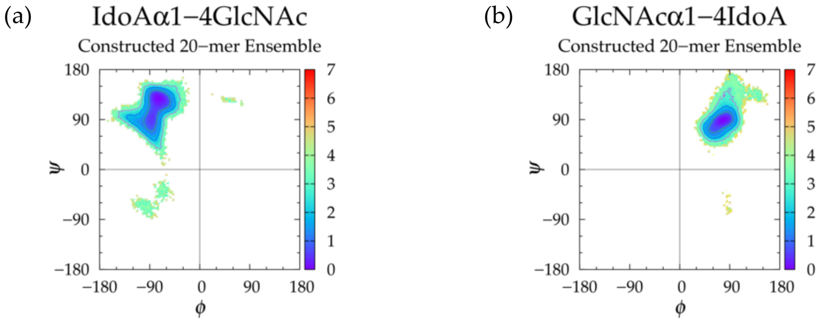

Figure S35. $\Delta G(\phi,\ \psi)$ in the constructed non-sulfated heparan 20-mer ensemble for aggregated (a) IdoAα1-4GlcNAc and (b) GlcNAcα1-4IdoA glycosidic linkage data; contour lines every 1 kcal/mol.

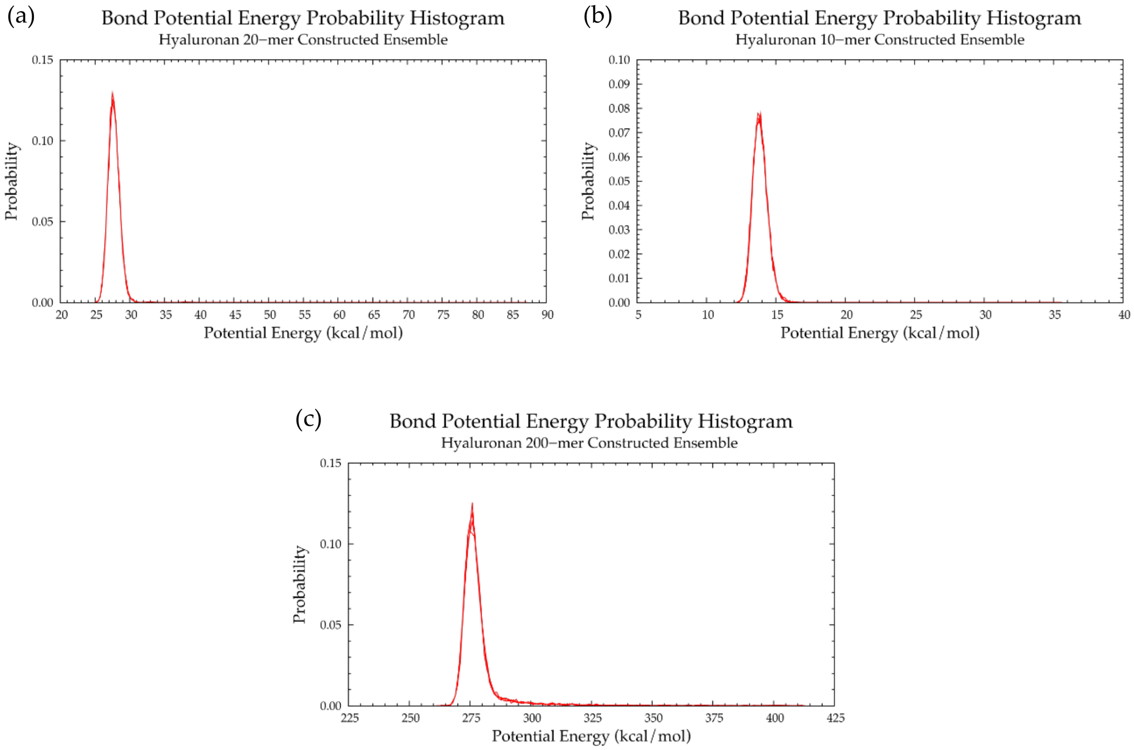

Figure S36. Bond potential energy probability distributions from constructed ensembles of hyaluronan (a) 20-mer (cutoff = 128.5 kcal/mol), (b) 10-mer (cutoff = 112.8 kcal/mol), and (c) 200-mer (cutoff = 412.2 kcal/mol).

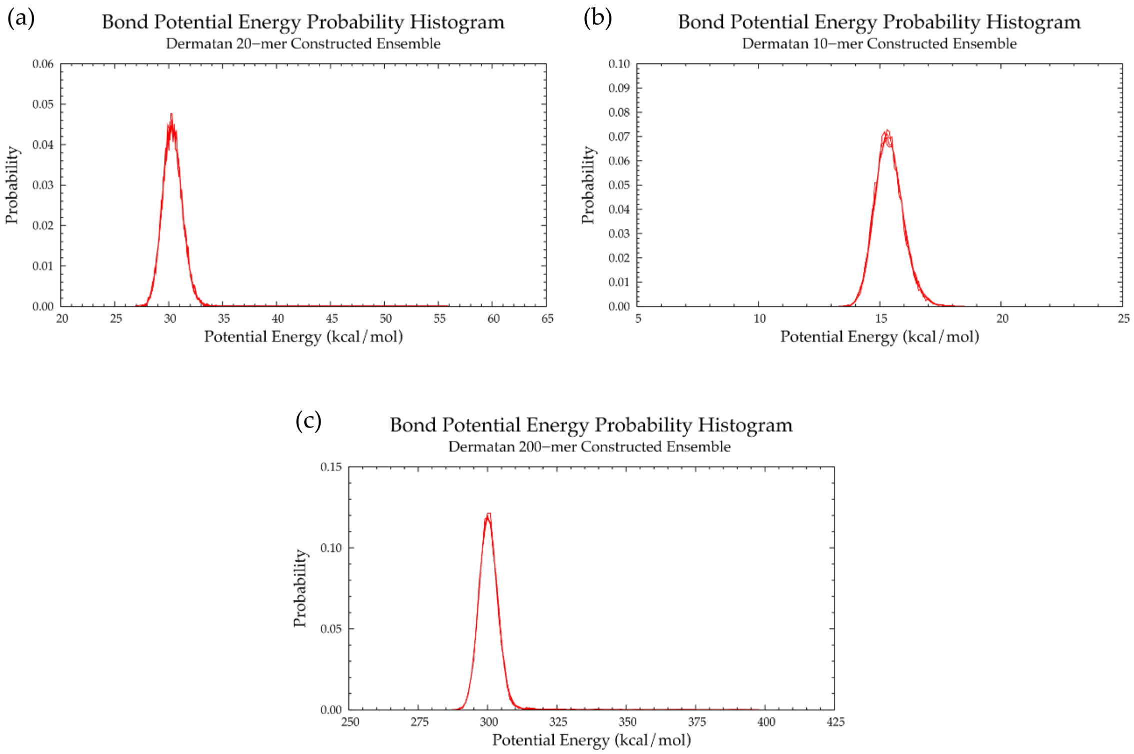

Figure S37. Bond potential energy probability distributions from constructed ensembles of non-sulfated dermatan (a) 20-mer (cutoff = 131.9 kcal/mol), (b) 10-mer (cutoff = 117.5 kcal/mol), and (c) 200-mer (cutoff = 397.7 kcal/mol).

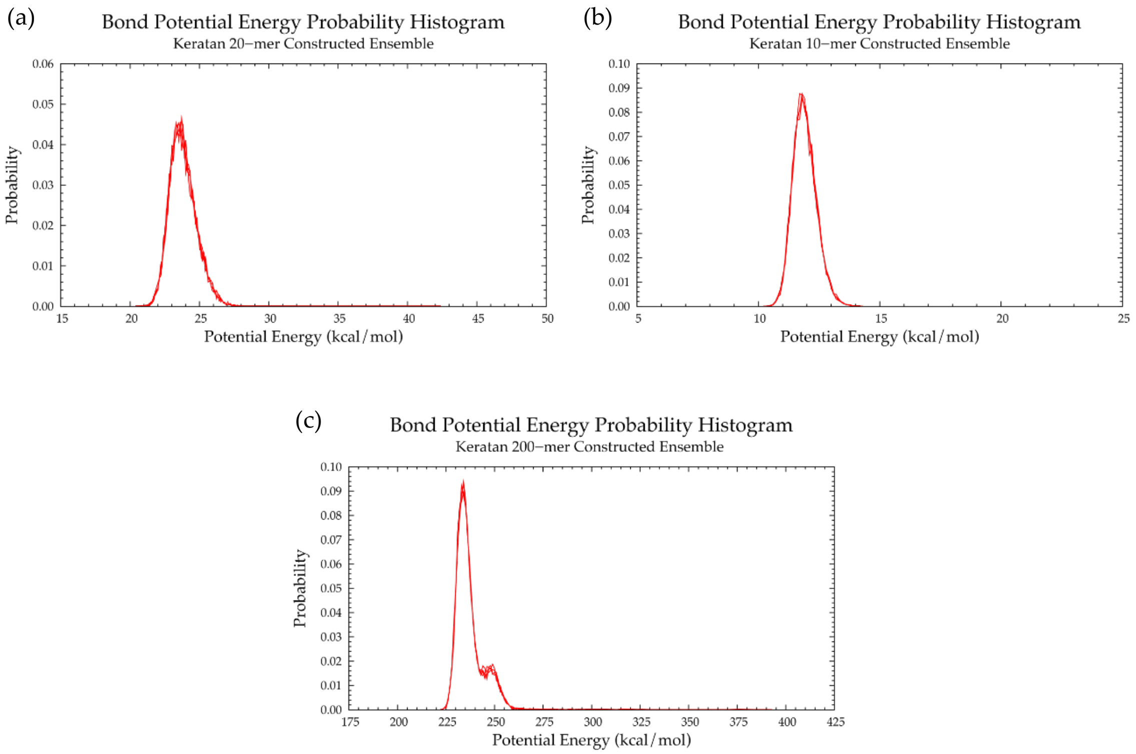

Figure S38. Bond potential energy probability distributions from constructed ensembles of non-sulfated keratan (a) 20-mer (cutoff = 126.3 kcal/mol), (b) 10-mer (cutoff = 111.5 kcal/mol), and (c) 200-mer (cutoff = 397.3 kcal/mol).

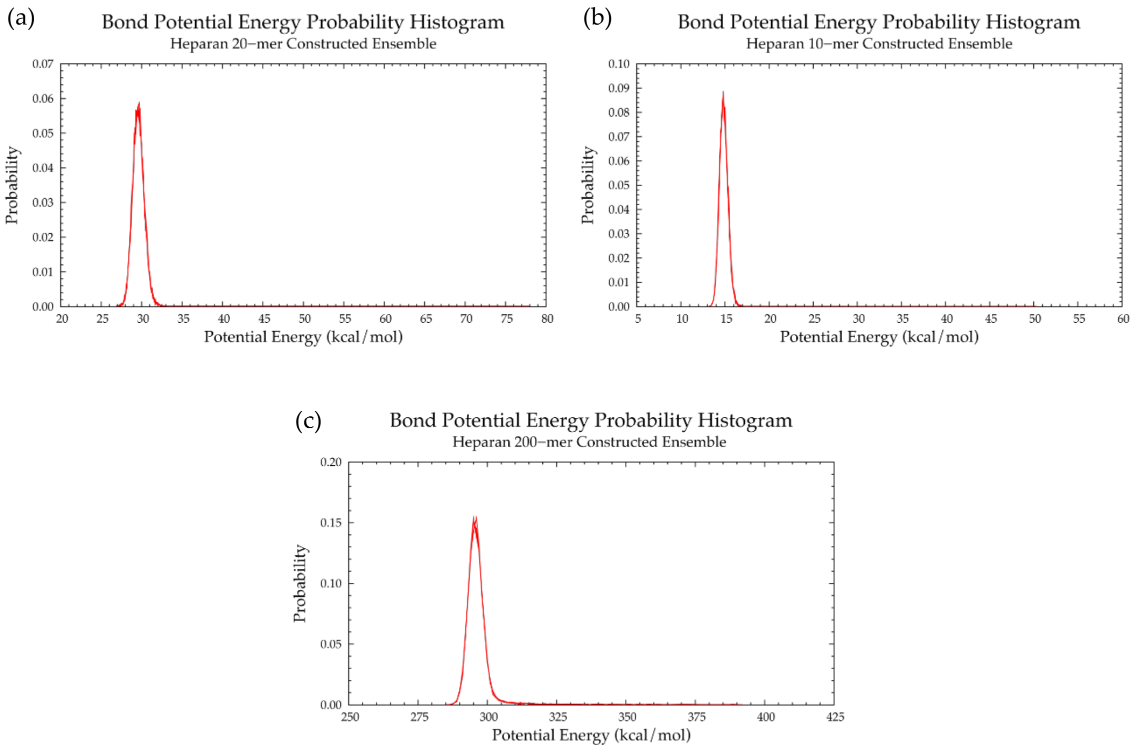

Figure S39. Bond potential energy probability distributions from constructed ensembles of non-sulfated heparan (a) 20-mer (cutoff = 130.3 kcal/mol), (b) 10-mer (cutoff = 115.8 kcal/mol), and (c) 200-mer (cutoff = 391.5 kcal/mol).
